# Isomeric triazines exhibit unique profiles of bioorthogonal reactivity†
†Electronic supplementary information (ESI) available: Synthetic procedures, protein expression procedures, computational details, NMR and mass spectra. See DOI: 10.1039/c9sc01427f


**DOI:** 10.1039/c9sc01427f

**Published:** 2019-08-21

**Authors:** David N. Kamber, Sean S. Nguyen, Fang Liu, Jeffrey S. Briggs, Hui-Wen Shih, R. David Row, Zane G. Long, K. N. Houk, Yong Liang, Jennifer A. Prescher

**Affiliations:** a Department of Chemistry , University of California , Irvine , California 92697 , USA . Email: jpresche@uci.edu; b Department of Chemistry and Biochemistry , University of California , Los Angeles , California 90095 , USA; c State Key Laboratory of Coordination Chemistry , Jiangsu Key Laboratory of Advanced Organic Materials , School of Chemistry and Chemical Engineering , Nanjing University , Nanjing 210023 , China . Email: yongliang@nju.edu.cn; d Department of Molecular Biology & Biochemistry , University of California , Irvine , California 92697 , USA; e Department of Pharmaceutical Sciences , University of California , Irvine , California 92697 , USA

## Abstract

Expanding the scope of bioorthogonal reactivity requires access to new and mutually compatible reagents. We report here that 1,2,4-triazines can be tuned to exhibit unique reaction profiles with biocompatible strained alkenes and alkynes. Computational analyses were used to identify candidate orthogonal reactions, and the predictions were experimentally verified. Notably, 5-substituted triazines, unlike their 6-substituted counterparts, undergo rapid [4 + 2] cycloadditions with a sterically encumbered strained alkyne. This unique, sterically controlled reactivity was exploited for dual bioorthogonal labeling. Mutually orthogonal triazines and cycloaddition chemistries will enable new multi-component imaging applications.

## Abstract 2

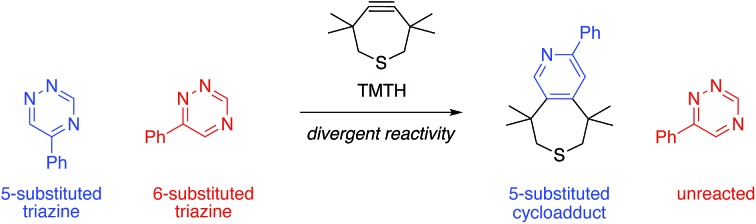
Isomeric triazines can be tuned to exhibit unique reaction profiles with biocompatible strained alkenes and alkynes.

## 

Bioorthogonal chemistries have been used extensively to tag biomolecules in complex environments.^1^ The success of these transformations is critically dependent on the stabilities of the reagents in cells and tissues. At the same time, the reagents must be robustly and singularly reactive with complementary probes.^2^ This chemical paradox has often frustrated efforts to develop reagents that exhibit selective reactivity, let alone multiple reactions that function in concert. In fact, there are only a handful of bioorthogonal reaction pairs that can be used simultaneously.^3^–^16^

We are developing “privileged” scaffolds that not only meet the requirements for bioorthogonality, but are also compatible with existing reagents to enable tandem application.^4^,^17^ Such mutually orthogonal reagents are in demand for chemical tagging of multiple biomolecules.^5^–^14^ We recently reported that a 6-substituted 1,2,4-triazine constitutes a new privileged scaffold (Fig. 1A).^18^ This motif reacts with *trans*-cyclooctene (TCO) *via* inverse electron-demand Diels–Alder (IED-DA) cycloaddition. The rate of this cycloaddition can be further improved by scaffold tuning.^19^ 6-Substituted triazines are also remarkably inert to other biological functionality. The enhanced stability of the triazine enabled its direct application in genetic code expansion and recombinant protein production.^18^ Additionally, while 6-substituted triazines react efficiently with TCO, they do not react with other bioorthogonal alkenes, including norbornene and cyclopropene.^18^ This result is in sharp contrast to related tetrazine motifs, which react vigorously with a variety of alkenes and alkynes.^12^,^13^,^20^–^28^

**Fig. 1:**
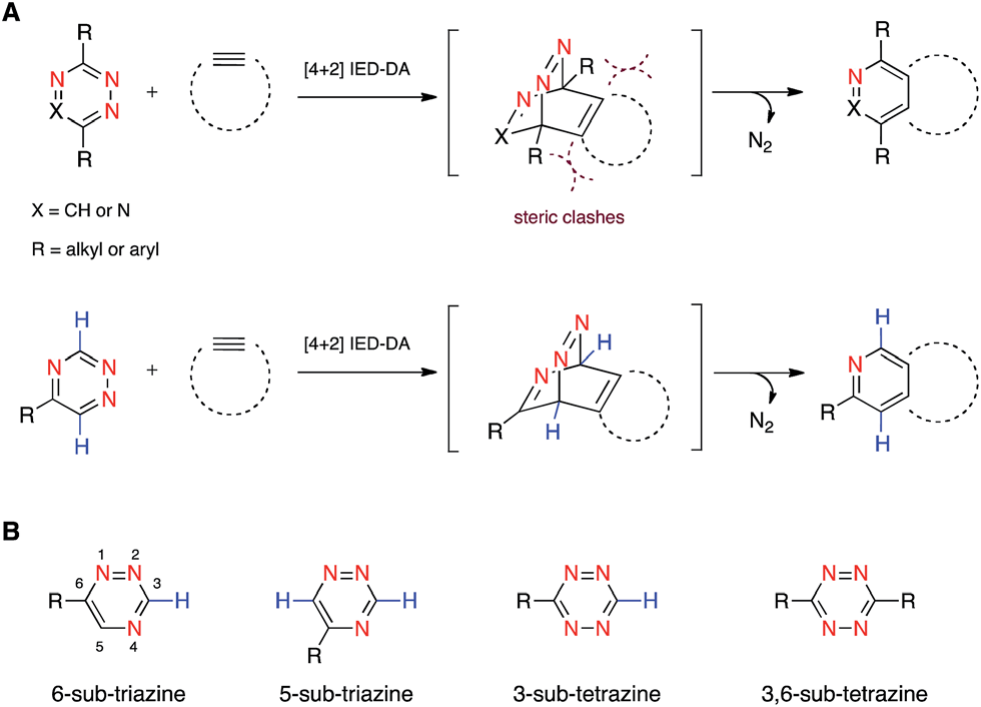
Inverse electron-demand Diels–Alder (IED-DA) reactions. (A) Triazine (X = CH) and tetrazine (X = N) scaffolds react with strained alkynes to form stable cycloadducts. (B) Isomeric 1,2,4-triazines and 1,2,4,5-tetrazines examined in this work.

To expand upon the unique features of triazines, we aimed to synthesize and examine the reactivities of alternatively substituted rings. 1,2,4-Triazines react with dienophiles to form new bonds across C3 and C6.^18^,^29^ The regioselectivity of this addition could potentially be exploited for orthogonal reaction development: 6-substituted triazines would be less likely to react with bulky dienophiles than their 5-substituted counterparts (Fig. 1B). Thus, different substitution patterns could effectively “tune” triazine reactivity and promote selective cycloaddition. Similar tactics have been used to develop mutually compatible reactions with tetrazines.^10^ For example, mono-substituted tetrazines react rapidly even with sterically encumbered cyclooctynes.^30^,^31^ By contrast, disubstituted tetrazines do not react with similar alkynes due to steric clashes in the transition states.^32^

To examine whether differentially substituted triazines could provide mutually orthogonal reactions, we first used density functional theory (DFT) calculations at the M06-2X/6-311+G(d,p)//M06-2X/6-31G(d) level to evaluate the reactivities of model probes.^33^–^36^ Isomeric triazines and substituted tetrazines were included in the analyses, along with a panel of bioorthogonal strained dienophiles (Table 1).^37^–^41^ The triazine motifs differed in their substitution patterns, with phenyl groups positioned at C3 and C6 (the sites of new bond formation in cycloaddition reactions), or C5. In agreement with our previous work, triazines were predicted to react readily with TCO, although more slowly than their tetrazine counterparts. Minimal or no triazine reactivity was predicted with other strained alkenes, including 1-methylcyclopropene and norbornene. Structurally related tetrazines, by contrast, harbor much lower LUMO+1 energies and were predicted to react robustly with a variety of strained alkenes.^42^

**Table 1:** DFT-computed activation free energies (kcal mol^–1^) and predicted rate constants (M^–1^ s^–1^) for tetrazine/triazine cycloadditions with strained dienophiles. Predicted rate constants (in water at 25 °C) range from 10^–10^ to 10^3^. Red designates slow (10^–10^ to 10^–4^), yellow designates intermediate (10^–3^ to 10^–2^), and green designates fast (10^–2^ to 10^3^) rates

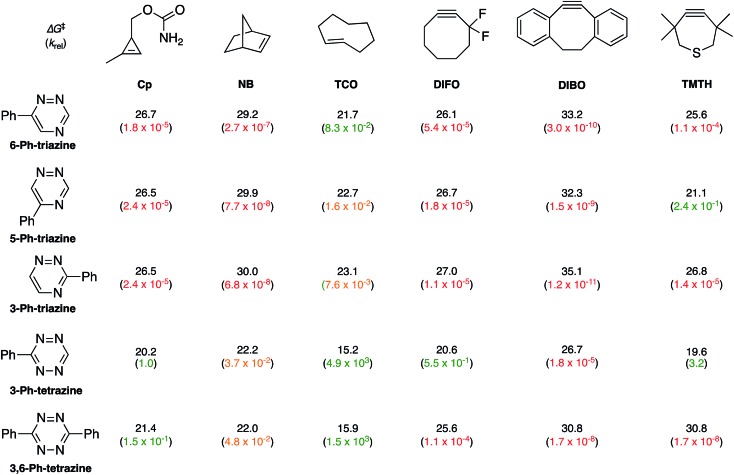

When evaluating strained alkynes, the reactivity profiles of the triazine isomers diverged. The 5-substituted scaffold was predicted to react with tetramethylthiacycloheptyne (TMTH, **9**), a sterically encumbered cycloalkyne developed by the Bertozzi group.^43^ The 3- and 6-substituted isomers were predicted to exhibit diminished reactivity with TMTH due to steric clashes at the reactive centers. Calculations further suggested that none of the triazine isomers would react efficiently with other strained alkynes, including DIBO and DIFO (molecules with lower HOMO energies), setting the stage for orthogonal reaction development.

To validate the computational predictions, we synthesized the panel of reagents shown in Table 2. The triazine and tetrazine scaffolds were incubated with a variety of strained alkenes and alkynes, and the reactions were monitored by ^1^H-NMR spectroscopy (Fig. S1–S10†). The measured bimolecular rate constants closely matched those predicted by DFT calculations (Table 2). The 6-substituted triazine (**1**) reacted at a reasonable rate with TCO, but no other strained alkene (**4–6**). The 5-phenyl isomer (**2**) was also efficiently ligated with TCO, but reacted minimally with cyclopropene **4** (Fig. S3–S5†). Importantly, the most tantalizing prediction—robust reactivity between TMTH (**9**) and 5-phenyl-1,2,4-triazine (**2**)—was also validated (Fig. S9–S11†). The reaction proceeded with a rate constant of *k*_2_ = 0.22 ± 0.01 M^–1^ s^–1^. This rate is on par with many commonly used distortion-accelerated azide–alkyne cycloadditions.^2^,^44^–^47^ Under similar conditions, no reactivity was observed between 6-substituted triazine (**1**) and TMTH (**9**) (Fig. S12†). Even with a more reactive triazine (**S3**), minimal reactivity was observed (Fig. S13†). The selective reactivity of **2** with TMTH (**9**) was further showcased in a competition experiment (Fig. 2). When the isomeric triazines were combined in equimolar amounts and treated with excess TMTH (**9**), only the 5-substituted triazine (**2**) was consumed (Fig. 2A).

**Table 2:** Second order rate constants (M^–1^ s^–1^) for tetrazine and triazine cycloadditions with strained dienophiles. All reactions were conducted at 25 °C and monitored *via*^1^H-NMR spectroscopy. Reactions were run in CD_3_CN containing 10–50% *d*-PBS unless otherwise stated. N.R. indicates no reaction after 24 h and a corresponding rate constant (*k*_2_) < 10^–4^ M^–1^ s^–1^. The color code is defined in Table 1

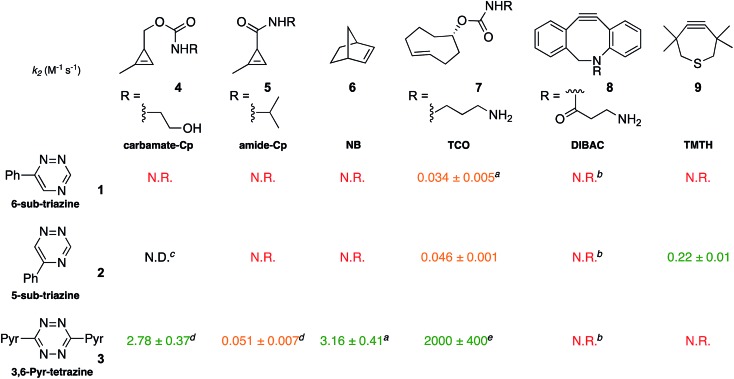

^*a*^Rate determined in ref. 18.

^*b*^Reactions were run in 40% CD_3_OD in CD_3_CN.

^*c*^Rate not determined. After 24 h, 15% conversion to an unisolable product was observed.

^*d*^Rate determined in ref. 12.

^*e*^The rate constant as measured with unsubstituted TCO in ref. 20.

**Fig. 2:**
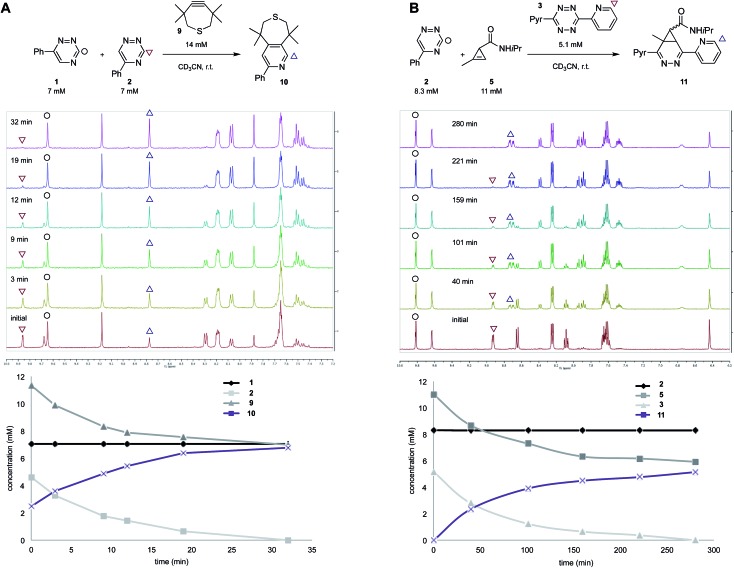
Isomeric triazines exhibit unique bioorthogonal reactivities. (A) 5-Phenyl-triazine (**2**) reacts exclusively with TMTH. The reaction was monitored by ^1^H-NMR spectroscopy (top). The plot of reaction progress over time is shown below. (B) 5-Phenyl-1,2,4-triazine (**2**) can be used in combination with disubstituted tetrazine and cyclopropene scaffolds. The reagents were combined and monitored by ^1^H-NMR spectroscopy (top). The plot of reaction progress over time is shown below.

The unique reactivity profile of 5-phenyl triazine (**2**) suggested immediate opportunities for mutually orthogonal reaction development. While triazine **2** reacts quickly with TMTH (**9**), this isomer is refractory to ligation with bioorthogonal cyclopropenes (scaffolds known to react robustly with tetrazines, Fig. S14 and S15†). Since tetrazines and cyclopropenes are both inert to TMTH and triazines (Fig. S16†), these reagents could be exploited for dual [4 + 2] cycloaddition. To examine this possibility, triazine **2** was first mixed with cyclopropene **5** and tetrazine **3**. Over the course of the reaction, the concentration of triazine **2** remained constant, while cyclopropene **5** and tetrazine **3** were consumed (Fig. 2B). When all four model reagents were combined (2.5 mM, 1 equiv.), the two expected cycloadducts were observed (Fig. 3, S17 and S18†). Similar results were obtained using a more reactive cyclopropene in the mixture (Fig. S19†). To our knowledge, these are the first examples of [4 + 2] IED-DA cycloadditions that can be used concurrently without cross-reactivity.^3^ Bonger and colleagues recently reported a pair of [4 + 2] cycloadditions for dual labeling, but precise reagent stoichiometries were required to prevent off-target reactions.^13^

**Fig. 3:**
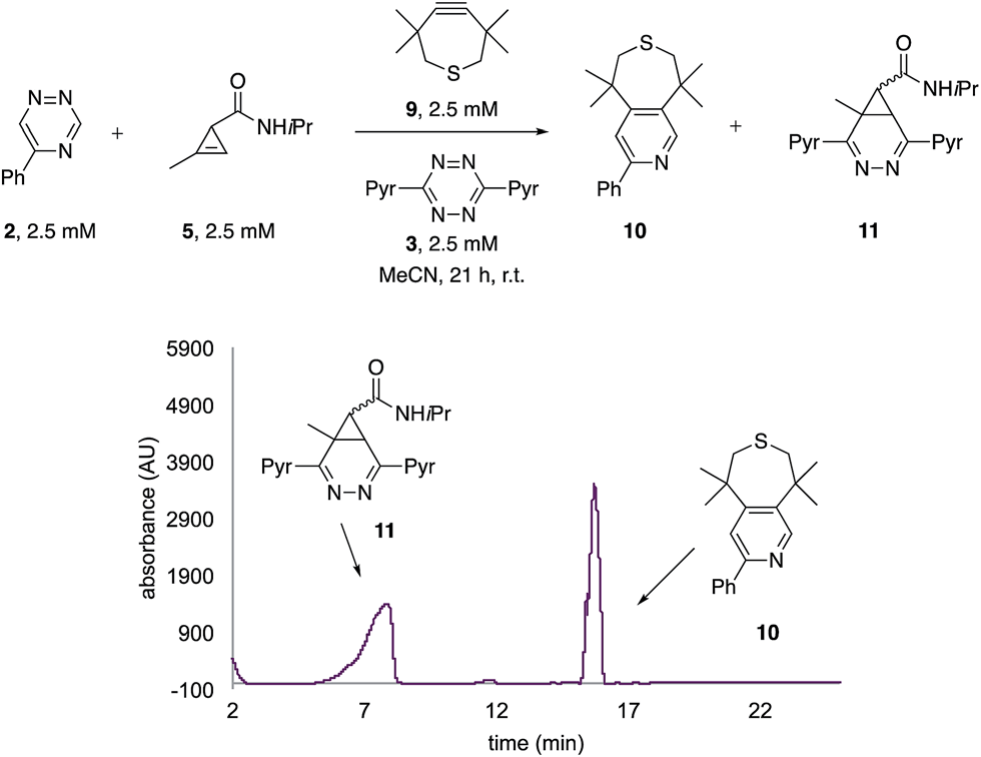
Orthogonal [4 + 2] cycloadditions. All reagents (2.5 mM) were combined and the reactions were monitored by HPLC (210 nm). Two distinct cycloadducts were observed.

The mutual orthogonality of the reagents from this work arises from the interplay of intrinsic reactivities and steric factors.^18^,^48^ Tetrazines are more reactive than triazines with bioorthogonal dienophiles due to the lower LUMO+1 energies of the π-systems.^42^,^49^,^50^ This intrinsic order of reactivity is manifested in the “Distortion/Interaction-Activation Strain Model”^51^ analysis shown in Fig. 4. The sterically unhindered cyclopropene reacts most slowly with **2**, slightly faster with **1**, and fastest with the tetrazine analog. These differences in reactivity arise from increased interaction energies that parallel the increasing electrophilicity across the series. The same behavior is observed with other sterically unhindered dienophiles, such as the highly reactive TCO. With a sterically hindered dienophile (*e.g.*, TMTH), though, the opposite trend is observed. The more hindered tetrazine reacts most slowly with TMTH (**9**), while the less hindered 5-phenyl triazine (**2**) ligates most rapidly. TMTH reactivity is mainly controlled by distortion energies that are strongly influenced by steric considerations. Balancing electronic interaction energies (that enhance reactivity) with steric effects (that increase distortion energies and thus decrease reactivity) is a general approach for developing mutually orthogonal bioorthogonal reactions.^11^,^36^,^48^

**Fig. 4:**
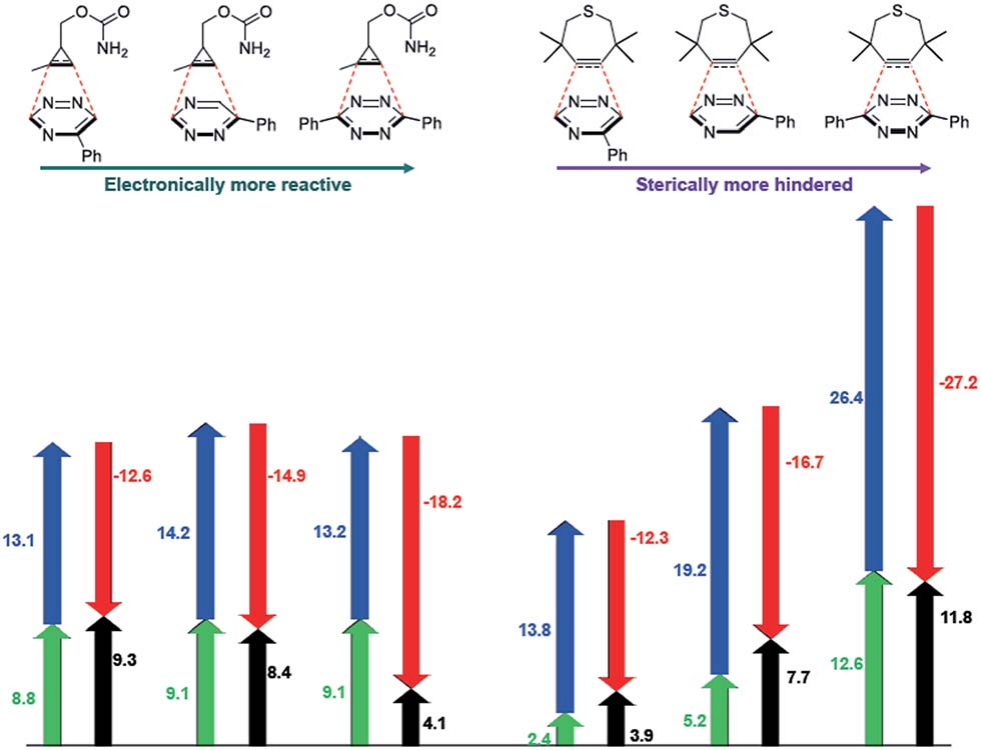
Distortion/interaction analysis of factors controlling mutually orthogonal cycloadditions. Black arrows are activation potential energies, green and blue arrows are distortion energies of dienophile and diene, respectively, and red arrows are interactions energies. All values are given in kcal mol^–1^.

Encouraged by the computational and experimental analyses, we examined whether the orthogonal cycloadditions could be used in more complex environments. Toward this end, we attempted the labeling reactions in concert with two model proteins (GFP and NanoLuciferase, Nluc). We attached a single 5-substituted triazine to Nluc using cysteine-maleimide chemistry (Fig. S20 and Scheme S1†). In this case, Nluc was engineered to harbor a single cysteine at residue 180.^52^ The resulting conjugate (**Nluc-Triazine**) was readily ligated with TMTH (**9**, Fig. S21†). We further prepared a cyclopropene-GFP (**GFP-Cp**) conjugate *via* genetic code expansion (Fig. S21†).^53^ The model proteins were combined 1 : 1 in PBS (pH 7.3, 2 μM final concentration). The mixture was then treated with TMTH (**9**) and tetrazine **3**. After 3 h, full consumption of the starting proteins was observed *via* mass spectrometry (Fig. 5, S21 and S22†). No cross-reactivities were observed, suggesting that the cycloadditions can be used to label two biological targets simultaneously. The orthogonal [4 + 2] cycloadditions could also be performed in the presence of lysate, conditions that mimic cellular environments (Fig. S23†). The cycloadditions were also compatible with polar bioorthogonal reactants, enabling simultaneous, triple-component ligations (Fig. S24 and S25†). Collections of three mutually orthogonal reactions are rare.^54^,^55^

**Fig. 5:**
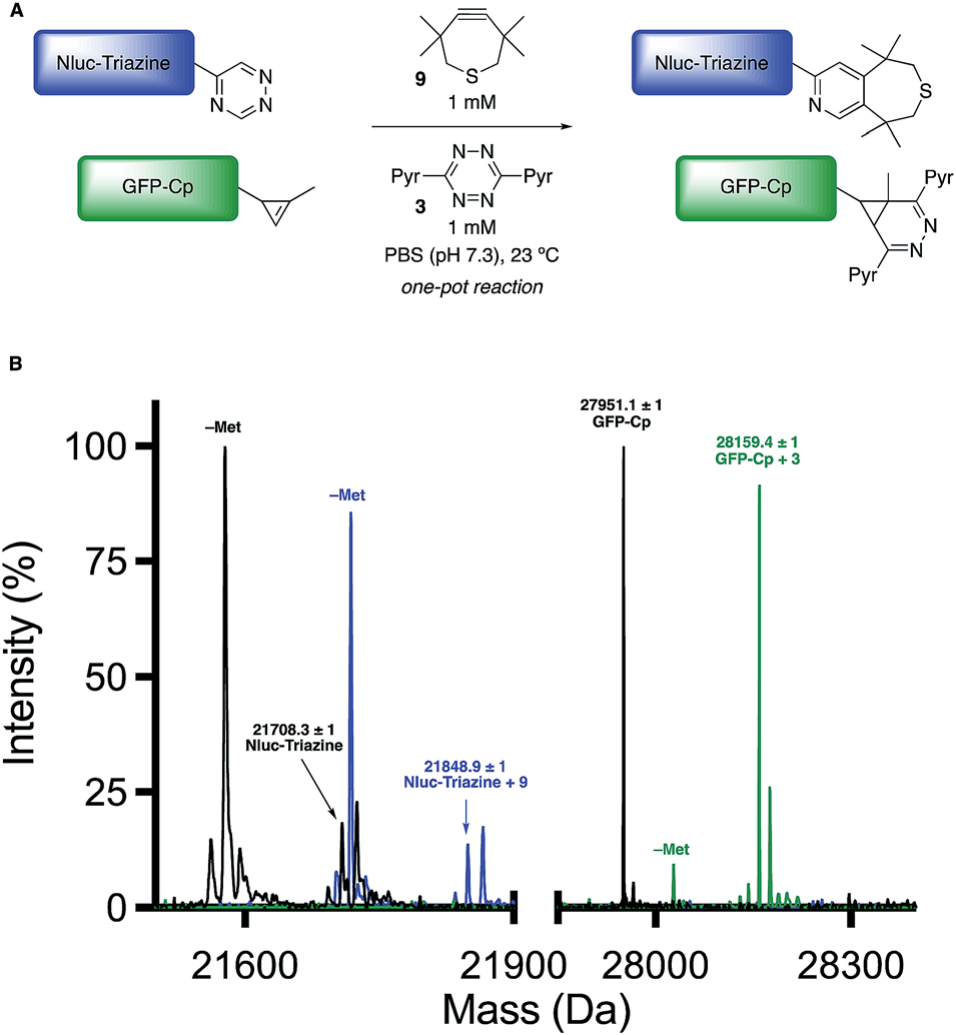
Orthogonal [4 + 2] cycloadditions enable dual protein labeling. (A) **Nluc-Triazine** and **GFP-Cp** were mixed 1 : 1 in PBS (pH 7.3, 2 μM), and subsequently treated with TMTH (**9**) and tetrazine **3** (1 mM). (B) Quantitative conversion to the expected cycloadducts was observed after 3 h *via* mass spectrometry. N-Terminal methionine cleavage (–Met) was observed for both proteins and their respective cycloadducts.

In conclusion, we identified isomeric triazine scaffolds that exhibit unique cycloaddition profiles. Computational and experimental analyses of triazine reactivity were performed. Isomeric triazines were found to react robustly with TCO, but only the least sterically encumbered isomers (5-substituted) reacted with other dienophiles. Notably, 5-substituted 1,2,4-triazines reacted efficiently with TMTH, one of the most sterically encumbered strained alkynes reported to date. The cycloaddition was successfully used in combination with another popular IED-DA reaction, the tetrazine ligation with 1-methylcyclopropene. These mutually compatible reactions can be used in tandem to tag protein targets in biologically relevant environments. Future work will address the need for functional TMTH conjugates. TMTH has been historically difficult to outfit with fluorophores and other reporter groups, although new strategies for derivatization are being pursued. A panel of easily accessible reagents will further enhance multi-component labeling applications.

## Conflicts of interest

There are no conflicts to declare.

## Supplementary Material

Supplementary information
