# Supplementary material for: Isomeric triazines exhibit unique profiles of bioorthogonal reactivity
Source: Chem Sci. 2019 Aug 21;10(39):9109–14. doi: 10.1039/c9sc01427f (PMC6910137; doi:10.1039/c9sc01427f)
Supplement: Supplementary file 1 [file SC-010-C9SC01427F-s001.pdf]

Electronic Supplementary Material (ESI) for Chemical Science.
This journal is © The Royal Society of Chemistry 2019

Isomeric 1,2,4-triazines exhibit unique profiles of bioorthogonal reactivity

David N. Kamber[1], Sean S. Nguyen[1], Fang Liu[4,6], Jeffrey S. Briggs[1], Hui-Wen Shih[1], R David Row[1], Zane G. Long[1], Kendall N. Houk[4-5], Yong Liang*[6], and Jennifer A. Prescher*[,1-3]

Departments of [1]Chemistry, [2]Molecular Biology & Biochemistry, and [3]Pharmaceutical Science, University of California, Irvine, CA 92697, USA; Departments of [4]Chemistry and Biochemistry, and [5]Chemical and Biomolecular Engineering, University of California, Los Angeles, CA 90095, USA; [6]State Key Laboratory of Coordination Chemistry, Jiangsu Key Laboratory of Advanced Organic Materials, School of Chemistry and Chemical Engineering, Nanjing University, Nanjing 210023, China

TABLE OF CONTENTS

Materials and Methods S2

Procedures S2-S8

Scheme S1 S3

Computational Studies S8-S25

References S25-S26

Supplemental Figures 1-2 S27-S28

Supplemental Figures 3-8 S29-S34

Supplemental Figures 9-13 S35-S39

Supplemental Figures 14-19 S40-S45

Supplemental Figures 20-22 S46-S48

Supplemental Figures 23-25 S49-S52

NMR Spectra S53-S68

Materials and Methods

Rate studies

The reactions between tetrazines/1,2,4-triazines and strained dienophiles **4-9** were monitored by ^{1}H-NMR spectroscopy. A solution of the strained dienophile (0.12–0.3 mL of a 20–50 mM solution) was added to a solution of the appropriate 1,2,4-triazine or tetrazine in CD_3CN (0.12–0.24 mL, 20–50 mM). The reactions were then diluted to a final volume of 0.6 mL. The final concentrations of all reactants were 5-10 mM and reactions were monitored for at least 24 h. All reactions were carried out at 25 °C. Representative spectra and experimental conditions for each reaction are shown in the accompanying figures.

General synthetic procedures

Compounds **1**[1], **2**[2], **4**[3], **5**[4], **9**[5], **11**[4], **12**[4], **13**[4], **S1**[1], **S3**[1], **S4**[6], **S8**[7], **S10**[8], **S12**[9], **S14**[10] and **S15**[11] were synthesized as previously reported. All other reagents were obtained from commercial sources and used without further purification. Reactions were run under ambient conditions, unless otherwise indicated. Tetrahydrofuran (THF), diethyl ether (Et_2O), dichloromethane (CH_2Cl_2), dimethylformamide (DMF), and acetonitrile (MeCN) were degassed with argon and run through two 4 x 36 inch columns of anhydrous neutral A-2 (8 x 14 mesh; LaRoche Chemicals; activated under a flow of argon at 350 °C for 12 h). Thin-layer chromatography was performed using Silica Gel 60 F_{254}-coated glass plates (0.25 mm thickness), and visualization was performed with $KMnO_4$ stain and/or UV irradiation. Chromatography was accomplished with 60 Å (240–400 mesh) silica gel, commercially available from Sorbent Technologies. Organic solutions were concentrated under reduced pressure using a Büchi rotary evaporator. HPLC purifications were performed on a Varian ProStar equipped with 325 a Dual Wavelength UV-Vis detector. Analytical runs were performed using an Agilent C18 Scalar column (4.6 x 150 mm, 5 µm) with a 1 mL/min flow rate, and visualized with 210 nm wavelength. NMR spectra were collected on a Bruker DRX400 (400 MHz ^{1}H, 100 MHz ^{13}C, 376.5 MHz ^{19}F), a Bruker DRX500 equipped with a cryo probe (500 MHz ^{1}H, 125.7 MHz ^{13}C), or a Bruker AVANCE600 equipped with a cryo probe (600 MHz ^{1}H, 150 MHz ^{13}C). All spectra were collected at 298 K. High-resolution mass spectrometry was performed by the University of California, Irvine Mass Spectrometry Center. Protein mass spectrometry (ESI-MS) experiments were performed on a Waters Xevo LV-G2 Q-TOF instrument.

Synthetic procedures

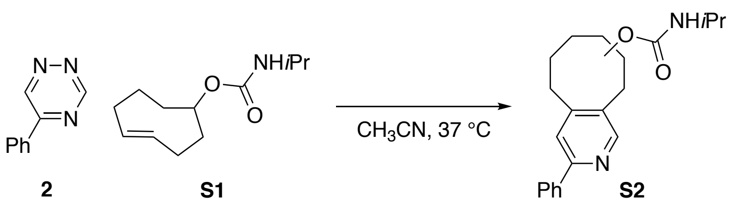

2 **S1** **S2**

***N*-Isopropyl-2-(3-phenyl-5,6,7,8,9,10-hexahydrocycloocta[*c*]pyridin-8-yl)acetamide (S2)**: To a vial containing TCO-isopropylamine **S1** (16.0 mg, 0.0757 mmol) was added a solution of triazine **2** (12.0 mg, 0.0763 mmol) in 1 mL MeCN. The vial was placed in a

37 °C incubator for 3 d to facilitate air-oxidation to the pyridine adduct. After 3 d, the crude product was concentrated *in vacuo* and purified by flash column chromatography (eluting with 10–20% EtOAc in CH_2Cl_2) to provide aromatized cycloadduct **S2** as a mixture of two regioisomers (11.0 mg, 43%) as a light-yellow oil. ^1H NMR (400 MHz, $CDCl_3$): δ 8.44 (s, 1H), 8.40 (s, 1H), 7.98 (apparent d, J = 7.4 Hz, 4H), 7.51–7.38 (m, 8H), 4.58–4.39 (m, 3H), 3.77 (m, 2H), 2.84–2.76 (m, 6H), 2.20–1.48 (m, 17H), 1.14 (m, 13H). ^{13}C NMR (125 MHz, $CDCl_3$) δ 156.2, 156.0, 150.1, 150.0, 149.8, 139.5, 139.4, 134.5, 134.4, 128.7, 128.7, 128.6, 126.8, 120.8, 74.7, 74.5, 43.0, 37.3, 36.6, 33.0, 31.8, 29.8, 29.1, 28.9, 27.9, 27.2, 25.9, 23.1. HRMS (ESI^+) m/z calcd. for $C_{21}H_{26}N_2O_2Na$ $[M+Na]^+$ 361.1892, found 361.1882.

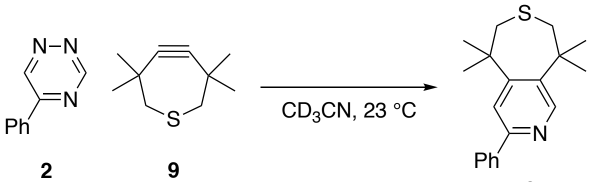

2 9 10

5,5,9,9-Tetramethyl-3-phenyl-5,6,8,9-tetrahydrothiepino[4,5-*c*]pyridine (10):
Triazine **2** and TMTH **9** (0.3 mL of a 20 mM solution) were mixed in a 1:1 ratio in CD_3CN and placed in 3 separate NMR tubes. After the reaction was complete, the solutions were combined and concentrated *in vacuo* to provide cycloadduct **10**. ^1H NMR (400 MHz, CD_3CN): δ 8.73 (s, 1H), 8.03 (d, J = 7.6 Hz, 2H), 7.83 (s, 1H), 7.49–7.39 (m, 3H), 2.87 (s, 2H), 2.86 (s, 2H), 1.58 (s, 6H), 1.57 (s, 6H). ^{13}C NMR (125 MHz, CD_3CN) δ 157.2, 153.6, 151.0, 141.5, 139.1, 128.7, 126.6, 119.7, 42.9, 41.8, 41.4, 41.1, 32.0, 31.7. HRMS (ESI^+) m/z calcd. for $C_{19}H_{24}NS$ $[M+H]^+$ 298.1629, found 298.1639.

Scheme S1. Synthesis of triazine-maleimide probe **S9**.

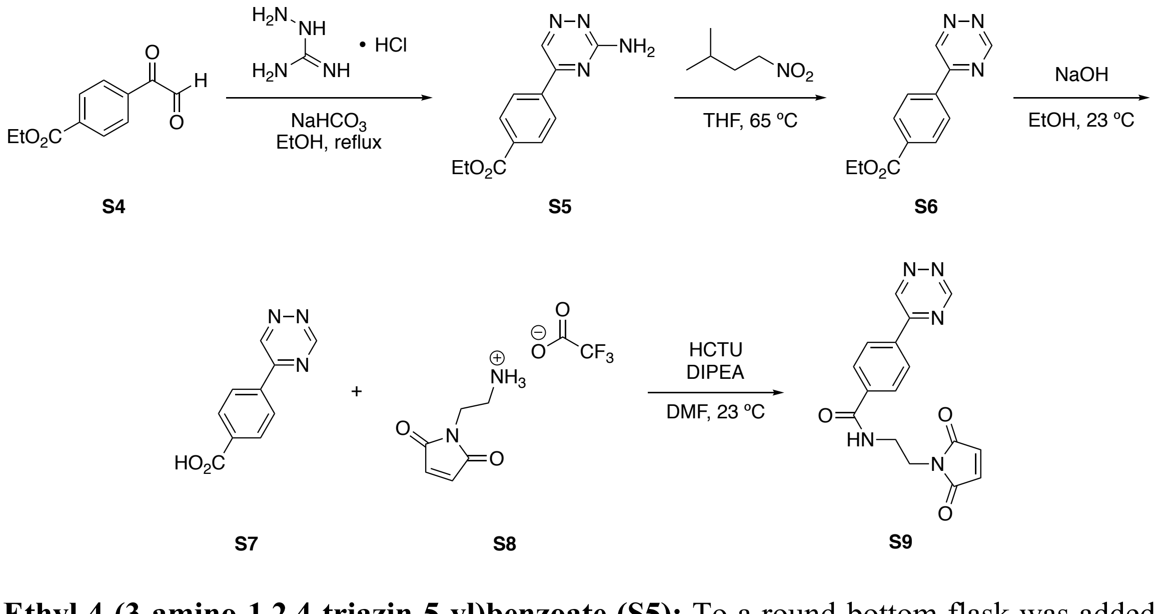

S4 S5 S6 S7 S8 S9

Ethyl 4-(3-amino-1,2,4-triazin-5-yl)benzoate (S5): To a round-bottom flask was added **S4** (0.912 g, 4.42 mmol) and EtOH (60 mL). Aminoguanidine hydrochloride (0.491 g,

4.44 mmol) and $NaHCO_3$ (1.12 g, 13.3 mmol) were added, and the mixture was stirred at reflux overnight. The resulting green solution was diluted with H_2O (40 mL) and EtOAc (40 mL), and the organic layer was washed with H_2O (3 x 75 mL). The aqueous layer was extracted with EtOAc (3 x 50 mL), and the combined organic layers were dried with $MgSO_4$. The solution was filtered and concentrated *in vacuo*, then dry-loaded on silica and purified by flash chromatography (eluting with 10–20% EtOAc in CH_2Cl_2) to give **S5** (73.8 mg, 7%) as a bright yellow solid. ^{1}H NMR (400 MHz, $(CD_3)_2SO$): δ 9.28 (s, 1H), 8.31 (d, J = 7.6 Hz, 2H), 8.12 (d, J = 7.5 Hz, 2H), 7.38 (br s, 2H), 4.36 (q, J = 7.0 Hz, 2H), 1.35 (t, J = 7.0 Hz, 3H). ^{13}C NMR (125 MHz, $(CD_3)_2SO$): δ 165.2, 163.1, 153.8, 138.4, 137.2, 132.4, 129.7, 127.6, 61.2, 14.2. HRMS (ESI$^+$) *m/z* calcd. for $C_{12}H_{13}N_4O_2$ $[M+H]^+$ 245.1039, found 245.1045.

Ethyl 4-(1,2,4-triazin-5-yl)benzoate (S6): To a flame-dried Schlenk tube under N_2 was added a solution of **S5** (70.9 mg, 0.290 mmol) in anhydrous THF (10 mL). Isoamyl nitrite (0.39 mL, 2.9 mmol) was added, and the Schlenk tube was sealed and stirred at 65 ºC for 16 h. The yellow solution was diluted with EtOAc (20 mL) and was washed with H_2O (2 x 30 mL) and brine (1 x 30 mL). The aqueous layer was extracted with EtOAc (1 x 30 mL), and the combined organic layers were dried with $MgSO_4$, filtered, and concentrated *in vacuo*. The crude oil was dry-loaded on silica and purified by flash chromatography (eluting with 10% EtOAc in hexanes) to give **S6** (27 mg, 41%) as a yellow solid. ^{1}H NMR (400 MHz, $CDCl_3$): δ 9.77 (d, J = 2.0 Hz, 1H), 9.74 (d, J = 2.0 Hz, 1H), 8.27–8.22 (m, 4H), 4.43 (q, J = 7.1 Hz, 2H), 1.43 (t, J = 7.1 Hz, 3H). ^{13}C NMR (125 MHz, $CDCl_3$): δ 165.8, 157.7, 154.7, 147.0, 134.2, 130.7, 127.7, 61.7, 29.8, 14.4. HRMS (ESI$^+$) *m/z* calcd. for $C_{12}H_{12}N_3O_2$ $[M+H]^+$ 230.0930, found 230.0929.

4-(1,2,4-Triazin-5-yl)benzoic acid (S7): To a round-bottom flask was added **S6** (23.6 mg, 0.102 mmol) and EtOH (5 mL). A solution of sodium hydroxide (0.52 mL of a 1 M solution) was added, and the solution was stirred at room temperature under N_2 overnight. The reaction was acidified with 1 M HCl to pH 2, then diluted with H_2O (20 mL) and EtOAc (20 mL). The aqueous layer was extracted with EtOAc (2 x 20 mL), and the combined organic layers were dried with $MgSO_4$. The mixture was filtered and the solvent was evaporated *in vacuo*. The crude solid was dry-loaded on silica and purified by flash chromatography (eluting with 1–5% MeOH in CH_2Cl_2) to give **S7** (7.6 mg, 37%) as a beige solid. ^{1}H NMR (400 MHz, $(CD_3)_2SO$): δ 13.35 (br s, 1H), 10.18 (d, J = 1.9 Hz, 1H), 9.86 (d, J = 2.0 Hz, 1H), 8.44 (d, J = 8.4 Hz, 2H), 8.14 (d, J = 8.0 Hz, 2H). ^{13}C NMR (125 MHz, $(CD_3)_2SO$): δ 166.7, 157.3, 153.7, 147.6, 136.8, 130.0, 127.9. HRMS (ESI$^+$) *m/z* calcd. for $C_{10}H_6N_3O_2$ $[M-H]^-$ 200.0460, found 200.0455.

***N*-(2-(2,5-Dioxo-2,5-dihydro-1H-pyrrol-1-yl)ethyl)-4-(1,2,4-triazin-5-yl)benzamide (S9)**: To an oven-dried round-bottom flask under N_2 was added a solution of **S7** (5.8 mg, 0.0288 mmol) in dry DMF (0.5 mL). Diisopropylethylamine (0.03 mL, 0.2 mmol) and HCTU (15.4 mg, 0.0372 mmol) were added. After 2 min, **S8** (12.3 mg, 0.0484 mmol) in dry DMF (0.25 mL) was added dropwise, and the solution was stirred under N_2 overnight. The reaction was diluted with CH_2Cl_2 (20 mL), and the organic layer was washed with 1 M LiCl (4 x 20 mL). The combined organic layers were dried with $MgSO_4$, filtered, and concentrated *in vacuo*. The crude yellow solid was purified by flash

chromatography (eluting with 1% MeOH in CH_2Cl_2) to give **S9** (2.9 mg, 31%) as a yellow solid. ^{1}H NMR (600 MHz, $(CD_3)_2SO$): δ 10.17 (d, *J* = 1.8 Hz, 1H), 9.83 (d, *J* = 1.8 Hz, 1H), 8.78 (t, *J* = 5.9 Hz, 1H), 8.41 (d, *J* = 8.3 Hz, 2H), 7.94 (d, *J* = 8.3 Hz, 2H), 7.02 (s, 2H), 3.61 (t, *J* = 5.6 Hz, 2H), 3.44 (apparent q, *J* = 5.7 Hz, 2H). ^{13}C NMR (150 MHz, $(CD_3)_2SO$): δ 171.1, 165.7, 157.3, 153.8, 147.6, 137.8, 135.4, 134.6, 128.0, 127.7, 37.7, 37.1. HRMS (ESI$^+$) *m/z* calcd. for $C_{16}H_{13}N_5O_3Na$ $[M+Na]^+$ 346.0916, found 346.0911.

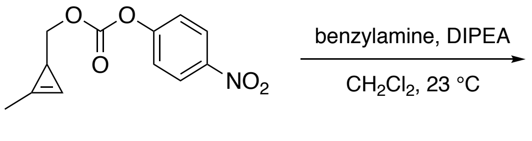

S10 **S11**

(2-Methylcycloprop-2-en-1-yl)methyl benzylcarbamate (S11):
To a flame-dried round-bottom flask was added **S10** (83 mg, 0.33 mmol) and anhydrous CH_2Cl_2 (2 mL). *N,N*-Diisopropylethylamine (DIPEA, 180 µL, 1.04 mmol) and benzylamine (110 µL, 1.04 mmol) were added, and the reaction was stirred at ambient temperature for 16 h. The mixture was diluted with CH_2Cl_2 (15 mL), then washed with saturated $NaHCO_3$ (3 x 20 mL) and brine (2 x 20 mL). The organic layer was dried with $MgSO_4$, filtered, and concentrated *in vacuo*. The crude oil was purified on silica (eluting with 10–25% EtOAc in hexanes), then further purified by HPLC (10–90% MeCN in H_2O over 15 min). The desired fractions were combined and lyophilized to give **S11** as a white solid (39 mg, 54%). ^{1}H NMR (500 MHz, $CDCl_3$) δ 7.35–7.27 (m, 5H), 6.56 (s, 1H), 4.96 (br s, 1H), 4.38 (d, *J* = 5.9 Hz, 2H), 4.01–3.98 (m, 1H), 3.97–3.93 (dd, *J* = 11.0, 5.2 Hz, 1H), 2.13 (s, 3H), 1.67–1.64 (m, 1H). ^{13}C NMR (150 MHz, $CDCl_3$) δ 157.0, 138.9, 128.8, 127.7, 127.6, 102.3, 72.6, 45.2, 17.4, 11.8. HRMS (ESI$^+$) *m/z* calculated for $C_{13}H_{15}NO_2Na$ $[M+Na]^+$ 240.1001, found 240.0995.

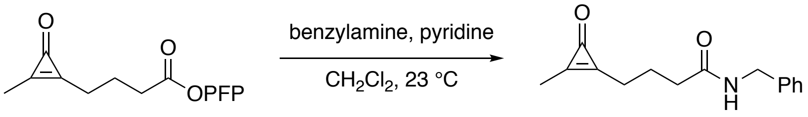

S12 **S13**

***N*-Benzyl-4-(2-methyl-3-oxocycloprop-1-en-1-yl)butanamide (S13):**
To a flame-dried round-bottom flask was added **S12** (63 mg, 0.20 mmol) and anhydrous CH_2Cl_2 (3 mL). Anhydrous pyridine (24 µL, 0.30 mmol) and benzylamine (11 µL, 0.10 mmol) were added, and the reaction was stirred at ambient temperature overnight. The resulting solution was diluted with H_2O (20 mL), acidified to pH ~ 1 with 1 M HCl, and extracted with CH_2Cl_2 (3 x 10 mL). The organic layers were combined, dried with $MgSO_4$, filtered, and concentrated *in vacuo*. The crude oil was purified on silica (eluting with 0–100% acetone in EtOAc), then further purified by HPLC (10–90% MeCN in H_2O over 15 min). The desired fractions were combined and lyophilized to give **S13** as a white solid (11 mg, 23%). ^{1}H NMR (600 MHz, $CDCl_3$) δ 7.32–7.29 (m, 2H), 7.26–7.23 (m, 3H), 6.74 (br s, 1H), 4.41 (d, *J* = 5.8 Hz, 2H), 2.61 (t, *J* = 6.9 Hz, 2H), 2.38 (t, *J* = 7.0 Hz, 2H), 2.24 (s, 3H), 2.02 (quint, *J* = 7.0 Hz, 2H). ^{13}C NMR (150 MHz, $CDCl_3$) δ 171.8,

160.9, 160.4, 156.9, 138.4, 128.8, 128.0, 127.6, 43.7, 35.0, 25.2, 22.1, 11.4. HRMS (ESI^+) *m/z* calculated for $C_{15}H_{17}NO_2Na$ $[M+Na]^+$ 266.1157, found 266.1149.

Preparation of triazine-labeled of NanoLuciferase (Nluc-Triazine)

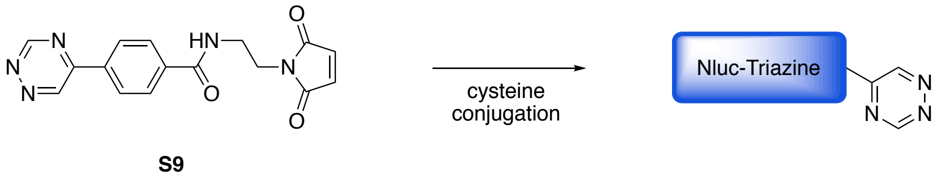

E. coli BL21(DE3) cells were transformed with pCold-His6-NLuc(G180C)[12] and used to inoculate 50 mL of LB broth containing ampicillin (40 μg/mL). The culture was incubated at 37 ºC with shaking (225 rpm) for 16 hr. A 15-mL aliquot of the starter culture was used to inoculate 1 L of LB broth containing ampicillin (40 μg/mL), and the resulting culture was incubated at 37 ºC with shaking (225 rpm). At OD_{600} = 0.6, the flask was cooled in an ice water bath for 20 min. Protein expression was induced by adding 1 M isopropyl β-D-1-thiogalactopyranoside (IPTG) to a final concentration of 1 mM, and the culture was incubated at 16 ºC with shaking (225 rpm) for 20 h.

Cells were collected via centrifugation (3500 x *g* for 10 min at 4 ºC). The pellet was resuspended in 30 mL of buffer (50 mM sodium phosphate, 20 mM imidazole, pH 7.8) and treated with Halt™ Protease Inhibitor Cocktail (Thermo Scientific). The cells were lysed via sonication, and the lysate was centrifuged (4500 x *g* for 45 min at 4 ºC). The lysate was passed through a 0.22 μM PES membrane syringe filter (Olympus), and the desired protein was purified by Ni^{2+}-affinity chromatography (eluting with 250 mM imidazole in 50 mM sodium phosphate buffer). Fractions containing the desired protein were combined and dialyzed against 50 mM sodium phosphate (pH 7.8) overnight. The final protein concentration was determined using a Jasco V-730 UV-Vis spectrophotometer and a predicted extinction coefficient of 24750 M^{-1} cm^{-1} (ProtParam).

Dithiothreitol (DTT) was added to the Nluc (G180C) sample from above (1 mM final concentration). The sample was diluted to a final protein concentration of 50 μM with phosphate buffered saline (PBS, pH 7.3), then treated with triazine **S9** (10 mM stock in DMSO, 1 mM final concentration). The sample was incubated at room temperature for 2 h, and aliquots were analyzed by mass spectrometry. Once complete conversion was observed, the sample was concentrated via spin filtration (3 kDa MW cutoff, Millipore) and washed with PBS (pH 7.3, 3 x 400 μL). After the final wash, the sample was concentrated to a final volume of 50 μL and stored at –20 ºC. Protein concentrations were determined as previously described.

Preparation of GFP-Cp

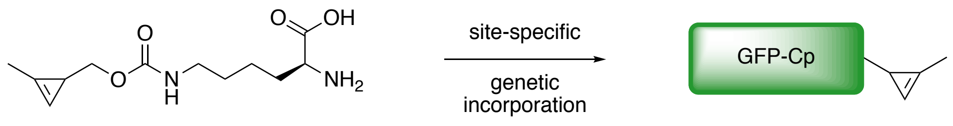

E. coli TOP10 cells transformed with pULTRA-WTPylRS/pBAD-GFP-150 were used to inoculate 3 mL of LB broth containing spectinomycin (50 µg/mL) and ampicillin (100 µg/mL). After 16 h, the starter culture (250 µL) was used to inoculate autoinduction expression media (AIM, 25 mL, Table S1) containing 1 mM **S14**, spectinomycin (50 µg/mL), and ampicillin (100 µg/mL).[13] The culture was incubated at 37 ºC with shaking (225 rpm) for 48 h.

Cells were collected via centrifugation (4500 rpm for 20 min at 4 ºC). The pellet was resuspended in 5 mL of PBS (pH 7.3), then treated with phenylmethylsulfonyl fluoride (PMSF, 500 µM final concentration) and a protease inhibitor cocktail (Sigma Aldrich). The cells were lysed via sonication and the lysate was centrifuged (14500 rpm for 30 min at 4 ºC). The lysate was passed through a 0.45 µM PES membrane syringe filter (Olympus) and was added to Profinity™ IMAC resin (BioRad, 400 µL bed volume). The slurry was gently rocked for 1 h at 4 ºC, then washed with wash buffer (20 mM imidazole in PBS, pH 7.3, 8 mL). **GFP-Cp** was eluted using 1 mL of elution buffer (250 mM imidazole in PBS, pH 7.3). The protein was concentrated via spin filtration (3 kDa MW cutoff, Millipore), washed with PBS (pH 7.3, 500 µL), then concentrated again. This was repeated three times to remove excess imidazole. On the final wash, the protein was concentrated to a final volume of 40 µL. The final protein concentration was determined by measuring the absorbance value at 488 nm using a Jasco V-730 UV-Vis spectrophotometer and an extinction coefficient of 88300 M^{-1} cm^{-1}.[14] Successful incorporation of amino acid **S12** was verified through mass spectrometry.

Table S1. Autoinduction expression media components.[a]

Aspartate (5%)[b]	2.5 mL
Glycerol (40%)[b]	0.625 mL
Glucose (40%)[b]	62.5 µL
L-Arabinose (20%)[b]	0.125 mL
Lactose (10%)[b]	0.1 mL
25X 18 Amino Acid Mix	2 mL
50X M-Salts	1 mL
1 M $MgSO_4$	0.1 mL
5000X Trace Metals	10 µL

[a]*Final volume of 50 mL, pH adjusted to 7.3*
[b]*Percentage weight by volume*

One-pot, dual labeling reaction on model proteins

Nluc-Triazine and **GFP-Cp** were mixed 1:1 in PBS (pH 7.3, 2 μM final concentration for each protein conjugate). The resulting mixture was treated with TMTH **9** (25 mM stock in DMSO, 1 mM final concentration) and tetrazine **3** (10 mM stock in DMSO, 1 mM final concentration). The solution was briefly vortexed and then incubated at room temperature for 3 h. Aliquots of the reaction solution (10 μL) were analyzed by mass spectrometry over time. Signal intensities of the individual cycloadducts were normalized to their respective unreacted starting materials.

One-pot, dual labeling reaction in cell lysate

An overnight culture of *E. coli* TOP10 cells (25 mL, LB Broth) was pelleted (3500 x *g* for 10 min at 4 ºC) and re-suspended in PBS (pH 7.3, 200 μL). The suspension was treated with PMSF (500 μM final concentration) and a protease cocktail inhibitor (Sigma Aldrich), then lysed via sonication. The lysate was clarified (14500 rpm for 30 min at 4 ºC) and the supernatant was collected. Total protein concentrations were determined using a Pierce™ BCA protein assay kit (Thermo Fisher).

Clarified bacterial cell lysate (30 μg) was treated with triazine **2** (10 mM stock in MeCN), cyclopropene **S11** (10 mM stock in MeCN), tetrazine **3** (10 mM stock in MeCN), and TMTH **9** (50 mM stock in MeCN). The mixtures were diluted with PBS (pH 7.3) to a final volume of 50 μL (1 mM final concentration for all bioorthogonal reagents), then allowed to stand at ambient temperature for 3 h. The reactions were diluted with 50% MeCN in H_2O (50 μL) and concentrated via spin filtration (14500 rpm for 30 min at 4 ºC) using a 3 kDa MW cutoff spin filter (Millipore) to a final volume of ~ 50 μL. The filtrates were analyzed by LC-MS.

One-pot, triple labeling reaction

Triazine **2** (10 mM stock in MeCN), cyclopropene **S11** (10 mM stock in MeCN), cyclopropeneone **S13**, TMTH **9** (50 mM stock in MeCN), tetrazine **S15** (10 mM stock in MeCN), and phosphine **S17** (10 mM stock in MeCN) were mixed at equimolar ratios, then diluted with MeCN to a final volume of 200 μL (1 mM final concentration for all reagents). The reaction was allowed to stand at ambient temperature for 18 h, then analyzed by LC-MS. Control reactions were conducted analogously using different reaction partners.

Computational studies

Calculations were performed with the Gaussian 09.[15] The geometry optimization of all the minima and transition states involved was carried out at the M06-2X level of theory[16] with the 6-31G(d) basis set.[17] The vibrational frequencies were computed at the same level to check whether each optimized structure is an energy minimum or a transition state and to evaluate its zero-point vibration energy (ZPVE) and thermal corrections at 298 K. A quasiharmonic correction was applied during the entropy calculation by setting all positive frequencies that are less than 100 cm^{-1} to 100 cm^{-1}.[18] The single-point energies and solvent effects in water were computed at the M06-2X/6-311+G(d,p) level using the gas-phase optimized structures at the M06-2X/6-31G(d) level. Solvation energies were evaluated by a self-consistent reaction field (SCRF) using the CPCM model,[19] where UFF radii were used.

Coordinates and energies of stationary points

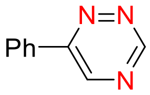

3N-6Ph

G(water) = -511.197341 Hartree

C	0.779183	0.013130	0.000499
C	3.386889	-0.068209	-0.033570
N	2.863080	1.127688	0.260984
N	2.714818	-1.189031	-0.270907
N	1.393616	-1.143256	-0.258470
C	-0.700656	0.013439	-0.003058
C	-1.392570	-1.183361	0.211540
C	-1.421563	1.190864	-0.224579
C	-2.781702	-1.195642	0.217865
H	-0.826008	-2.094434	0.371487
C	-2.812337	1.174522	-0.219253
H	-0.901160	2.121930	-0.429722
C	-3.495167	-0.017550	0.005618
H	-3.309776	-2.128116	0.390764
H	-3.361531	2.093117	-0.400063
H	-4.580655	-0.029630	0.010384
H	4.469062	-0.146334	-0.073090
C	1.544020	1.158636	0.286596
H	1.072887	2.100691	0.559044

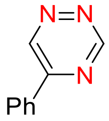

3N-5Ph

G(water) = -511.199246 Hartree

C	1.546637	1.159485	0.213857
C	2.729892	-1.096700	-0.188494
N	1.396695	-1.154084	-0.193928
N	3.487080	-0.018637	-0.018065
N	2.869680	1.136882	0.198351
H	3.266041	-2.028710	-0.345079
C	0.771315	0.003995	-0.001795
C	-0.708556	0.013588	-0.004471
C	-1.398701	-1.192228	0.160699
C	-1.431727	1.198673	-0.174982
C	-2.787502	-1.208237	0.170059
H	-0.829505	-2.107634	0.281696
C	-2.822029	1.178601	-0.170073
H	-0.916332	2.140065	-0.338067
C	-3.502456	-0.023326	0.006589
H	-3.313834	-2.147791	0.305509
H	-3.373338	2.102796	-0.310936
H	-4.587945	-0.037126	0.012239
H	1.099491	2.126168	0.421793

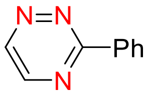

3N-3Ph

G(water) = -511.199776 Hartree

C	0.789293	0.009378	-0.000325
C	3.399297	-0.082668	0.000825
N	2.723215	-1.225679	-0.000509
N	1.404513	-1.179969	-0.001212
C	-0.692807	0.002772	-0.000284
C	-1.394086	-1.207474	0.000257
C	-1.395611	1.211252	-0.000534
C	-2.783717	-1.204225	0.000755
H	-0.838180	-2.138189	0.000371
C	-2.785595	1.208019	-0.000373
H	-0.841620	2.143451	-0.000672
C	-3.481981	0.001601	0.000351
H	-3.324001	-2.145739	0.001302
H	-3.326881	2.148997	-0.000498
H	-4.567819	0.000872	0.000619
H	4.481235	-0.160630	0.001491
C	2.734089	1.145245	0.000917
H	3.278010	2.087662	0.000109
N	1.415980	1.199958	-0.000029

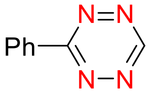

4N-3Ph

G(water) = -527.213293 Hartree

C	0.795954	-0.000001	-0.000312
C	3.344786	0.000000	0.000332
N	2.735472	-1.186514	0.000292
N	1.426931	-1.186701	-0.000008
N	2.735472	1.186514	-0.000108
N	1.426931	1.186700	-0.000451
C	-0.681194	-0.000001	-0.000145
C	-1.381274	1.210967	0.000099
C	-1.381274	-1.210966	-0.000212
C	-2.770752	1.206739	0.000257
H	-0.828010	2.143429	0.000139
C	-2.770754	-1.206738	-0.000063
H	-0.828012	-2.143429	-0.000401
C	-3.467186	0.000000	0.000177
H	-3.311732	2.147647	0.000449
H	-3.311730	-2.147649	-0.000139
H	-4.552928	0.000003	0.000302

H	4.428939	0.000000	0.000779

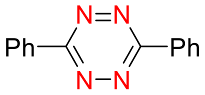

4N-36Ph

G(water) = -758.159878 Hartree

C	-0.000124	-1.287597	-0.000573
C	0.000124	1.287597	-0.000573
N	1.183187	0.651657	-0.001373
N	1.183061	-0.652015	-0.001280
N	-1.183061	0.652015	-0.001280
N	-1.183187	-0.651657	-0.001373
C	0.000214	2.764533	0.000244
C	1.210798	3.465467	0.001564
C	-1.210325	3.465451	-0.000733
C	1.206617	4.855025	0.001864
H	2.143368	2.912363	0.002220
C	-1.206155	4.855025	-0.000518
H	-2.142875	2.912278	-0.002082
C	0.000214	5.552030	0.000788
H	2.147731	5.395799	0.002879
H	-2.147320	5.395715	-0.001343
H	0.000236	6.637766	0.001079
C	-0.000214	-2.764533	0.000244
C	-1.210798	-3.465467	0.001564
C	1.210325	-3.465451	-0.000733
C	-1.206617	-4.855025	0.001864
H	-2.143368	-2.912363	0.002220
C	1.206155	-4.855025	-0.000518
H	2.142875	-2.912278	-0.002082
C	-0.000214	-5.552030	0.000788
H	-2.147731	-5.395799	0.002879
H	2.147320	-5.395715	-0.001343
H	-0.000236	-6.637766	0.001079

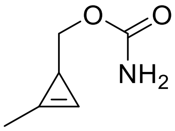

Cp

G(water) = -439.038604 Hartree

C	-2.166718	0.175532	0.058874
C	-2.006027	-0.469760	1.170833
C	-1.431480	-1.126459	-0.044027
H	-2.131475	-0.535413	2.240679
C	0.062086	-1.177135	-0.308638
H	0.539951	-2.010779	0.217916
H	0.269937	-1.302805	-1.377421
O	0.637711	0.050638	0.139259
C	1.974764	0.141012	-0.018915
O	2.668768	-0.713420	-0.519492
N	2.429227	1.320799	0.498171
H	3.369185	1.583352	0.247576
H	1.764153	2.052257	0.695037
H	-1.937570	-1.997520	-0.475794
C	-2.622171	1.345280	-0.722413
H	-3.287944	1.031457	-1.532692
H	-3.146818	2.067822	-0.092923
H	-1.758564	1.837471	-1.181998

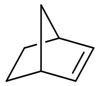

NB

G(water) = -272.545754 Hartree

C	-1.275344	-0.668267	-0.500391
C	-0.084771	-1.124602	0.325215
C	1.179937	-0.777633	-0.521120
C	1.179960	0.777613	-0.521143
C	-1.275321	0.668278	-0.500390
C	-0.084742	1.124606	0.325239
C	-0.030779	0.000005	1.376394
H	-1.915633	-1.325220	-1.079425
H	-0.114261	-2.151779	0.692510
H	2.080314	-1.172541	-0.039936
H	1.123410	-1.201886	-1.526509
H	1.123255	1.201826	-1.526539
H	2.080396	1.172565	-0.040118
H	-1.915564	1.325267	-1.079429
H	-0.114276	2.151779	0.692547
H	-0.897291	0.000013	2.042551
H	0.896013	-0.000019	1.961528

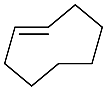

TCO

G(water) = -312.998444 Hartree

C	-0.413966	-0.522065	-1.358397
H	-1.490184	-0.336794	-1.334889
C	0.413966	0.522065	-1.358397
H	1.490184	0.336794	-1.334889
C	0.034981	-1.871510	-0.901221
H	-0.510280	-2.704358	-1.358222
H	1.102540	-2.008137	-1.112700
C	-0.034981	1.871510	-0.901221
H	-1.102540	2.008137	-1.112700
H	0.510280	2.704358	-1.358222

C	0.183001	1.877834	0.635533
H	1.260794	1.924503	0.841500
H	-0.248046	2.798155	1.048278
C	-0.413966	0.660993	1.376189
H	-0.560669	0.965017	2.418884
H	-1.420918	0.465721	0.984525
C	-0.183001	-1.877834	0.635533
H	0.248046	-2.798155	1.048278
H	-1.260794	-1.924503	0.841500
C	0.413966	-0.660993	1.376189
H	0.560669	-0.965017	2.418884
H	1.420918	-0.465721	0.984525

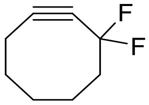

DIFO

G(water) = -510.299493 Hartree

C	-0.329082	1.579977	0.266345
C	-1.673885	1.181350	-0.409347
C	0.409933	-1.178338	-0.075735
C	-2.539058	0.122782	0.310012
C	-0.764313	-1.456068	-0.066028
C	-2.218621	-1.340393	-0.083054
C	0.972801	1.162080	-0.444633
C	1.505066	-0.202185	-0.016281
H	-1.487460	0.851540	-1.439914
H	-2.413455	0.229989	1.393485
H	-2.282352	2.087562	-0.493422
H	-3.598064	0.303551	0.095483
H	-2.704303	-2.044370	0.599352
H	-2.590949	-1.550943	-1.092420
H	1.775989	1.877226	-0.234915
H	0.833271	1.130813	-1.530264
H	-0.307370	2.670707	0.350928
H	-0.296885	1.202829	1.295757
F	2.563487	-0.544981	-0.797018
F	1.980350	-0.118811	1.260158

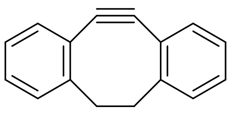

DIBO

G(water) = -616.569577 Hartree

C	1.898010	0.794625	0.022116
C	1.849374	-0.601117	0.270603
C	0.528818	-1.276479	0.579968
C	-0.528875	-1.276678	-0.579828
C	-1.849402	-0.601158	-0.270680
C	-1.897966	0.794599	-0.022145
C	-0.606901	1.406197	0.009197
C	0.606960	1.406243	-0.009110
C	-3.048984	-1.307798	-0.257496
C	-4.263124	-0.664239	-0.021388
C	-4.296781	0.708777	0.207263
C	-3.113867	1.439545	0.210245
C	3.113930	1.439526	-0.210290
C	4.296813	0.708705	-0.207339
C	4.263097	-0.664305	0.021342
C	3.048926	-1.307806	0.257474
H	0.087127	-0.785756	1.454801
H	0.733785	-2.310717	0.872057
H	-0.733880	-2.311024	-0.871515
H	-0.087185	-0.786309	-1.454852
H	-3.033843	-2.378707	-0.444115
H	-5.184913	-1.237794	-0.021407
H	-5.242920	1.209212	0.387266
H	-3.120850	2.508387	0.397337
H	3.120953	2.508372	-0.397358
H	5.242972	1.209100	-0.387347
H	5.184860	-1.237901	0.021380
H	3.033739	-2.378702	0.444158

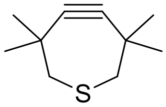

TMTH

G(water) = -788.531726 Hartree

C	0.604741	-1.159786	0.217348
C	-1.443927	0.832813	-0.737771
C	-0.604734	-1.159824	0.217424
C	-1.882213	-0.437360	0.049601
C	1.443851	0.832699	-0.738016
C	1.882167	-0.437289	0.049486
H	-2.266681	1.558063	-0.751823
H	-1.223527	0.556001	-1.774850
H	2.266478	1.558088	-0.752272
H	1.223231	0.555876	-1.775051
S	0.000017	1.764908	-0.091140
C	2.914035	-1.207291	-0.780446
H	3.791959	-0.579822	-0.969762
H	3.242057	-2.103095	-0.244926
H	2.493325	-1.517347	-1.741534
C	2.467967	-0.054196	1.412561
H	2.717322	-0.949901	1.988232
H	3.382383	0.533857	1.273415
H	1.749221	0.538709	1.984946
C	-2.468066	-0.054319	1.412556
H	-3.382402	0.533863	1.273453
H	-2.717568	-0.949992	1.988226

H	-1.749251	0.538451	1.985023
C	-2.913864	-1.207285	-0.780681
H	-3.242132	-2.103095	-0.245302
H	-3.791717	-0.579820	-0.970341
H	-2.492725	-1.517324	-1.741568

TS-Cp-3N-6Ph
G(water) = -950.193369 Hartree

C	0.394356	2.963677	0.259283
N	0.830392	2.536526	1.467336
C	1.790983	0.845760	0.129312
N	1.815633	1.690598	-0.955670
N	1.103297	2.755731	-0.898063
C	-1.096567	1.401715	-0.280304
C	-0.357867	0.280304	-0.235414
C	-1.273432	0.522197	0.916120
H	-0.056368	-0.544091	-0.865070
H	-0.835136	0.861498	1.860706
C	2.515768	-0.440316	0.007305
C	2.292948	-1.451136	0.947341
C	4.100378	-1.867707	-1.124248
C	2.978074	-2.657927	0.857740
H	1.561526	-1.297401	1.737747
C	3.886371	-2.867184	-0.177226
H	4.806442	-2.028750	-1.933079
H	2.797761	-3.437212	1.591582
H	4.421776	-3.808903	-0.248732
C	-2.521641	-0.307094	1.121565
H	-2.313588	-1.192030	1.732552
H	-3.305079	0.267341	1.627446
C	-1.956116	2.177822	-1.221824
H	-2.358601	3.081355	-0.756188
H	-1.388527	2.460290	-2.111672
H	-2.799574	1.546105	-1.519784
O	-2.990940	-0.723386	-0.162749
C	-4.102885	-1.494986	-0.132282
O	-4.688640	-1.806126	0.877941
N	-4.436817	-1.885409	-1.393969
H	-5.342865	-2.309923	-1.511721
H	-4.006260	-1.432864	-2.184786
C	3.418244	-0.658957	-1.037211
H	3.577099	0.127807	-1.766636
H	-0.264348	3.826455	0.245086
C	1.559781	1.452984	1.392819
H	1.984466	1.039377	2.305727

TS-Cp-3N-5Ph
G(water) = -950.195549 Hartree

C	-0.586383	2.298236	1.034854
N	-0.298607	3.007815	-0.086927
N	-0.582774	2.445514	-1.218274
C	-1.092335	1.185499	-1.161349
N	-1.585461	1.388619	1.117724
C	1.098958	0.741288	0.714881
C	0.698050	0.154984	-0.425141
C	1.890653	1.050793	-0.510070
H	0.392574	-0.818037	-0.783659
H	1.749144	2.025816	-0.985058
C	3.286215	0.502324	-0.709442
H	3.531286	0.420109	-1.773608
H	4.038808	1.148343	-0.245119
C	1.335536	0.441761	2.155858
H	1.671538	1.324008	2.706846
H	0.424342	0.057106	2.622043
H	2.118789	-0.320689	2.228376
O	3.342063	-0.796605	-0.112940
C	4.546528	-1.406476	-0.204569
O	5.522157	-0.916340	-0.721992
N	4.482755	-2.655079	0.340320
H	5.362471	-3.120069	0.499019
H	3.696731	-2.899695	0.921753
H	-1.215860	0.701605	-2.124336
H	-0.241933	2.742754	1.963918
C	-1.878042	0.815468	-0.026314
C	-2.930671	-0.220502	-0.084344
C	-3.340365	-0.844201	1.100114
C	-3.522538	-0.596832	-1.294452
C	-4.314531	-1.833026	1.070673
H	-2.879681	-0.538762	2.033812
C	-4.497470	-1.588803	-1.320978
H	-3.244843	-0.097042	-2.217480
C	-4.893029	-2.210832	-0.140287
H	-4.623292	-2.313792	1.993827
H	-4.954792	-1.869141	-2.264685
H	-5.653890	-2.985131	-0.162697

TS-Cp-3N-3Ph
G(water) = -950.196203 Hartree

C	1.780978	1.005181	-0.047291
N	1.462376	1.566251	-1.251395
N	0.708820	2.612981	-1.258310
C	0.235189	3.032858	-0.046585
C	-0.469068	0.262913	0.332060
C	-1.116281	1.444130	0.350317
C	-1.311415	0.575262	-0.853279
H	-1.752530	1.993850	1.029788
H	-0.819032	0.897086	-1.775685
C	2.528110	-0.272585	-0.087516

C	2.526273	-1.053611	-1.248192
C	3.869583	-1.945923	1.024574
C	3.194388	-2.272239	-1.267104
H	1.999929	-0.692477	-2.125703
C	3.866877	-2.722305	-0.131872
H	4.395323	-2.290018	1.910082
H	3.190808	-2.873833	-2.170846
H	4.387637	-3.674922	-0.150036
C	-2.614422	-0.148750	-1.109777
H	-3.296275	0.464146	-1.708120
H	-2.449534	-1.086980	-1.650349
C	-0.169946	-0.900576	1.208910
H	0.401337	-1.671603	0.686450
H	0.392056	-0.584929	2.092726
H	-1.122894	-1.334823	1.532159
O	-3.221699	-0.430506	0.154277
C	-4.422382	-1.049625	0.075567
O	-4.961453	-1.363749	-0.958982
N	-4.940524	-1.230517	1.324584
H	-5.731350	-1.851381	1.393024
H	-4.335446	-1.136563	2.125232
C	3.202194	-0.725996	1.049027
H	3.198993	-0.109618	1.941657
H	-0.432498	3.887063	-0.087065
N	1.831280	1.744057	1.100247
C	1.059193	2.791376	1.089780
H	1.049414	3.448986	1.956730

TS-Cp-4N-3Ph

G(water) = -966.219637 Hartree

C	-1.785023	1.002564	0.065584
N	-1.499250	1.599124	1.253867
N	-0.755036	2.653157	1.217687
C	-0.317368	3.018589	-0.016008
N	-1.075656	2.783947	-1.131608
N	-1.818109	1.739234	-1.087522
C	0.488480	0.264690	-0.381988
C	1.117914	1.446633	-0.379553
C	1.295785	0.581859	0.830007
H	1.723713	2.047359	-1.043074
H	0.778461	0.886927	1.744089
C	-2.516616	-0.279705	0.094626
C	-2.515449	-1.054113	1.259192
C	-3.825457	-1.971959	-1.023342
C	-3.167220	-2.281677	1.276707
H	-2.003938	-0.682638	2.141414
C	-3.822644	-2.743702	0.136696
H	-4.338732	-2.326589	-1.911781
H	-3.164317	-2.879842	2.182545
H	-4.330863	-3.703017	0.153526
C	2.598546	-0.135144	1.108721
H	3.266996	0.482748	1.716903
H	2.429443	-1.072951	1.648662
C	0.175577	-0.897472	-1.253360
H	-0.415957	-1.654083	-0.733022
H	-0.361488	-0.579017	-2.151016
H	1.128538	-1.348610	-1.552765
O	3.223891	-0.414042	-0.145919
C	4.426542	-1.030211	-0.049049
O	4.944131	-1.351051	0.994274
N	4.967337	-1.200207	-1.287750
H	5.772273	-1.802837	-1.350644
H	4.390050	-1.076008	-2.104252
C	-3.173886	-0.743971	-1.048182
H	-3.170978	-0.133000	-1.944473
H	0.342527	3.877216	-0.055999

TS-Cp-4N-36Ph

G(water) = -1197.164449 Hartree

C	1.432217	-1.474206	0.097185
N	0.815507	-1.647677	1.303093
N	-0.461007	-1.782980	1.290514
C	-1.071942	-1.712540	0.066793
N	-0.391417	-2.162910	-1.044175
N	0.878689	-2.032212	-1.028289
C	0.579943	0.639920	-0.374769
C	-0.737377	0.380842	-0.339618
C	-0.161762	1.104209	0.835124
H	-1.603099	0.452888	-0.982485
H	-0.045311	0.544295	1.767446
C	-2.553926	-1.780497	0.041321
C	-3.280780	-1.452248	1.188502
C	-4.615758	-2.155337	-1.153754
C	-4.670339	-1.481104	1.161054
H	-2.746388	-1.183961	2.094009
C	-5.340682	-1.832485	-0.008873
H	-5.135018	-2.431987	-2.066130
H	-5.231814	-1.227796	2.054877
H	-6.426001	-1.853884	-0.028273
C	2.889568	-1.228903	0.106740
C	3.487936	-0.672495	1.240983
C	5.027551	-1.231362	-1.013074
C	4.850420	-0.397125	1.243101
H	2.875952	-0.461458	2.112274
C	5.623086	-0.675292	0.116764
H	5.626736	-1.454999	-1.890277
H	5.310858	0.035938	2.125655
H	6.687023	-0.459108	0.120634
C	-0.437146	2.572561	1.071784
H	-1.324821	2.709435	1.698287

H	0.403845	3.060888	1.575352
C	1.629373	1.153040	-1.297120
H	2.609088	1.205381	-0.817178
H	1.701416	0.526445	-2.190501
H	1.334958	2.164688	-1.597197
O	-0.657032	3.191165	-0.197700
C	-0.928950	4.516851	-0.136678
O	-0.961963	5.158076	0.886922
N	-1.182890	5.000645	-1.383660
H	-1.233038	6.001776	-1.482435
H	-0.966271	4.440263	-2.192501
C	-3.225247	-2.128019	-1.133442
H	-2.649991	-2.384100	-2.016681
C	3.664226	-1.506171	-1.022845
H	3.189204	-1.940518	-1.896100

TS-NB-3N-6Ph

G(water) = -783.696583 Hartree

N	1.166999	1.877319	1.317935
N	0.003900	1.337572	1.290625
C	-0.584994	1.136918	0.066953
N	0.922380	2.663078	-0.917169
C	1.712704	2.204533	0.102306
C	0.885437	-0.359166	-0.749060
C	2.129252	0.259500	-0.652840
C	0.928227	-1.580548	0.150686
C	1.860181	-2.569071	-0.615770
C	2.951613	-0.594370	0.305762
C	3.257236	-1.889056	-0.505675
C	1.880831	-1.130861	1.271359
H	0.292385	-0.367295	-1.657900
H	2.598902	0.739804	-1.506495
H	-0.040730	-1.992263	0.440707
H	1.540863	-2.719633	-1.650920
H	1.854534	-3.544824	-0.120105
H	3.826418	-0.113358	0.747226
H	3.701188	-1.670650	-1.481148
H	3.960621	-2.519353	0.047180
H	2.251052	-1.982998	1.851231
H	1.468423	-0.388140	1.949654
C	-1.849145	0.365479	0.042528
C	-2.489021	0.099210	-1.172500
C	-2.399414	-0.138866	1.226072
C	-3.667774	-0.638651	-1.204302
H	-2.060119	0.461830	-2.103774
C	-3.575055	-0.880342	1.190158
H	-1.897013	0.067145	2.164909
C	-4.215043	-1.130245	-0.022178
H	-4.154695	-0.834382	-2.154696
H	-3.995188	-1.263743	2.115010
H	-5.132561	-1.709881	-0.045867
H	2.726592	2.589544	0.149394
C	-0.259571	2.121802	-0.920215
H	-0.986426	2.444823	-1.663839

TS-NB-3N-5Ph

G(water) = -783.697296 Hartree

N	0.719702	1.711838	-1.340477
N	1.154577	2.326112	-0.295564
C	0.691247	1.870151	0.908678
N	-0.569390	1.377119	1.055669
C	-0.130470	0.665654	-1.127533
C	1.737621	-0.055154	0.964599
C	1.240737	-0.742906	-0.138730
C	3.240188	0.066086	0.756761
C	3.771098	-1.382376	0.977883
C	2.434984	-1.042647	-1.033250
C	3.216693	-2.148254	-0.259951
C	3.349937	0.165861	-0.773640
H	1.319390	-0.175614	1.959248
H	0.414896	-1.445409	-0.069427
H	3.746717	0.833474	1.344769
H	3.429336	-1.805407	1.926728
H	4.865504	-1.384366	0.986392
H	2.208810	-1.286688	-2.072854
H	2.577841	-2.992837	0.013353
H	4.029977	-2.533135	-0.882980
H	4.372182	-0.018989	-1.120239
H	2.990362	1.103539	-1.191099
C	-1.019333	0.763638	-0.009738
H	-0.388279	0.096923	-2.014976
H	1.142792	2.337279	1.778441
C	-2.362320	0.144849	0.003270
C	-3.025727	-0.186504	-1.182495
C	-2.981655	-0.121646	1.230253
C	-4.281350	-0.784184	-1.142246
H	-2.575801	0.046500	-2.142860
C	-4.235171	-0.717701	1.267380
H	-2.460171	0.146771	2.143087
C	-4.887137	-1.053813	0.081583
H	-4.790464	-1.030786	-2.068666
H	-4.706262	-0.922875	2.223782
H	-5.867114	-1.520183	0.112962

TS-NB-3N-3Ph

G(water) = -783.697699 Hartree

N	1.171632	1.831161	1.410633
N	0.014688	1.279233	1.363855

C	-0.582092	1.151095	0.131085
N	-0.382794	2.112679	-0.828139
C	1.718495	2.211622	0.212435
C	0.840714	-0.321972	-0.748497
C	2.087026	0.298866	-0.671790
C	0.933719	-1.591049	0.079020
C	1.852749	-2.526032	-0.765519
C	2.947906	-0.586244	0.222406
C	3.243880	-1.833921	-0.662932
C	1.913007	-1.183381	1.191860
H	0.212485	-0.277593	-1.632785
H	2.537107	0.799228	-1.524215
H	-0.018648	-2.034521	0.375957
H	1.502355	-2.625432	-1.796562
H	1.875052	-3.526554	-0.322460
H	3.830463	-0.115151	0.659332
H	3.655814	-1.559916	-1.638610
H	3.972186	-2.480882	-0.164294
H	2.312754	-2.054851	1.721049
H	1.510100	-0.477372	1.914533
C	-1.840869	0.365203	0.082448
C	-2.660767	0.421003	-1.047579
C	-2.191821	-0.467500	1.149479
C	-3.818379	-0.347761	-1.108365
H	-2.384265	1.077947	-1.865951
C	-3.350758	-1.233116	1.083939
H	-1.552483	-0.496430	2.025984
C	-4.166274	-1.177062	-0.044574
H	-4.452158	-0.297579	-1.988627
H	-3.619965	-1.873726	1.918184
H	-5.070257	-1.776514	-0.093494
H	2.721541	2.621048	0.277416
C	0.790555	2.667271	-0.771674
H	1.040692	3.454663	-1.480354

TS-NB-4N-3Ph

G(water) = -799.723669 Hartree

N	1.142263	1.919608	1.357410
N	-0.013107	1.355546	1.351933
C	-0.587018	1.148580	0.129651
N	-0.356460	2.073407	-0.864681
N	0.792986	2.632801	-0.865547
C	1.648111	2.225818	0.130772
C	0.857358	-0.350557	-0.764744
C	2.088527	0.283851	-0.700373
C	0.948097	-1.582284	0.115451
C	1.882713	-2.540851	-0.686880
C	2.953581	-0.553547	0.232113
C	3.266560	-1.833467	-0.601713
C	1.916913	-1.122017	1.217360
H	0.211892	-0.321464	-1.636914
H	2.520957	0.806585	-1.548608
H	-0.003390	-2.021019	0.421405
H	1.540540	-2.684426	-1.715336
H	1.908690	-3.522249	-0.203607
H	3.828409	-0.056739	0.655118
H	3.683602	-1.594818	-1.584100
H	3.996111	-2.452780	-0.071271
H	2.319585	-1.968255	1.783499
H	1.503226	-0.392867	1.910797
C	-1.839324	0.361439	0.076105
C	-2.622079	0.357630	-1.081906
C	-2.219558	-0.416318	1.173834
C	-3.776806	-0.415336	-1.136746
H	-2.323094	0.971543	-1.925532
C	-3.374506	-1.188224	1.111804
H	-1.607202	-0.399895	2.069855
C	-4.155122	-1.190320	-0.042418
H	-4.384434	-0.410838	-2.036354
H	-3.667253	-1.787974	1.968023
H	-5.056503	-1.793755	-0.088086
H	2.644412	2.653050	0.113445

TS-NB-4N-36Ph

G(water) = -1030.670515 Hartree

N	-0.639573	-0.891865	1.372192
N	0.639588	-0.891858	1.372192
C	1.251977	-0.998139	0.150136
N	0.636564	-1.788453	-0.803770
N	-0.636555	-1.788463	-0.803770
C	-1.251969	-0.998160	0.150142
C	0.695529	0.883175	-0.790055
C	-0.695470	0.883182	-0.790203
C	1.129063	2.071626	0.051711
C	0.778386	3.301384	-0.840878
C	-1.129179	2.071679	0.051398
C	-0.778194	3.301420	-0.841094
C	-0.000197	2.164261	1.092744
H	1.285133	0.562607	-1.643797
H	-1.284903	0.562549	-1.644050
H	2.158334	2.047395	0.414296
H	1.204260	3.216508	-1.844382
H	1.175209	4.214205	-0.386608
H	-2.158548	2.047507	0.413701
H	-1.203795	3.216569	-1.844715
H	-1.175097	4.214256	-0.386926
H	-0.000247	3.131134	1.606633
H	-0.000323	1.366602	1.832602
C	2.731266	-0.902414	0.117738
C	3.438040	-1.300128	-1.020110

C	3.420342	-0.375166	1.213429
C	4.822416	-1.173176	-1.057756
H	2.895468	-1.719786	-1.861380
C	4.804800	-0.249901	1.169135
H	2.861203	-0.079389	2.095421
C	5.508759	-0.647456	0.034607
H	5.367718	-1.488116	-1.942156
H	5.335390	0.157009	2.024547
H	6.589487	-0.548683	0.002147
C	-2.731231	-0.902454	0.117776
C	-3.438009	-1.300044	-1.020120
C	-3.420385	-0.375355	1.213499
C	-4.822378	-1.173008	-1.057820
H	-2.895445	-1.719579	-1.861456
C	-4.804835	-0.250062	1.169185
H	-2.861276	-0.079689	2.095550
C	-5.508759	-0.647427	0.034577
H	-5.367644	-1.487786	-1.942300
H	-5.335442	0.156746	2.024635
H	-6.589481	-0.548605	0.002078

TS-TCO-3N-6Ph

G(water) = -824.161177 Hartree

C	-1.004112	1.196477	-0.033370
C	1.108634	2.548884	-0.356698
C	1.888700	0.584231	-0.230986
H	2.293339	0.623240	-1.243544
C	0.787732	-0.233873	-0.046927
H	0.545899	-0.521130	0.977526
N	0.563978	2.702192	0.883637
N	0.323478	2.353856	-1.470875
N	-0.743954	1.667165	-1.298483
C	0.409228	-1.227876	-1.103421
H	-0.659754	-1.467032	-1.093211
H	0.649956	-0.814913	-2.090764
C	2.907268	0.704102	0.872285
H	3.463545	1.647422	0.826444
H	2.393532	0.680037	1.841313
C	1.219561	-2.521329	-0.863175
H	1.024334	-3.202316	-1.699960
H	0.830786	-3.020081	0.034723
C	3.893797	-0.477455	0.771737
H	4.559485	-0.439243	1.642191
H	4.534117	-0.333229	-0.108875
C	3.247995	-1.873052	0.690115
H	3.999815	-2.594422	1.028406
H	2.433752	-1.939215	1.424619
C	2.739443	-2.330900	-0.707563
H	3.210456	-3.292118	-0.939937
H	3.097700	-1.637250	-1.479952
C	-2.156963	0.285410	0.131843
C	-3.037336	0.039003	-0.927564
C	-2.358996	-0.367312	1.352473
C	-4.104952	-0.835384	-0.758304
H	-2.870492	0.542631	-1.873575
C	-3.429135	-1.239671	1.518979
H	-1.664743	-0.202934	2.173331
C	-4.305921	-1.475529	0.463185
H	-4.785767	-1.017209	-1.584332
H	-3.573823	-1.740124	2.471415
H	-5.139458	-2.159387	0.589974
H	2.043233	3.067925	-0.548709
C	-0.525669	2.005163	1.039186
H	-1.066059	2.071536	1.982197

TS-TCO-3N-5Ph

G(water) = -824.161517 Hartree

C	0.508000	1.486432	0.737534
C	-0.295170	1.745654	-1.617094
C	-1.750578	0.358563	-0.705965
H	-2.564351	1.077765	-0.807196
C	-1.265276	0.146833	0.569546
H	-0.648036	-0.741639	0.719079
N	0.829118	0.996191	-1.523156
N	-0.573437	2.780479	-0.762489
N	-0.150546	2.646302	0.445539
C	-2.076225	0.577695	1.760012
H	-1.458505	0.777010	2.643011
H	-2.605815	1.506819	1.517650
C	-1.747164	-0.769786	-1.699674
H	-1.712914	-0.418067	-2.737230
H	-0.856055	-1.389693	-1.537231
C	-3.093108	-0.536006	2.093360
H	-3.756895	-0.164050	2.882791
H	-2.556542	-1.392598	2.523164
C	-3.020491	-1.618234	-1.491721
H	-2.951894	-2.501682	-2.137609
H	-3.890158	-1.048166	-1.844870
C	-3.277536	-2.067764	-0.041691
H	-3.926565	-2.948951	-0.085721
H	-2.335012	-2.422612	0.396305
C	-3.941292	-1.027185	0.905820
H	-4.849498	-1.476914	1.321298
H	-4.284246	-0.159679	0.326527
H	-0.754594	1.827689	-2.597849
H	0.742660	1.343902	1.787395
C	1.272656	0.869027	-0.293865
C	2.474477	0.051022	-0.023890
C	2.903474	-0.875675	-0.981465
C	3.190769	0.180157	1.170125

C	4.021619	-1.663785	-0.741531
H	2.344980	-0.961231	-1.908069
C	4.308850	-0.612236	1.408767
H	2.893508	0.921140	1.906187
C	4.725102	-1.537190	0.455519
H	4.346315	-2.381361	-1.488863
H	4.860494	-0.499159	2.336817
H	5.598522	-2.154462	0.642437

TS-TCO-3N-3Ph

G(water) = -824.161357 Hartree

C	-0.982494	1.208852	-0.070956
C	1.129373	2.589377	-0.281281
C	1.878246	0.589519	-0.199110
H	2.318599	0.648840	-1.196382
C	0.761264	-0.222298	-0.071376
H	0.490994	-0.535682	0.937606
N	0.517084	2.584704	0.949671
N	-0.538947	1.869457	1.057373
N	-0.798162	1.746621	-1.314094
C	0.419094	-1.189773	-1.165491
H	-0.651229	-1.418686	-1.200988
H	0.700785	-0.757021	-2.134181
C	2.867022	0.666907	0.935700
H	3.436124	1.603557	0.931576
H	2.324462	0.623644	1.887458
C	1.206403	-2.497578	-0.931964
H	1.029955	-3.156816	-1.790284
H	0.783643	-3.012279	-0.059017
C	3.844634	-0.521398	0.831280
H	4.485795	-0.509429	1.720524
H	4.511076	-0.361664	-0.027273
C	3.190512	-1.908890	0.698014
H	3.925968	-2.644301	1.041799
H	2.353490	-1.984015	1.404994
C	2.721935	-2.328207	-0.724863
H	3.190576	-3.288579	-0.965576
H	3.111801	-1.620435	-1.469063
C	-2.127821	0.286661	0.127202
C	-2.932555	-0.082045	-0.954449
C	-2.393420	-0.240233	1.394726
C	-3.990400	-0.966341	-0.767882
H	-2.720976	0.338344	-1.932148
C	-3.451217	-1.123544	1.575987
H	-1.772358	0.061970	2.231980
C	-4.252175	-1.489856	0.495759
H	-4.613355	-1.244804	-1.612437
H	-3.652272	-1.526800	2.563863
H	-5.076981	-2.181234	0.639285
H	2.055542	3.153490	-0.329107
C	0.278408	2.471032	-1.413265
H	0.507671	2.953312	-2.361472

TS-TCO-4N-3Ph

G(water) = -840.187486 Hartree

C	-1.012127	1.217003	-0.071701
C	1.047417	2.567439	-0.371057
C	1.880567	0.575300	-0.219291
H	2.301894	0.644720	-1.224137
C	0.787513	-0.247208	-0.067038
H	0.499175	-0.515263	0.950951
N	0.469568	2.641470	0.863788
N	-0.589427	1.933004	1.017422
N	0.277100	2.409408	-1.487025
N	-0.784494	1.704997	-1.329095
C	0.441437	-1.242532	-1.131181
H	-0.624575	-1.491756	-1.144768
H	0.707005	-0.834113	-2.114236
C	2.864215	0.727700	0.908849
H	3.406566	1.679197	0.869204
H	2.327453	0.698150	1.864578
C	1.260192	-2.525979	-0.858926
H	1.094223	-3.215599	-1.694761
H	0.853951	-3.021074	0.032823
C	3.870637	-0.440944	0.833545
H	4.513833	-0.391042	1.719825
H	4.529660	-0.289653	-0.031835
C	3.242632	-1.844236	0.740987
H	3.993536	-2.555743	1.100807
H	2.411428	-1.915586	1.455529
C	2.773122	-2.313278	-0.667130
H	3.263218	-3.268048	-0.885587
H	3.139397	-1.617001	-1.433589
C	-2.143947	0.285067	0.121294
C	-2.909736	-0.135884	-0.970243
C	-2.436058	-0.196976	1.400312
C	-3.958825	-1.028911	-0.779003
H	-2.677083	0.249475	-1.957508
C	-3.484752	-1.090684	1.584596
H	-1.843294	0.144976	2.243113
C	-4.248079	-1.508801	0.496289
H	-4.553973	-1.347923	-1.629055
H	-3.708217	-1.460641	2.580480
H	-5.066011	-2.207653	0.642263
H	1.977325	3.109964	-0.501540

TS-TCO-4N-36Ph

G(water) = -1071.132935 Hartree

C	1.251570	-1.207255	0.038913
C	-1.251569	-1.207255	-0.038913
C	-0.691700	0.911665	0.021258
H	-1.212607	1.060429	-0.926092
C	0.691700	0.911665	-0.021258
H	1.212607	1.060430	0.926092
N	-0.677570	-1.552440	1.161720
N	0.597792	-1.546184	1.203521
N	-0.597791	-1.546184	-1.203522
N	0.677571	-1.552440	-1.161721
C	1.389688	1.452693	-1.235851
H	2.385383	1.018529	-1.372584
H	0.795922	1.219764	-2.128433
C	-1.389688	1.452692	1.235852
H	-2.385382	1.018527	1.372585
H	-0.795922	1.219764	2.128434
C	1.523803	2.983218	-1.080635
H	1.937706	3.379117	-2.015359
H	2.268251	3.195800	-0.302008
C	-1.523804	2.983217	1.080635
H	-1.937707	3.379116	2.015359
H	-2.268252	3.195799	0.302008
C	-0.222491	3.734131	0.745309
H	-0.363644	4.775166	1.055325
H	0.590070	3.351506	1.377787
C	0.222489	3.734131	-0.745309
H	0.363642	4.775166	-1.055325
H	-0.590071	3.351505	-1.377786
C	-2.730866	-1.143998	-0.102703
C	-3.502828	-1.327147	1.048155
C	-3.359348	-0.881299	-1.323029
C	-4.889929	-1.248692	0.973981
H	-3.005020	-1.539237	1.988652
C	-4.745553	-0.802816	-1.390782
H	-2.752343	-0.756691	-2.214609
C	-5.514051	-0.985376	-0.242963
H	-5.484451	-1.397670	1.870187
H	-5.227748	-0.602628	-2.342627
H	-6.596711	-0.924460	-0.297643
C	2.730867	-1.143998	0.102702
C	3.502828	-1.327146	-1.048155
C	3.359348	-0.881299	1.323029
C	4.889929	-1.248691	-0.973981
H	3.005020	-1.539236	-1.988652
C	4.745553	-0.802817	1.390783
H	2.752343	-0.756691	2.214609
C	5.514052	-0.985375	0.242963
H	5.484451	-1.397668	-1.870187
H	5.227748	-0.602629	2.342627
H	6.596711	-0.924460	0.297643

TS-DIFO-3N-6Ph

G(water) = -1021.45531 Hartree

C	-1.985725	3.023623	0.169973
C	-3.185097	2.166476	-0.298658
C	-0.453291	0.403881	0.079063
C	-3.561191	0.954542	0.564929
C	-1.456284	-0.324380	0.071055
C	-2.937585	-0.358037	0.134424
C	-0.639421	2.795611	-0.534549
C	0.255008	1.691637	0.052620
H	-3.032791	1.835809	-1.333736
H	-3.291244	1.116972	1.613542
H	-4.064458	2.816938	-0.323855
H	-4.641905	0.779540	0.529968
H	-0.056100	3.722825	-0.500137
H	-0.815633	2.576800	-1.594207
H	1.174581	1.619006	-0.537886
H	0.563879	1.952331	1.073455
H	-2.253845	4.072485	0.005403
H	-1.854735	2.919023	1.256037
F	-3.420812	-0.717036	-1.090379
F	-3.352089	-1.329739	0.998728
C	1.285945	-1.187948	-0.030484
C	-0.979930	-2.307114	-0.165323
N	0.862935	-1.616103	-1.268352
N	-0.273868	-2.193237	-1.348013
N	-0.336472	-2.541323	1.016706
H	-1.973261	-2.728171	-0.274273
C	2.544476	-0.415771	0.023988
C	2.975274	0.141066	1.233508
C	3.305196	-0.209486	-1.132080
C	4.155372	0.874598	1.291880
H	2.372253	0.017650	2.130120
C	4.483123	0.527214	-1.070291
H	2.961483	-0.639090	-2.066699
C	4.913463	1.067964	0.139689
H	4.479795	1.300559	2.236300
H	5.069391	0.678080	-1.971594
H	5.833308	1.642815	0.183341
C	0.826228	-1.961162	1.076187
H	1.443858	-2.104232	1.961099

TS-DIFO-3N-5Ph

G(water) = -1021.456171 Hartree

C	2.883119	2.549945	-0.513382
C	3.787439	1.305806	-0.699846
C	0.924971	0.623384	0.654872
C	3.227016	0.172799	-1.569294
C	1.415645	-0.355362	0.078419
C	2.472151	-0.898624	-0.803345

C	2.233497	2.727113	0.868669
C	0.868778	2.043702	1.048818
H	4.075091	0.895176	0.276019
H	2.549562	0.563622	-2.335472
H	4.720540	1.634385	-1.166999
H	4.034145	-0.357770	-2.085670
H	2.085920	3.794284	1.069066
H	2.917194	2.356026	1.640880
H	0.549422	2.141674	2.091611
H	0.105679	2.542034	0.435436
H	3.497399	3.437236	-0.697029
H	2.103573	2.565458	-1.287373
F	3.345402	-1.623517	-0.047304
F	1.942521	-1.773457	-1.708837
C	-0.876933	-0.197251	1.689255
C	0.237048	-2.110679	0.531648
N	-0.176878	-1.061484	2.484827
N	0.403284	-2.043259	1.896508
N	-0.911309	-1.721429	-0.075832
H	0.831289	-2.868944	0.034158
C	-1.513069	-0.725785	0.529534
H	-1.251438	0.688870	2.191869
C	-2.757701	-0.161311	-0.033142
C	-3.063521	-0.393448	-1.378981
C	-3.632936	0.602154	0.745785
C	-4.218877	0.139526	-1.935868
H	-2.379514	-0.992622	-1.970906
C	-4.788800	1.135674	0.184704
H	-3.429911	0.758154	1.801247
C	-5.082679	0.908308	-1.156980
H	-4.446486	-0.043070	-2.981550
H	-5.464755	1.721565	0.799650
H	-5.985574	1.324180	-1.593389

TS-DIFO-3N-3Ph

G(water) = -1021.456241 Hartree

C	1.937716	3.050177	-0.164686
C	3.101198	2.180828	0.366346
C	0.420797	0.395090	-0.167643
C	3.545633	1.004553	-0.513597
C	1.437718	-0.316507	-0.153115
C	2.919823	-0.329812	-0.163012
C	0.545928	2.804570	0.434584
C	-0.286426	1.684446	-0.210527
H	2.868819	1.809749	1.372420
H	3.328188	1.197473	-1.569062
H	3.972544	2.831519	0.486801
H	4.625616	0.843282	-0.427673
H	-0.045395	3.723403	0.347906
H	0.642951	2.597729	1.507198
H	-1.255613	1.622380	0.294389
H	-0.502004	1.924296	-1.260205
H	2.190509	4.095232	0.043174
H	1.883515	2.971123	-1.259190
F	3.355375	-0.733875	1.071178
F	3.389336	-1.262748	-1.042158
C	-1.260314	-1.233825	0.107579
C	1.030272	-2.310811	0.094895
N	-0.844207	-1.650876	1.338459
N	0.257813	-2.484293	-1.035046
N	-0.883434	-1.910199	-1.034055
H	2.015704	-2.758085	0.043761
C	-2.519910	-0.461021	0.019694
C	-3.046379	-0.114966	-1.229023
C	-3.164833	-0.035628	1.184349
C	-4.207695	0.644589	-1.308131
H	-2.537803	-0.452955	-2.126047
C	-4.326457	0.725565	1.099087
H	-2.747477	-0.315140	2.146011
C	-4.849867	1.067737	-0.145508
H	-4.614005	0.907163	-2.280169
H	-4.826094	1.049344	2.007047
H	-5.756409	1.661944	-0.209366
C	0.323407	-2.224391	1.328418
H	0.739689	-2.604844	2.258053

TS-DIFO-4N-3Ph

G(water) = -1037.479955 Hartree

C	-1.910721	3.012981	0.222248
C	-3.133743	2.183809	-0.243189
C	-0.438690	0.426925	-0.019089
C	-3.521483	0.973327	0.618532
C	-1.442328	-0.291081	0.034746
C	-2.918342	-0.344619	0.172680
C	-0.602438	2.828589	-0.563171
C	0.310004	1.687767	-0.080788
H	-2.996908	1.854380	-1.280717
H	-3.236053	1.125284	1.664493
H	-4.001314	2.849933	-0.258739
H	-4.604707	0.812412	0.595393
H	-0.012357	3.749949	-0.505160
H	-0.832661	2.671464	-1.623424
H	1.175249	1.596649	-0.746070
H	0.710810	1.907737	0.916883
H	-2.182813	4.070467	0.144423
H	-1.724002	2.834372	1.290047
F	-3.456380	-0.718858	-1.023920
F	-3.278986	-1.310562	1.064260
C	1.262927	-1.240102	-0.072788
C	-0.974789	-2.302047	-0.204747

N	0.879116	-1.695800	-1.305502
N	-0.267502	-2.262231	-1.376931
N	-0.307194	-2.470340	0.981733
N	0.841488	-1.907267	1.046390
H	-1.970840	-2.723666	-0.259587
C	2.511685	-0.457977	0.023874
C	2.985588	-0.058774	1.277079
C	3.200658	-0.081484	-1.133292
C	4.138951	0.711559	1.369767
H	2.444741	-0.364042	2.167343
C	4.354791	0.688532	-1.033142
H	2.825204	-0.403574	-2.099041
C	4.824686	1.087921	0.216056
H	4.506089	1.017290	2.344552
H	4.890703	0.973975	-1.932970
H	5.725285	1.689662	0.291024

TS-DIFO-4N-36Ph

G(water) = -1268.418607 Hartree

C	0.940458	3.934008	-0.194205
C	-0.597485	3.826379	-0.147069
C	0.799493	0.826632	-0.137167
C	-1.210376	2.951408	0.954990
C	-0.423510	0.744456	0.064515
C	-1.589759	1.543860	0.528512
C	1.698087	2.943498	-1.089227
C	2.023030	1.573330	-0.478795
H	-0.983604	3.503003	-1.121872
H	-0.541388	2.866685	1.817355
H	-0.986545	4.838278	0.001922
H	-2.148097	3.391049	1.311615
H	2.658305	3.391089	-1.370285
H	1.139854	2.791568	-2.020863
H	2.623809	0.995547	-1.189943
H	2.643421	1.683206	0.419745
H	1.179328	4.936244	-0.566186
H	1.345196	3.892643	0.826805
F	-2.535911	1.616687	-0.447129
F	-2.165814	0.930909	1.598556
C	1.462186	-1.284887	0.005493
C	-1.044184	-1.291792	-0.060204
N	0.882680	-1.633041	-1.194499
N	-0.388435	-1.651397	-1.227208
N	-0.454985	-1.653921	1.138400
N	0.817753	-1.642554	1.168916
C	2.939942	-1.222543	0.064791
C	3.570755	-0.964798	1.285456
C	3.707341	-1.390990	-1.091333
C	4.956132	-0.871173	1.346069
H	2.965240	-0.846964	2.178744
C	5.094060	-1.299021	-1.023380
H	3.207824	-1.602380	-2.031253
C	5.720783	-1.036500	0.192278
H	5.441098	-0.671298	2.296659
H	5.686046	-1.437237	-1.922785
H	6.802901	-0.964256	0.242344
C	-2.527378	-1.332419	-0.137563
C	-3.292713	-1.614921	0.995320
C	-3.155426	-1.116133	-1.365815
C	-4.676895	-1.685790	0.895028
H	-2.793757	-1.775068	1.944452
C	-4.540186	-1.182459	-1.459740
H	-2.550357	-0.897636	-2.239595
C	-5.303396	-1.469059	-0.330422
H	-5.269034	-1.910089	1.776797
H	-5.024089	-1.010756	-2.416143
H	-6.385477	-1.522125	-0.404808

TS-DIBO-3N-6Ph

G(water) = -1127.713992 Hartree

C	0.531245	1.223180	-0.438774
C	0.016202	2.393951	0.157950
C	0.760308	3.568802	0.077828
C	1.979691	3.601908	-0.593496
C	2.452232	2.458333	-1.232110
C	1.727072	1.276159	-1.162645
H	0.368012	4.472369	0.537580
H	2.548132	4.525901	-0.636883
H	3.382695	2.484559	-1.790000
H	2.069553	0.389043	-1.683413
C	-0.281541	0.028896	-0.366318
C	-1.402092	-0.543484	-0.375597
C	-2.821153	-0.365341	-0.071908
C	-3.646718	-1.429679	0.313983
C	-3.341666	0.950487	-0.060292
C	-4.976898	-1.213416	0.653478
H	-3.235660	-2.428704	0.405854
C	-4.679472	1.141495	0.278193
C	-5.502865	0.073136	0.620906
H	-5.595913	-2.053374	0.951905
H	-5.078226	2.152730	0.276002
H	-6.543311	0.249127	0.875844
C	-2.449686	2.125411	-0.355950
H	-1.948354	1.970112	-1.317346
H	-3.061494	3.025808	-0.461306
C	-1.095157	-2.468145	-0.835364
C	1.196437	-1.728273	-0.127777
N	1.025891	-2.036177	-1.465122
N	-0.130669	-2.426392	-1.831987
N	-0.768384	-2.911083	0.425203

C	-1.364967	2.357605	0.751429
H	-1.433338	1.553295	1.492251
H	-1.568584	3.294642	1.277343
C	2.508651	-1.209204	0.311633
C	2.603158	-0.549724	1.539952
C	3.653521	-1.370577	-0.474174
C	3.824671	-0.048827	1.976243
H	1.704613	-0.398928	2.134595
C	4.873264	-0.868696	-0.035321
H	3.569599	-1.884609	-1.426645
C	4.961673	-0.206123	1.188252
H	3.886716	0.473572	2.925766
H	5.760363	-0.998006	-0.648029
H	5.915522	0.186924	1.526414
H	-2.073517	-2.744270	-1.213986
C	0.413453	-2.508387	0.780481
H	0.809145	-2.791534	1.753781

TS-DIBO-3N-5Ph
G(water) = -1127.717425 Hartree

C	0.859207	2.011807	0.117834
C	1.806723	2.198635	-0.915637
C	2.136818	3.496525	-1.297192
C	1.570143	4.601274	-0.667332
C	0.678240	4.413274	0.383681
C	0.327151	3.126922	0.776336
H	2.859739	3.639658	-2.096119
H	1.840166	5.604004	-0.983613
H	0.259332	5.266954	0.906836
H	-0.323234	2.984944	1.633324
C	0.577044	0.643269	0.511470
C	0.962510	-0.544523	0.615781
C	1.862026	-1.594365	0.172045
C	1.518767	-2.952104	0.181860
C	3.100542	-1.193220	-0.381302
C	2.404245	-3.908878	-0.300297
H	0.536041	-3.256812	0.526218
C	3.974601	-2.170112	-0.851346
C	3.642203	-3.521173	-0.802791
H	2.121475	-4.956660	-0.287459
H	4.930345	-1.861039	-1.266929
H	4.341619	-4.265125	-1.171332
C	3.420915	0.271556	-0.502292
H	3.317045	0.748950	0.478085
H	4.464507	0.393413	-0.805903
C	-0.370081	-1.453659	2.055686
C	-1.427578	0.687001	1.379388
N	-0.939525	0.635116	2.660933
N	-0.389941	-0.466830	3.014149
N	-1.401228	-1.634538	1.186090

C	2.487507	1.003621	-1.525560
H	1.734405	0.297241	-1.891219
H	3.072240	1.320353	-2.393752
H	0.207272	-2.326813	2.338030
H	-1.786681	1.655712	1.054693
C	-1.982427	-0.513279	0.839526
C	-3.120204	-0.521416	-0.103154
C	-4.000602	0.561348	-0.191099
C	-3.325532	-1.637522	-0.922069
C	-5.060172	0.535089	-1.091889
H	-3.878640	1.418618	0.464157
C	-4.385585	-1.661828	-1.818994
H	-2.638979	-2.474078	-0.843878
C	-5.253917	-0.574758	-1.908941
H	-5.740383	1.379218	-1.147060
H	-4.534706	-2.529969	-2.453768
H	-6.081970	-0.595701	-2.610789

TS-DIBO-3N-3Ph
G(water) = -1127.713399 Hartree

C	0.552806	1.215226	-0.450669
C	0.032566	2.393338	0.127210
C	0.771457	3.570353	0.032954
C	1.991640	3.600843	-0.636714
C	2.469139	2.451049	-1.259555
C	1.749108	1.266691	-1.173864
H	0.374464	4.477665	0.481150
H	2.556438	4.526562	-0.690359
H	3.400662	2.472993	-1.815921
H	2.097894	0.375959	-1.682774
C	-0.247931	0.010409	-0.364356
C	-1.382509	-0.536594	-0.364099
C	-2.798912	-0.358374	-0.046098
C	-3.615034	-1.410782	0.389830
C	-3.324241	0.955096	-0.068406
C	-4.942757	-1.188650	0.734891
H	-3.196134	-2.402277	0.520425
C	-4.659977	1.152432	0.274562
C	-5.476264	0.093119	0.659361
H	-5.553650	-2.019736	1.072482
H	-5.061504	2.162138	0.244840
H	-6.515228	0.273148	0.917378
C	-2.431049	2.122130	-0.387891
H	-1.927703	1.949167	-1.345244
H	-3.039219	3.023058	-0.508977
C	-1.121476	-2.483730	-0.855106
C	1.185387	-1.752312	-0.181713
N	1.089243	-2.083192	-1.511565
N	0.448330	-2.455344	0.762135
N	-0.711313	-2.854047	0.415588

C	-1.348531	2.363682	0.719521
H	-1.418183	1.566061	1.467110
H	-1.552781	3.305588	1.236377
C	2.472151	-1.224503	0.331549
C	2.516492	-0.643769	1.600113
C	3.634507	-1.302284	-0.438143
C	3.709938	-0.122930	2.085638
H	1.605441	-0.596501	2.189903
C	4.828565	-0.785971	0.053211
H	3.585914	-1.763617	-1.419687
C	4.867112	-0.190039	1.311776
H	3.738293	0.339058	3.067621
H	5.731588	-0.849447	-0.546384
H	5.798776	0.218230	1.691695
H	-2.121993	-2.798318	-1.126991
C	-0.098036	-2.482015	-1.850604
H	-0.291792	-2.786121	-2.876141

TS-DIBO-4N-3Ph

G(water) = -1143.740254 Hartree

C	0.581167	1.215480	-0.489012
C	0.083978	2.391244	0.114112
C	0.845906	3.554313	0.031894
C	2.063809	3.569112	-0.642881
C	2.519236	2.419139	-1.282268
C	1.776088	1.248120	-1.213674
H	0.470893	4.462735	0.496298
H	2.646299	4.484266	-0.685807
H	3.450077	2.431808	-1.839809
H	2.104424	0.356289	-1.735098
C	-0.255785	0.040032	-0.408090
C	-1.385344	-0.501396	-0.382165
C	-2.795399	-0.334106	-0.051228
C	-3.619086	-1.396847	0.339279
C	-3.302899	0.986554	-0.029341
C	-4.942585	-1.172131	0.698616
H	-3.212400	-2.399188	0.418247
C	-4.633773	1.184622	0.330546
C	-5.458293	0.119084	0.680548
H	-5.564053	-2.008520	1.001472
H	-5.026734	2.197998	0.338867
H	-6.493485	0.300793	0.952109
C	-2.404417	2.152934	-0.343767
H	-1.930966	1.995541	-1.318993
H	-3.010045	3.059296	-0.431098
C	-1.099855	-2.445172	-0.926525
C	1.143624	-1.775215	-0.160003
N	1.029366	-2.069736	-1.499301
N	-0.127763	-2.437921	-1.897751
N	0.396079	-2.509758	0.733966

N	-0.762098	-2.874747	0.338094
C	-1.287361	2.374115	0.733627
H	-1.348953	1.576087	1.481828
H	-1.465538	3.316449	1.259041
C	2.428368	-1.254266	0.351672
C	2.467404	-0.672841	1.619746
C	3.592731	-1.336732	-0.415438
C	3.660143	-0.153840	2.109233
H	1.554373	-0.622354	2.206625
C	4.785040	-0.821902	0.080804
H	3.549231	-1.800259	-1.396170
C	4.819320	-0.224612	1.339069
H	3.686200	0.308472	3.090986
H	5.690452	-0.888493	-0.514553
H	5.750559	0.181539	1.722046
H	-2.091516	-2.701463	-1.279561

TS-DIBO-4N-36Ph

G(water) = -1374.680356 Hartree

C	1.748951	1.107429	-0.633059
C	1.792705	2.486835	-0.336650
C	2.840663	3.250517	-0.845538
C	3.820119	2.682681	-1.654320
C	3.736200	1.336191	-1.995816
C	2.703209	0.555138	-1.494427
H	2.877156	4.310979	-0.609799
H	4.632199	3.295083	-2.033972
H	4.470829	0.890719	-2.658860
H	2.614542	-0.483070	-1.791430
C	0.616985	0.338721	-0.129802
C	-0.616995	0.338714	0.129799
C	-1.748966	1.107409	0.633062
C	-2.703196	0.555116	1.494459
C	-1.792745	2.486810	0.336634
C	-3.736184	1.336166	1.995864
H	-2.614508	-0.483087	1.791476
C	-2.840699	3.250487	0.845536
C	-3.820128	2.682650	1.654351
H	-4.470788	0.890695	2.658936
H	-2.877211	4.310946	0.609782
H	-4.632204	3.295048	2.034017
C	-0.660833	3.120611	-0.421714
H	-0.487112	2.578802	-1.357892
H	-0.925324	4.146537	-0.692502
C	-1.220321	-1.650721	-0.224417
C	1.220333	-1.650715	0.224424
N	0.843528	-2.062973	-1.044500
N	-0.405353	-2.056129	-1.275893
N	0.405366	-2.056116	1.275904
N	-0.843515	-2.062966	1.044511

C	0.660775	3.120640	0.421670
H	0.487065	2.578866	1.357870
H	0.925244	4.146581	0.692419
C	2.664906	-1.623127	0.566139
C	3.071155	-0.943642	1.714734
C	3.606147	-2.286494	-0.222054
C	4.417016	-0.903137	2.059085
H	2.324831	-0.442851	2.325868
C	4.951339	-2.248838	0.128963
H	3.274980	-2.824543	-1.105171
C	5.359299	-1.552971	1.264725
H	4.731114	-0.363600	2.947065
H	5.682452	-2.767621	-0.483394
H	6.410718	-1.521406	1.533806
C	-2.664893	-1.623134	-0.566137
C	-3.071128	-0.943674	-1.714753
C	-3.606144	-2.286478	0.222061
C	-4.416985	-0.903170	-2.059118
H	-2.324796	-0.442903	-2.325894
C	-4.951333	-2.248822	-0.128968
H	-3.274986	-2.824510	1.105192
C	-5.359278	-1.552980	-1.264751
H	-4.731073	-0.363653	-2.947113
H	-5.682454	-2.767585	0.483396
H	-6.410695	-1.521419	-1.533841

TS-TMTH-3N-6Ph

G(water) = -1299.688203 Hartree

C	-0.926110	-2.442779	0.291330
C	1.330255	-1.287520	0.187873
N	0.938958	-2.028679	-0.902499
N	-0.190325	-2.624697	-0.860649
N	-0.331486	-2.319678	1.513752
C	-1.536748	-0.522933	-0.120705
C	-1.325445	2.529520	-0.509960
C	-0.566962	0.257000	-0.109804
C	-0.113641	1.678156	-0.047899
C	-3.516366	0.803485	-0.688836
C	-3.032431	-0.597835	-0.252688
H	-1.462641	2.414147	-1.591341
H	-1.094056	3.583240	-0.317314
H	-4.610453	0.829986	-0.633456
S	-2.929817	2.221069	0.293014
C	0.245905	2.035163	1.399115
H	1.077712	1.420011	1.749760
H	0.554006	3.085537	1.455075
H	-0.611613	1.878733	2.059710
C	1.044819	2.028578	-0.996173
H	1.065295	3.113431	-1.150410
H	2.011316	1.735366	-0.585441
H	0.918276	1.544527	-1.969429
C	-3.663897	-0.960505	1.097463
H	-4.751638	-1.034361	0.986906
H	-3.294765	-1.915501	1.480982
H	-3.436581	-0.189684	1.839398
C	-3.472167	-1.574742	-1.354826
H	-3.292768	-2.618853	-1.088085
H	-4.547199	-1.460803	-1.532259
H	-2.940260	-1.370853	-2.288721
H	-3.234375	0.983090	-1.732620
C	2.634013	-0.593399	0.053310
C	3.242797	0.036249	1.144546
C	3.276516	-0.557336	-1.190726
C	4.449997	0.711199	0.990931
H	2.772236	0.019226	2.122952
C	4.481103	0.117112	-1.341757
H	2.809299	-1.057047	-2.031478
C	5.070229	0.760102	-0.254023
H	4.904576	1.200124	1.846954
H	4.963205	0.143542	-2.314251
H	6.009664	1.290445	-0.375221
H	-1.882869	-2.950942	0.284468
C	0.818297	-1.715369	1.448620
H	1.389689	-1.593979	2.366632

TS-TMTH-3N-5Ph

G(water) = -1299.697382 Hartree

C	-0.924949	-0.245709	1.628736
C	0.109380	-2.336255	0.716822
N	-1.056033	-2.019821	0.112562
N	0.400002	-2.028675	2.016201
N	-0.147369	-0.960719	2.485881
C	0.794472	0.419433	0.269312
C	3.280214	-0.003764	-1.458866
C	1.372933	-0.608927	-0.096797
C	2.599053	-1.182885	-0.716216
C	1.880486	2.339180	-0.702310
C	0.788993	1.906868	0.310215
H	2.709713	0.234157	-2.364157
H	4.283607	-0.314482	-1.772450
H	2.057655	3.415837	-0.597892
S	3.521280	1.551831	-0.533392
C	3.511115	-1.729445	0.388432
H	3.000603	-2.507092	0.964995
H	4.417895	-2.158363	-0.053083
H	3.794161	-0.929269	1.078163
C	2.308486	-2.265573	-1.764547
H	3.226601	-2.499621	-2.314399
H	1.958367	-3.192081	-1.303136
H	1.551098	-1.929123	-2.478958

C	1.151719	2.404700	1.714217
H	1.155585	3.500434	1.729130
H	0.434097	2.053094	2.460555
H	2.142594	2.042443	2.001857
C	-0.545364	2.504014	-0.157081
H	-1.346789	2.314942	0.562980
H	-0.447209	3.589455	-0.269650
H	-0.852436	2.079059	-1.118024
H	1.525713	2.158162	-1.723490
H	-1.281903	0.700368	2.020196
H	0.657568	-3.184947	0.321457
C	-1.613432	-0.929547	0.588620
C	-2.870983	-0.425035	-0.001122
C	-3.699905	0.460408	0.695708
C	-3.232746	-0.829304	-1.291168
C	-4.863273	0.944949	0.107182
H	-3.453902	0.751421	1.712953
C	-4.396274	-0.345183	-1.875685
H	-2.584197	-1.520554	-1.819385
C	-5.212290	0.545756	-1.180214
H	-5.503038	1.625758	0.659895
H	-4.667379	-0.660337	-2.878599
H	-6.120905	0.923862	-1.638615

TS-TMTH-3N-3Ph

G(water) = -1299.688723 Hartree

C	-0.960388	-2.460106	0.252390
C	1.299314	-1.319928	0.173608
N	0.919196	-2.060469	-0.923417
N	-0.205367	-2.665943	-0.880278
N	0.895933	-1.658063	1.436284
C	-1.523454	-0.514974	-0.143610
C	-1.277274	2.545516	-0.465845
C	-0.531571	0.240502	-0.153865
C	-0.077050	1.661640	-0.040502
C	-3.483984	0.846492	-0.678335
C	-3.021996	-0.567598	-0.261432
H	-1.421117	2.473779	-1.550238
H	-1.031052	3.588105	-0.234928
H	-4.577989	0.886607	-0.631505
S	-2.883622	2.236015	0.332406
C	0.280021	1.941947	1.425420
H	1.076957	1.274242	1.760159
H	0.628576	2.976106	1.528584
H	-0.592167	1.793677	2.068701
C	1.090127	2.056680	-0.960469
H	1.101787	3.146931	-1.071847
H	2.053323	1.757257	-0.547390
H	0.982712	1.611298	-1.954227
C	-3.658262	-0.934690	1.084861
H	-4.749905	-0.927682	0.991052
H	-3.361589	-1.932746	1.419967
H	-3.367735	-0.212282	1.853209
C	-3.486669	-1.524123	-1.371237
H	-3.318324	-2.573714	-1.118883
H	-4.561497	-1.393424	-1.537970
H	-2.960225	-1.316278	-2.307294
H	-3.190638	1.037677	-1.716817
C	2.597453	-0.614045	0.034386
C	3.235819	-0.084043	1.158623
C	3.194281	-0.478625	-1.223966
C	4.430094	0.615908	1.019905
H	2.788555	-0.231167	2.136209
C	4.391204	0.213941	-1.357077
H	2.702077	-0.911036	-2.087923
C	5.006595	0.774279	-0.238464
H	4.914578	1.033268	1.897330
H	4.844742	0.321394	-2.337665
H	5.938185	1.321732	-0.345757
H	-1.904394	-2.990620	0.257542
C	-0.250532	-2.267861	1.471567
H	-0.647625	-2.603516	2.427318

TS-TMTH-4N-3Ph

G(water) = -1315.71385 Hartree

C	-0.906054	-2.449254	0.345471
C	1.308692	-1.347496	0.163169
N	0.931308	-2.135749	-0.889470
N	-0.206253	-2.718357	-0.797656
N	0.878050	-1.646226	1.428276
N	-0.254327	-2.236954	1.528890
C	-1.518945	-0.493706	-0.130518
C	-1.276391	2.548600	-0.477098
C	-0.540438	0.267880	-0.134555
C	-0.074852	1.682193	-0.011201
C	-3.475249	0.815511	-0.726818
C	-3.010375	-0.586200	-0.267500
H	-1.382606	2.470365	-1.565279
H	-1.049171	3.594648	-0.242527
H	-4.570171	0.849256	-0.699667
S	-2.905897	2.229929	0.272006
C	0.241617	1.965191	1.462630
H	1.035811	1.303310	1.816654
H	0.579002	3.001938	1.574681
H	-0.644836	1.807320	2.083326
C	1.111273	2.080625	-0.903904
H	1.125495	3.171752	-1.004782
H	2.065938	1.777151	-0.473683
H	1.023341	1.644971	-1.903781
C	-3.660083	-0.922383	1.080897
H	-4.747967	-0.969706	0.959581
H	-3.323193	-1.884349	1.475456

H	-3.421104	-0.152475	1.819673
C	-3.434169	-1.582514	-1.356657
H	-3.222508	-2.618784	-1.081122
H	-4.513142	-1.498688	-1.525813
H	-2.915693	-1.374113	-2.297082
H	-3.163228	0.987281	-1.763248
C	2.592410	-0.626550	0.025216
C	3.220816	-0.076196	1.145969
C	3.180581	-0.491115	-1.236337
C	4.402755	0.641606	0.997197
H	2.777988	-0.220353	2.125927
C	4.364033	0.222658	-1.378436
H	2.694059	-0.939526	-2.095805
C	4.971921	0.800651	-0.264911
H	4.883056	1.073978	1.869454
H	4.812457	0.332580	-2.360929
H	5.893131	1.364030	-0.378698
H	-1.869013	-2.935444	0.429606

TS-TMTH-4N-36Ph

G(water) = -1546.642471 Hartree

C	1.286462	-1.307393	0.019521
C	-1.214309	-1.322020	-0.084784
N	-0.645081	-1.785595	1.088482
N	0.622514	-1.784297	1.139129
N	-0.542269	-1.558235	-1.272877
N	0.724095	-1.550079	-1.220509
C	0.657822	0.780078	0.071133
C	-1.145609	2.985821	1.021337
C	-0.579748	0.739913	0.287935
C	-1.715694	1.562767	0.848094
C	1.309856	3.171854	-0.170216
C	1.731378	1.705684	-0.463904
H	-0.383321	2.994715	1.808346
H	-1.955650	3.652879	1.333153
H	1.939124	3.817284	-0.792356
S	-0.397461	3.697736	-0.477476
C	-2.962397	1.656281	-0.048527
H	-3.624037	0.800488	0.073330
H	-3.528133	2.550792	0.234447
H	-2.688069	1.748260	-1.103039
C	-2.092085	1.052431	2.246535
H	-2.848411	1.715142	2.682160
H	-2.508215	0.045338	2.201960
H	-1.217344	1.035004	2.903638
C	1.809919	1.485928	-1.981112
H	2.578932	2.141161	-2.405832
H	2.071760	0.450094	-2.212210
H	0.847433	1.711398	-2.450302
C	3.133995	1.602400	0.166495
H	3.730810	0.793250	-0.249684
H	3.668198	2.537399	-0.037373
H	3.075972	1.477134	1.251423
H	1.539366	3.410648	0.874333
C	2.764478	-1.421393	0.101249
C	3.532416	-1.614007	-1.050101
C	3.389480	-1.358282	1.349190
C	4.918233	-1.692916	-0.954342
H	3.036787	-1.704771	-2.010666
C	4.773617	-1.436232	1.439480
H	2.780355	-1.238034	2.239361
C	5.542481	-1.592286	0.287307
H	5.511455	-1.838202	-1.852016
H	5.255094	-1.373365	2.410512
H	6.624452	-1.648630	0.358547
C	-2.690364	-1.455375	-0.205515
C	-3.301742	-1.231532	-1.443303
C	-3.468833	-1.848098	0.886165
C	-4.679886	-1.348120	-1.572019
H	-2.687077	-0.955816	-2.293306
C	-4.849499	-1.958902	0.754043
H	-2.983310	-2.076102	1.829162
C	-5.459547	-1.699417	-0.470847
H	-5.148364	-1.163071	-2.533639
H	-5.448477	-2.257942	1.608602
H	-6.537315	-1.784498	-0.571409

References

1. Kamber, D. N.; Liang, Y.; Blizzard, R. J.; Liu, F.; Mehl, R. A.; Houk, K. N.; Prescher, J. A: 1,2,4-Triazines are versatile bioorthogonal reagents. *J. Am. Chem. Soc.* **2015**, *137*, 8388.
2. Ernd, M.; Heuschmann, M.; Zipse, H: Cycloadditions of Aryl-Substituted 1,2,4- Triazines with 2-Cyclopropylidene-1,3-dimethylimidazolidine – Zwitterions as Discrete Intermediates. *Helvetica. Chimica. Acta.* **2005**, *88*, 1491.
3. Yang, J.; Seckute, J.; Cole, C. M.; Devaraj, N. K: Live-cell imaging of cyclopropene tags with fluorogenic tetrazine cycloadditions. *Angew. Chem. Int. Ed.* **2012**, *51*, 7476.
4. Kamber, D. N.; Nazarova, L. A.; Liang, Y.; Lopez, S. A.; Patterson, D. M.; Shih, H. W.; Houk, K. N.; Prescher, J. A: Isomeric cyclopropenes exhibit unique bioorthogonal reactivities. *J. Am. Chem. Soc.* **2013**, *135*, 13680.

5. de Almeida, G.; Sletten, E. M.; Nakamura, H.; Palaniappan, K. K.; Bertozzi, C. R: Thiacycloalkynes for copper-free click chemistry. *Angew. Chem. Int. Ed.* **2012**, *51*, 2443.
6. Bertozzi, C. R.; Agard, N. J.; Prescher, J. A.; Baskin, J. M.; Sletten, E. M. Compositions and methods for modification of biomolecules. U.S. Patent 9,260,371, February 16, 2016.
7. Zhuo, J.; Zhang, C.; Xu, M.; Qian, D. Q.; Yao, W.; Jalluri, R. K. Triazolotriazines as kinase inhibitors. U.S. Patent 7,682,060, March 23, 2010.
8. Patterson, D. M.; Jones, K. A.; Prescher, J. A. Improved cyclopropene reporters for probing protein glycosylation. *Mol. BioSyst.* **2014**, *10*, 1693.
9. Row, R. D.; Prescher, J. A. A cyclopropenethione-phosphine ligation for rapid biomolecule labeling. *Org. Lett.* **2018**, *20*, 5614.
10. Elliott, S. T.; Townsley, F. M.; Bianco, A.; Ernst, R. J.; Sachdeva, A.; Elässer, S. J.; Davis, L.; Lang, K.; Pisa, R.; Greiss, S.; Lilley, K. S.; Chin, J. W. Proteome labeling and protein identification in specific tissues and at specific developmental stages in an animal. *Nat. Biotechnol.* **2014**, *32*, 465.
11. Yang, J.; Karver, M. R.; Li, W.; Sahu, S.; Deveraj, N. K., Metal-Catalyzed One-Pot Synthesis of Tetrazines Directly from Aliphatic Nitriles and Hydrazines. *Angew. Chem. Int. Ed.* **2012,** *51*, 5222–5225.
12. Engelen, W.; van de Wiel, K. M.; Meijer, L. H. H.; Saha, B.; Merkx, M. Nucleic acid detection using BRET-beacons based on bioluminescent protein-DNA hybrids. *Chem. Commun.* **2017**, *53*, 2862.
13. Hammill, J. T.; Miyake-Stoner, S.; Hazen, J. L.; Jackson, J. C.; Mehl, R. A. Preparation of site-specifically labeled fluorinated proteins for ^{19}F-NMR structural characterization. *Nat. Protoc.* **2007**, *2*, 2601.
14. Pedelacq, J.-D.; Cabantous, S.; Tran, T.; Terwilliger, T. C.; Waldo, G. S. Engineering and characterization of a superfolder green fluorescent protein. *Nat. Biotechnol.* **2006**, *24*, 79.
15. Frisch, M. J.; Trucks, G. W.; Schlegel, H. B.; Scuseria, G. E.; Robb, M. A.; Cheeseman, J. R.; Scalmani, G.; Barone, V.; Mennucci, B.; Petersson, G. A.; Nakatsuji, H.; Caricato, M.; Li, X.; Hratchian, H. P.; Izmaylov, A. F.; Bloino, J.; Zheng, G.; Sonnenberg, J. L.; Hada, M.; Ehara, M.; Toyota, K.; Fukuda, R.; Hasegawa, J.; Ishida, M.; Nakajima, T.; Honda, Y.; Kitao, O.; Nakai, H.; Vreven, T.; Montgomery, J. A., Jr.; Peralta, J. E.; Ogliaro, F.; Bearpark, M.; Heyd, J. J.; Brothers, E.; Kudin, K. N.; Staroverov, V. N.; Keith, T.; Kobayashi, R.; Normand, J.; Raghavachari, K.; Rendell, A.; Burant, J. C.; Iyengar, S. S.; Tomasi, J.; Cossi, M.; Rega, N.; Millam, J. M.; Klene, M.; Knox, J. E.; Cross, J. B.; Bakken, V.; Adamo, C.; Jaramillo, J.; Gomperts, R.; Stratmann, R. E.; Yazyev, O.; Austin, A. J.; Cammi, R.; Pomelli, C.; Ochterski, J. W.; Martin, R. L.; Morokuma, K.; Zakrzewski, V. G.; Voth, G. A.; Salvador, P.; Dannenberg, J. J.; Dapprich, S.; Daniels, A. D.; Farkas, O.; Foresman, J. B.; Ortiz, J. V.; Cioslowski, J.; Fox, D. J.; *Gaussian 09, revision D.01*; Gaussian Inc.: Wallingford, CT, 2013.
16. (a) Zhao, Y.; Truhlar, D. G. *Theor. Chem. Acc.* **2008**, *120*, 215. (b) Zhao, Y.; Truhlar, D. G. *Acc. Chem. Res.* **2008**, *41*, 157.
17. Hehre, W. J.; Radom, L.; Schleyer, P. v. R.; Pople, J. A. *Ab Initio Molecular Orbital Theory*, Wiley: New York, 1986.
18. (a) Zhao, Y.; Truhlar, D. G. *Phys. Chem. Chem. Phys.* **2008**, *10*, 2813. (b) Ribeiro, R. F.; Marenich, A. V.; Cramer, C. J.; Truhlar, D. G. *J. Phys. Chem. B* **2011**, *115*, 14556.
19. (a) Barone, V.; Cossi, M. *J. Phys. Chem. A* **1998**, *102*, 1995. (b) Cossi, M.; Rega, N.; Scalmani, G.; Barone, V. *J. Comput. Chem.* **2003**, *24*, 669. (c) Takano, Y.; Houk, K. N. *J. Chem. Theory Comput.* **2005**, *1*, 70.
20. Patterson, D. M.; Nazarova, L. A.; Xie, B. J.; Kamber, D. N.; Prescher, J. A. Functionalized cyclopropenes as bioorthogonal chemical reporters. *J. Am. Chem. Soc.* **2012**, *134*, 18638.

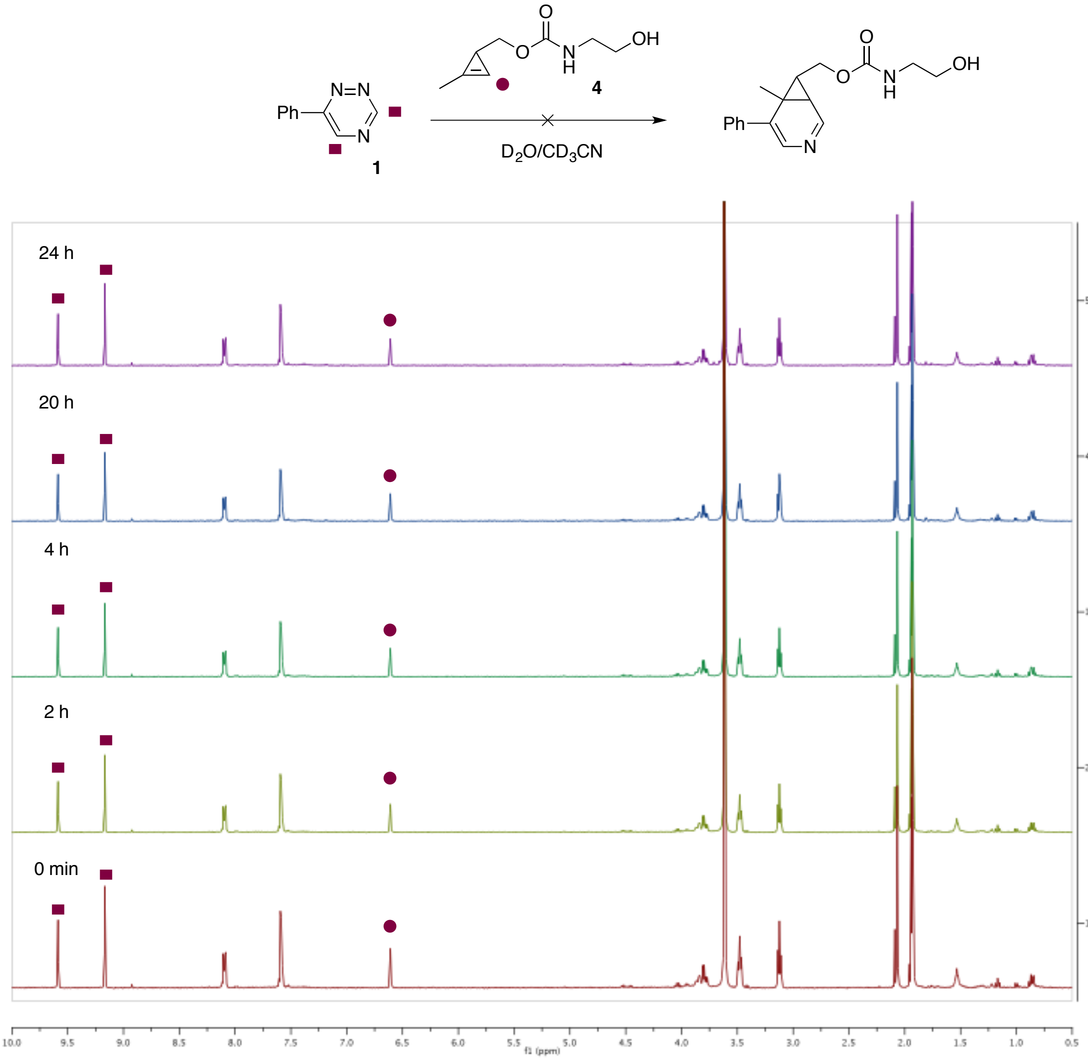

Figure S1. Triazine **1** is orthogonal to cyclopropene **4**. Triazine **1** (0.24 mL of a 25 mM solution in CD_3CN) was added a solution of cyclopropene **4** (0.24 mL of a 25 mM solution in 25% D_2O in CD_3CN) and diluted with 0.12 mL CD_3CN. The reaction was monitored over time by ^{1}H-NMR spectroscopy.

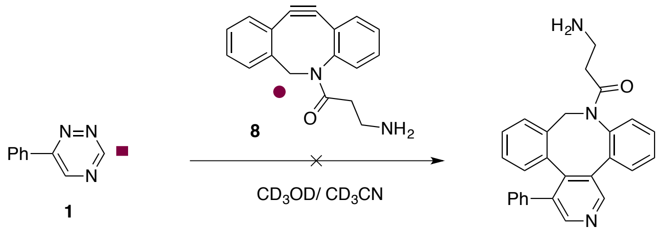

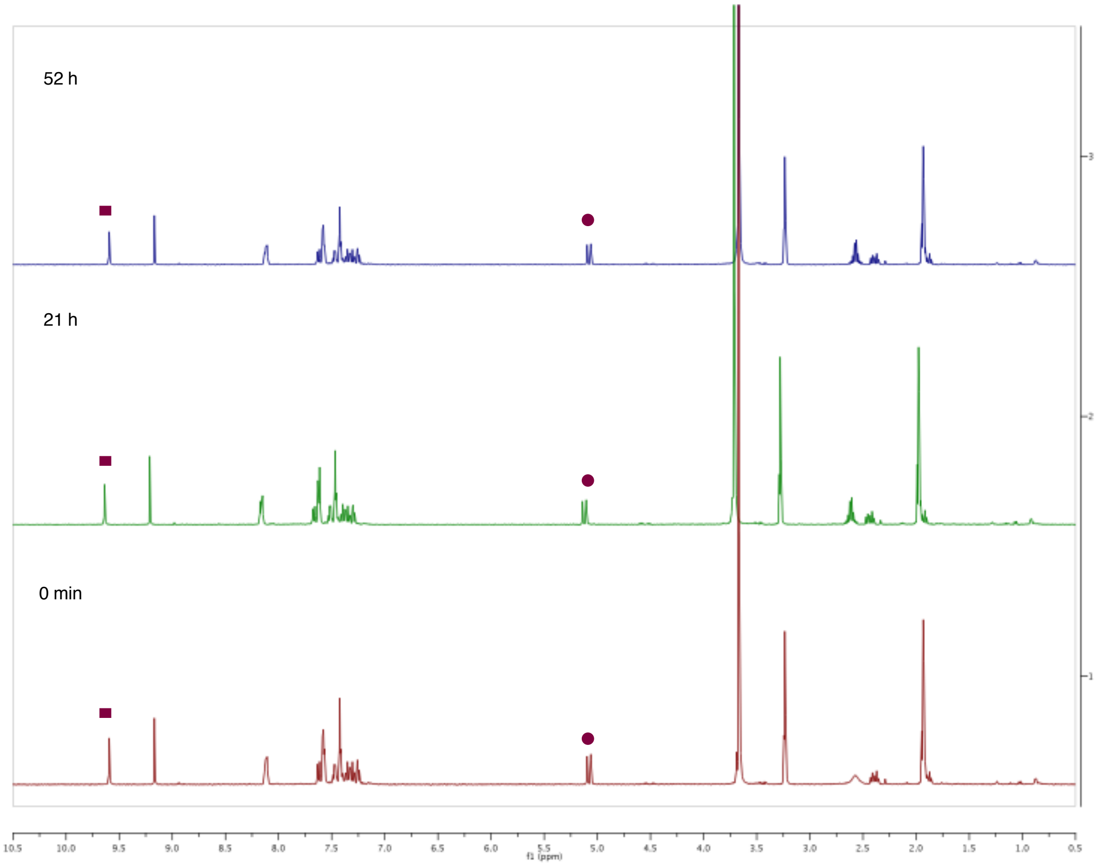

Figure S2. Triazine **1** is orthogonal to DBCO-amine **8**. Triazine **1** (0.24 mL of a 25 mM solution in CD_3CN) was added a solution of **8** (0.24 mL of a 25 mM solution in CD_3OD), and diluted to 0.6 mL with CD_3CN. The reaction was monitored over time by ^{1}H-NMR spectroscopy.

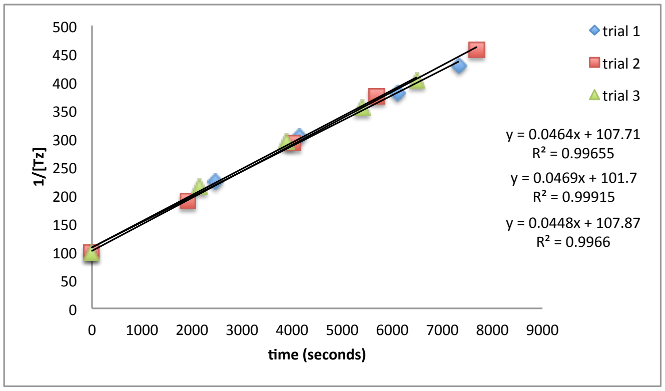

Figure S3. Kinetic data used to calculate second-order rate constants (k_2) for **2** and **7** in 1:1 CD_3CN: *d*-PBS. The reactions between triazine **2** and TCO **7** were run in 1:1 ratios and monitored by ^{1}H-NMR spectroscopy.

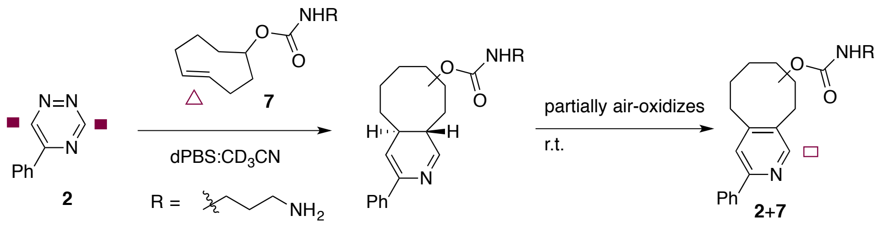

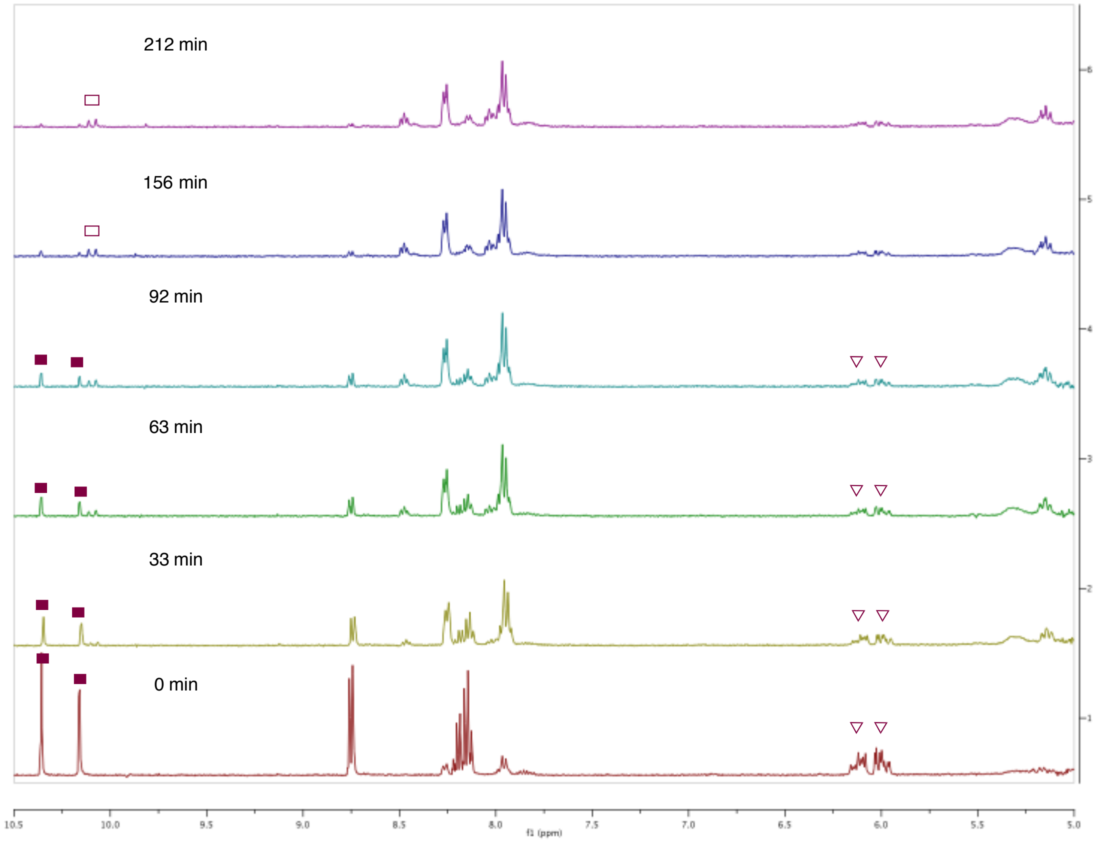

Figure S4. Reaction between triazine **2** and TCO. Triazine **2** (0.30 mL of a 20 mM solution in CD_3CN) was added a solution of TCO **7** (0.30 mL of a 20 mM solution in dPBS). The reaction was monitored over time by ^{1}H-NMR spectroscopy.

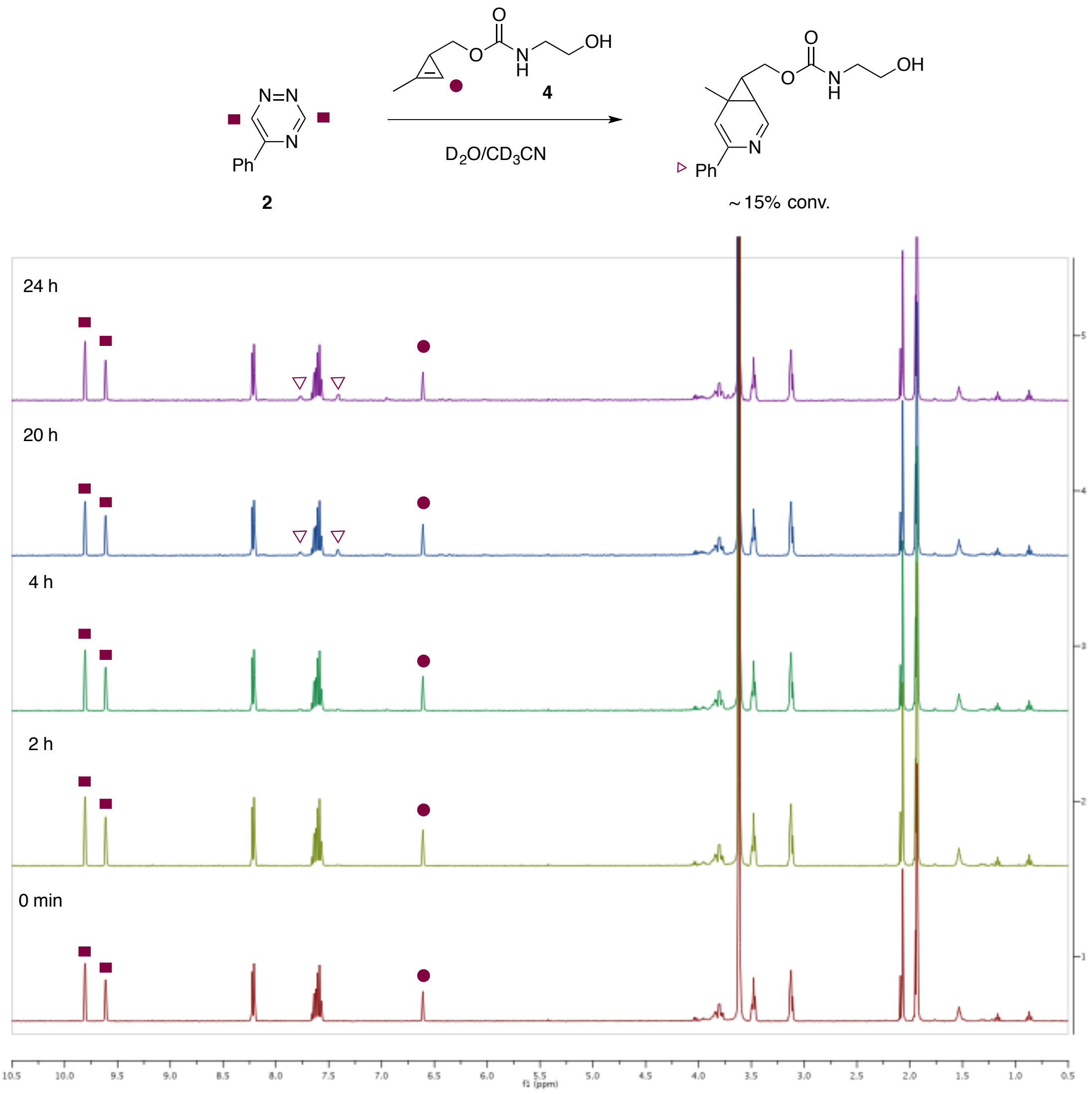

Figure S5. Minimum reactivity is observed between triazine **2** and cyclopropene **4**. Triazine **2** (0.30 mL of a 20 mM solution in CD_3CN) was added a solution of cyclopropene **4** (0.24 mL of a 25 mM solution in 25% D_2O in CD_3CN) and diluted with 60 µL CD_3CN. The reaction was monitored over time by ^{1}H-NMR spectroscopy.

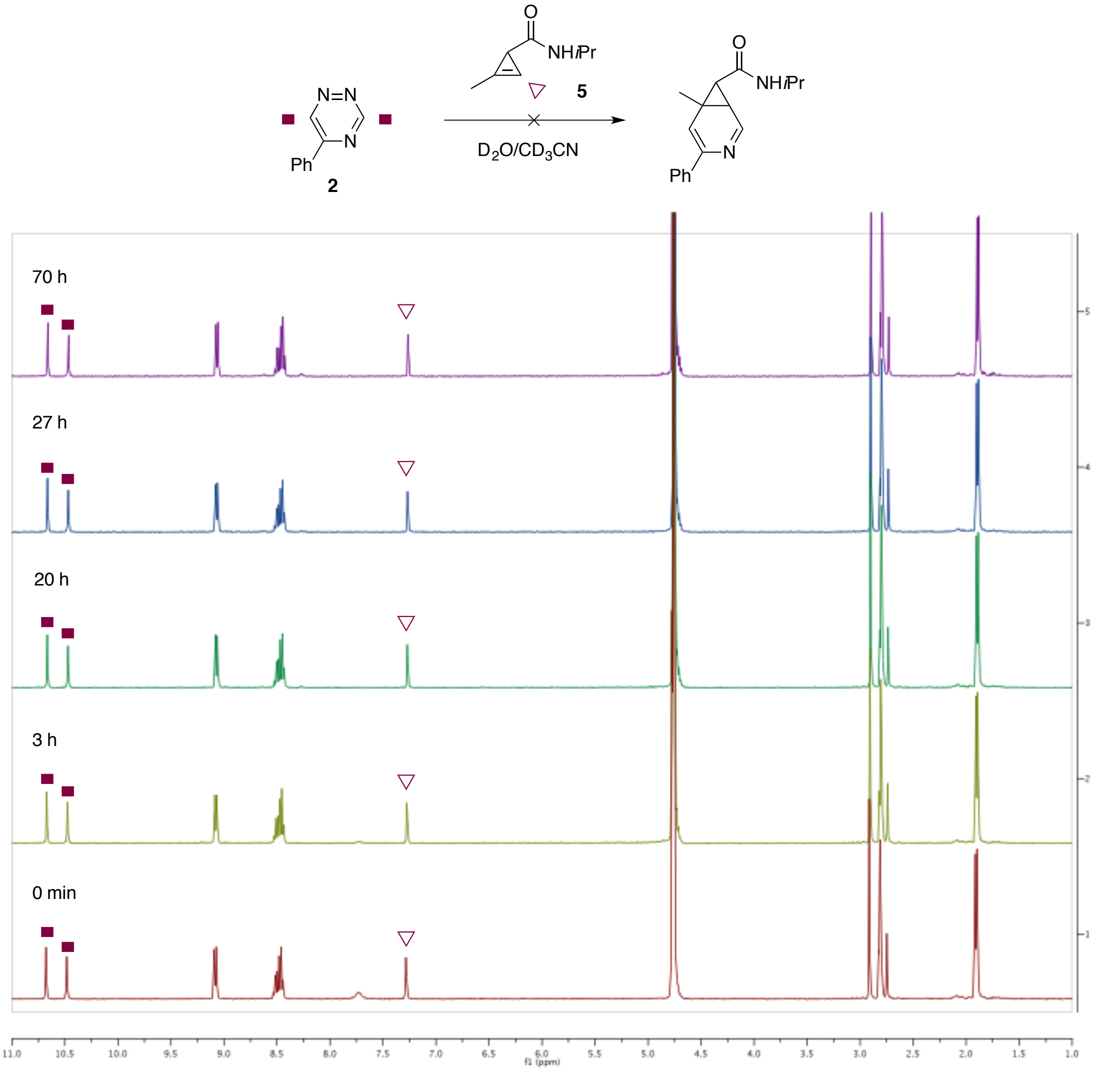

Figure S6. Triazine **2** is orthogonal to cyclopropene **5**. Triazine **2** (0.24 mL of a 25 mM solution in CD_3CN) was added a solution of cyclopropene **5** (0.17 mL of a 35 mM solution in CD_3CN) and diluted with 0.19 mL of D_2O. The reaction was monitored over time by ^{1}H-NMR spectroscopy.

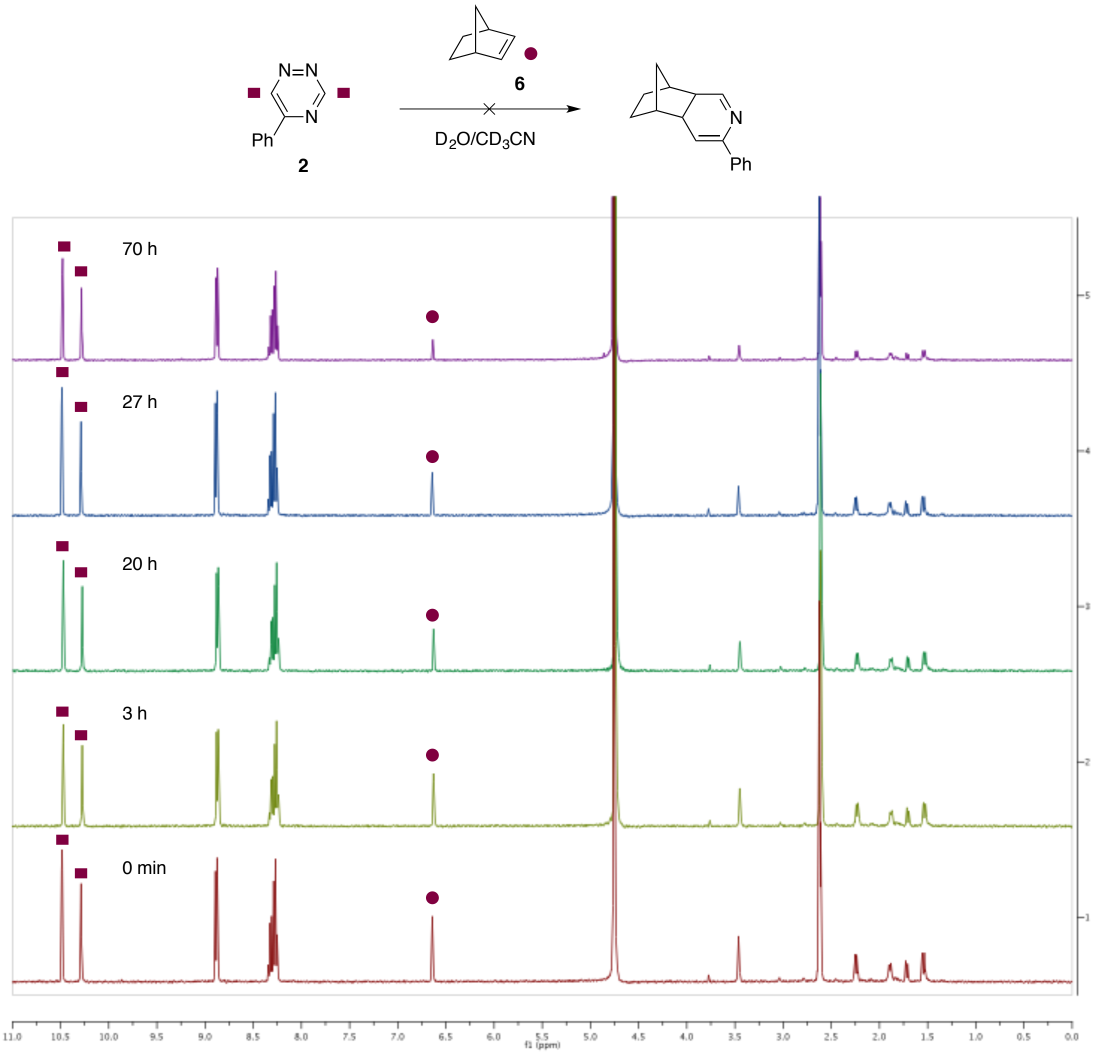

Figure S7. Triazine **2** is orthogonal to norbornene **6**. Triazine **2** (0.24 mL of a 25 mM solution in CD_3CN) was added a solution of norbornene **6** (0.24 mL of a 9.4 mM solution in 1:1 D_2O:CD_3CN) and diluted with 0.12 mL of D_2O. The reaction was monitored over time by ^{1}H-NMR spectroscopy.

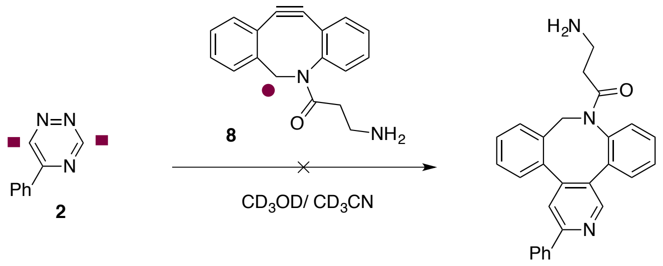

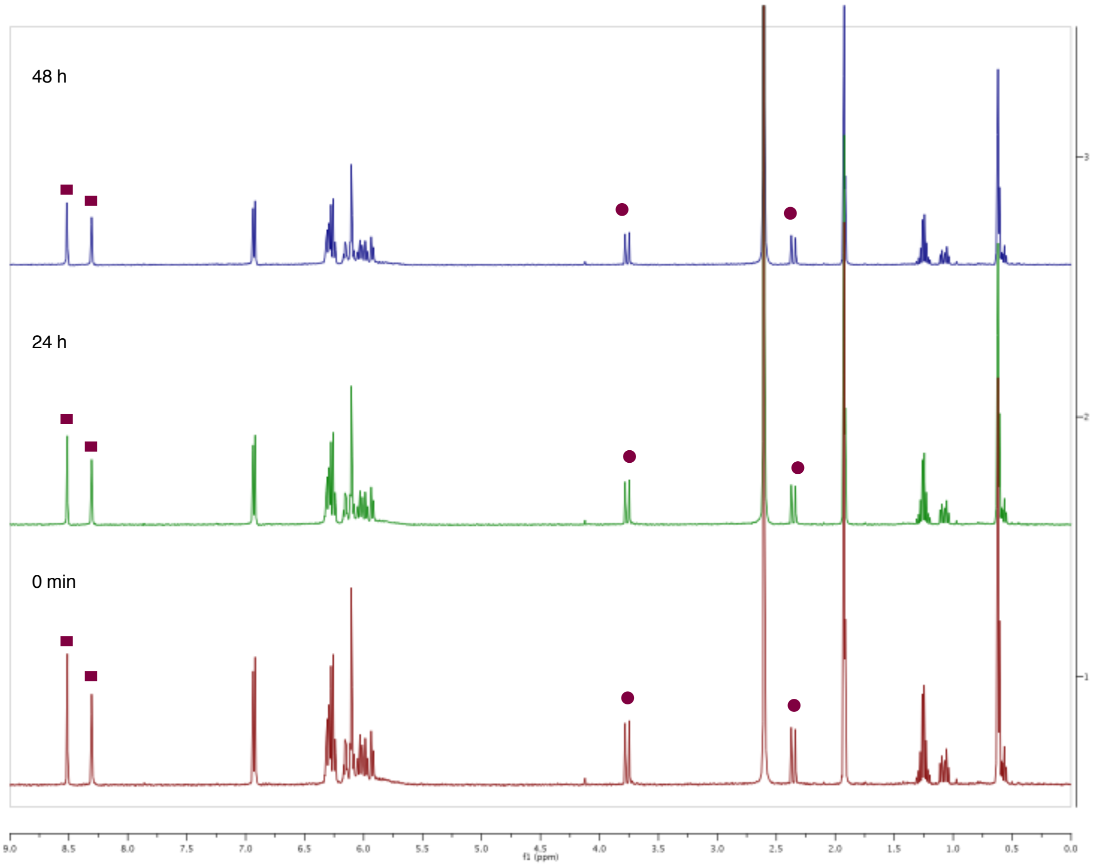

Figure S8. Triazine **2** is orthogonal to DBCO-amine **8**. Triazine **2** (0.30 mL of a 20 mM solution in CD_3CN) was added a solution of DBCO-amine **8** (0.30 mL of a 20 mM solution in CD_3OD). The reaction was monitored over time by ^{1}H-NMR spectroscopy.

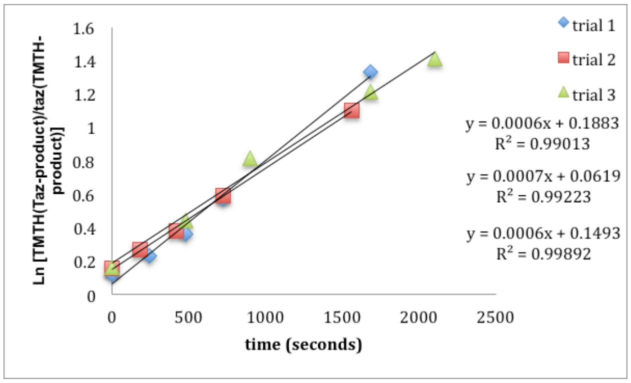

Figure S9. Kinetic data used to calculate second-order rate constants (k_2) for **2** and **9** CD_3CN. The reactions between triazine **2** and TMTH **9** were run in ~1:2 (triazine:TMTH) ratios and monitored by ^{1}H-NMR spectroscopy.

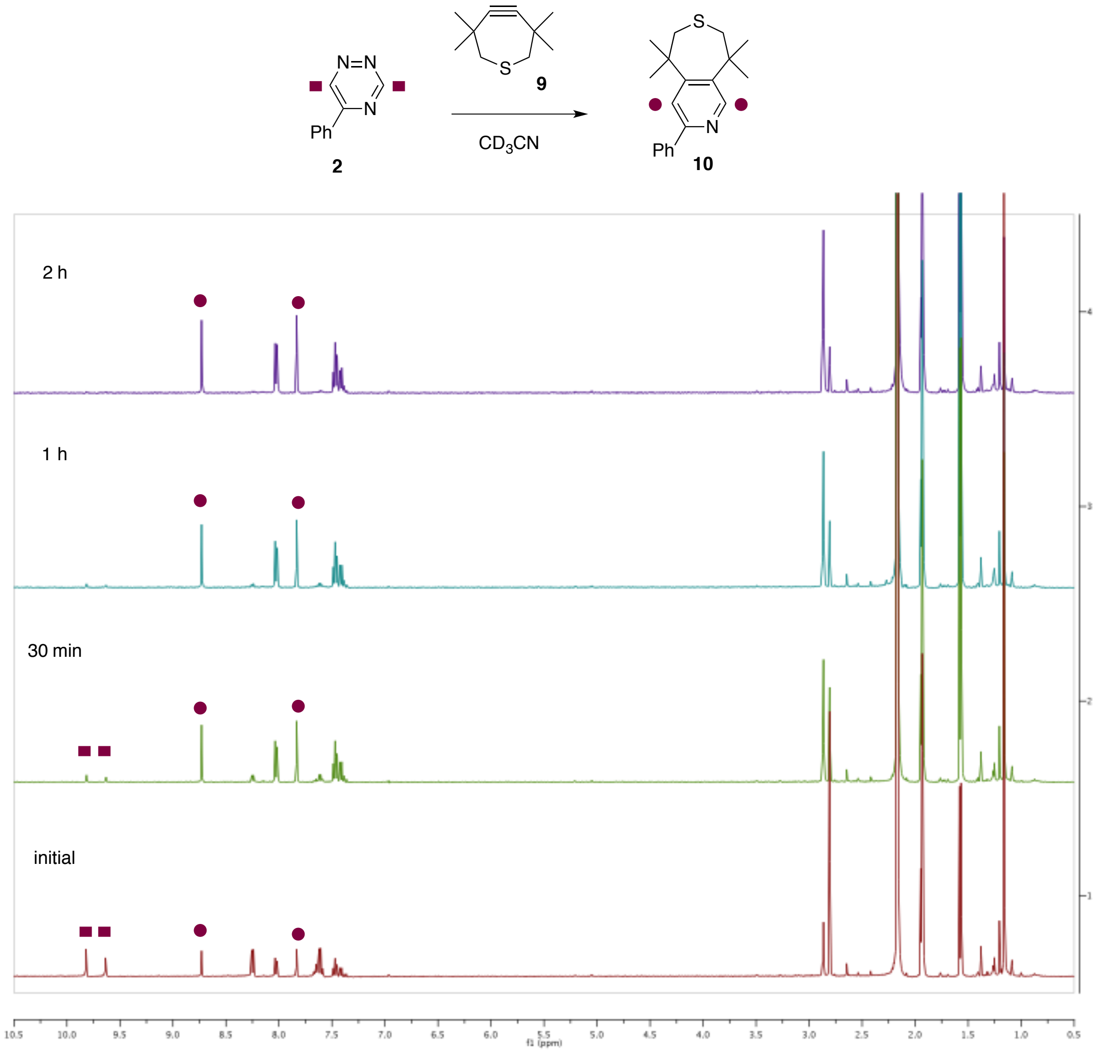

Figure S10. Reaction between triazine and TMTH. Triazine **2** (0.12 mL of a 50 mM solution in CD_3CN) was added a solution of TMTH **9** (0.12 mL of a 50 mM solution in CD_3CN) and diluted with 0.36 mL of CD_3CN. The reaction was monitored over time by ^{1}H-NMR spectroscopy.

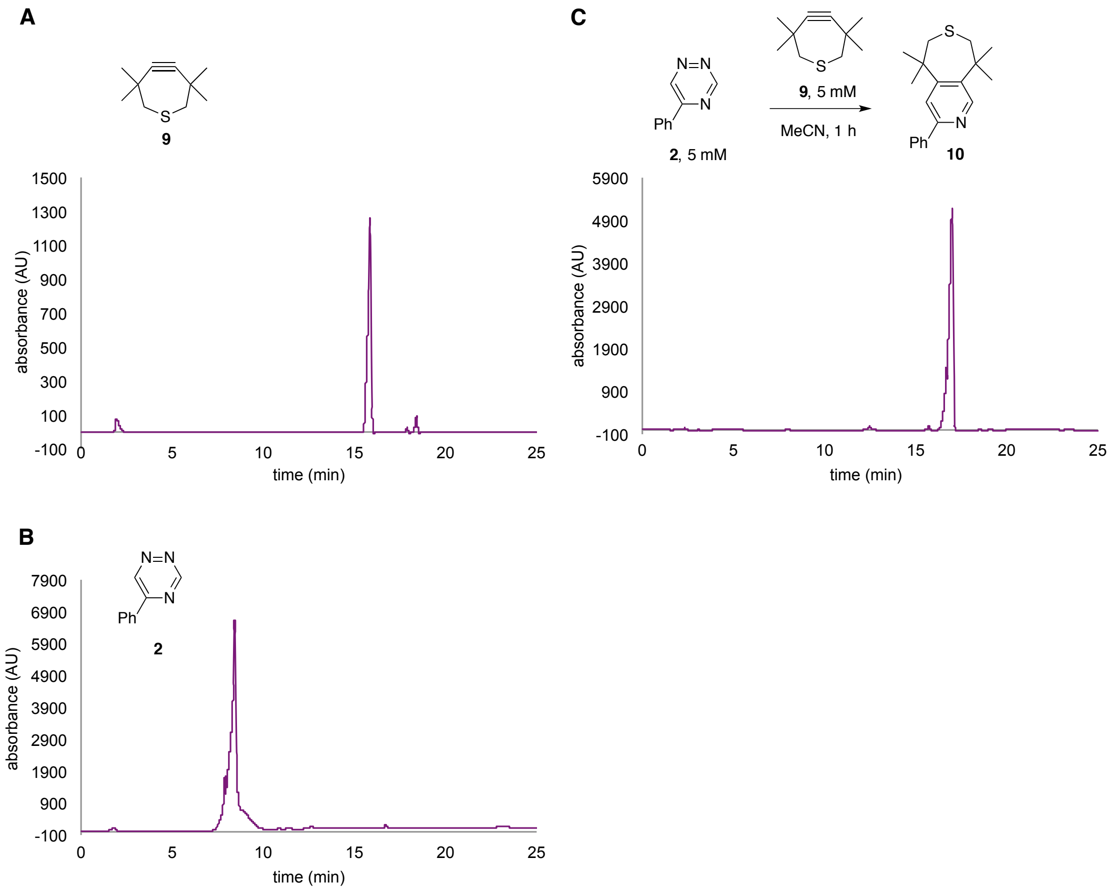

Fig S11. Triazine **2** reacts quantitatively with TMTH **9**. (A) HPLC trace of starting TMTH **9** reagent. (B) HPLC trace of starting triazine **2**. (C) Triazine **2** (5mM in MeCN) was reacted with TMTH **9** (5 mM in MeCN) for 1 h, and monitored by HPLC. All traces were acquired at 210 nm.

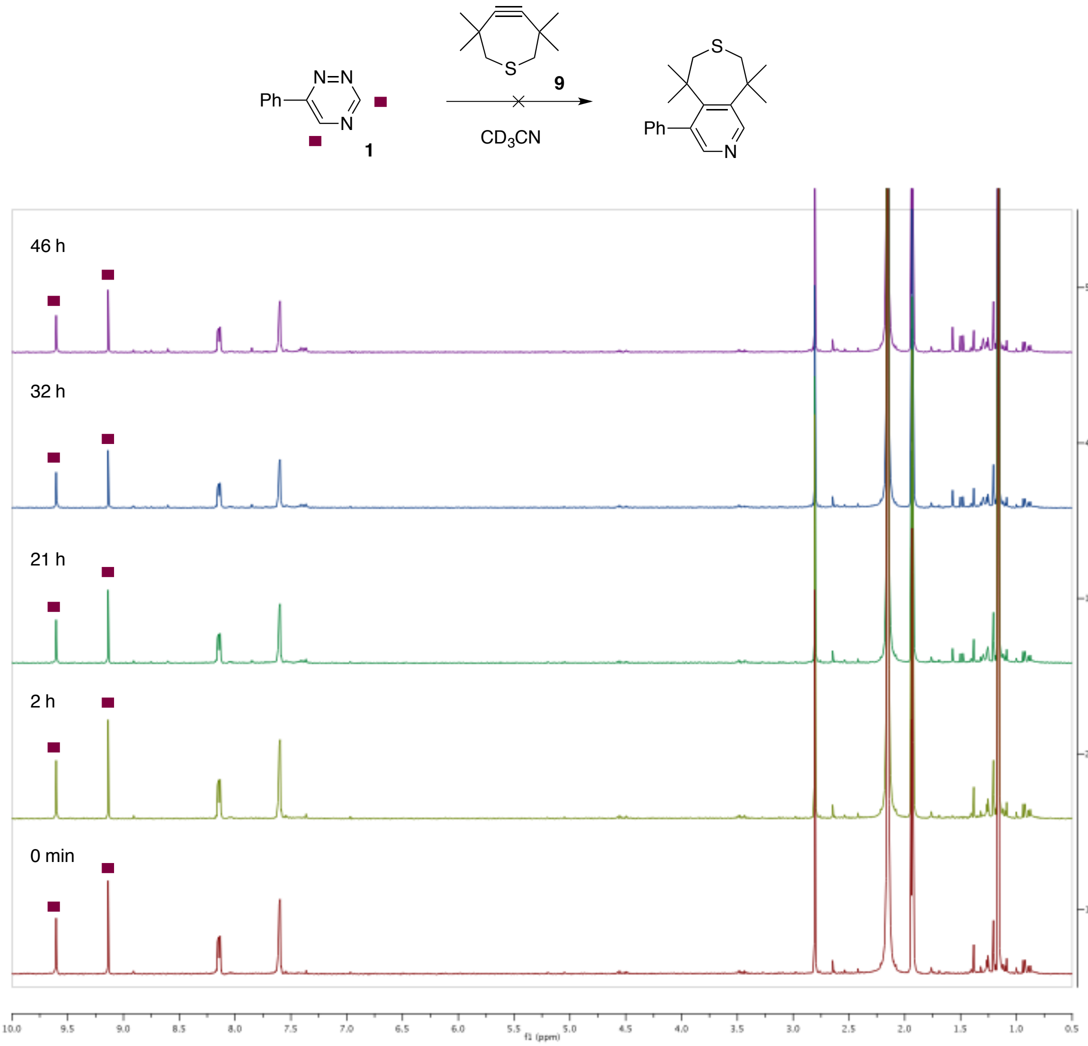

Figure S12. Triazine **1** is orthogonal to TMTH **9**. Triazine **1** (0.24 mL of a 25 mM solution in CD_3CN) was added a solution of TMTH **9** (0.12 mL of a 50 mM solution in CD_3CN) and diluted with 0.24 mL CD_3CN. The reaction was monitored over time by ^{1}H-NMR spectroscopy.

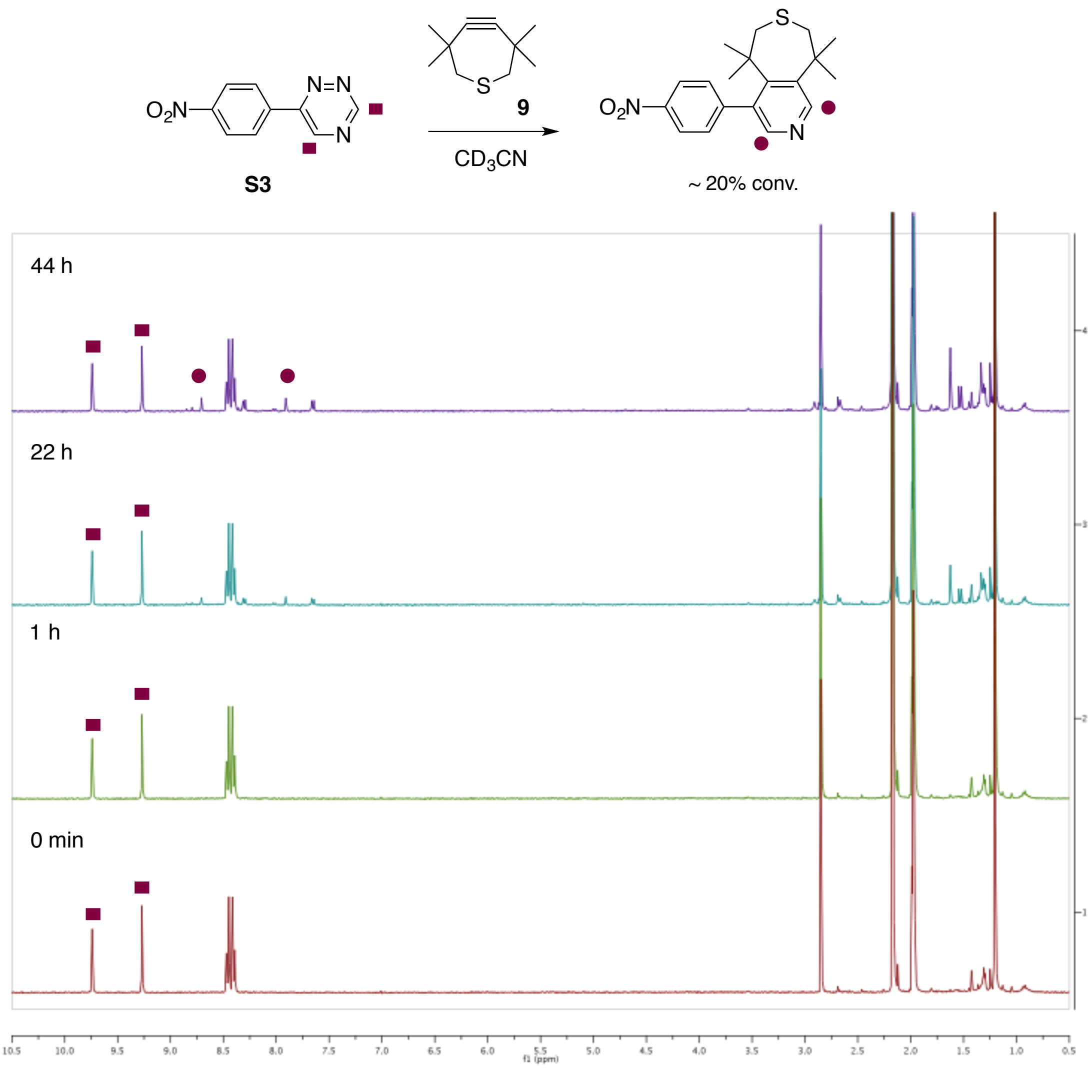

Figure S13. Minimum reactivity is observed between triazine **S3** and TMTH **9**. Triazine **S3** (0.30 mL of a 20 mM solution in CD_3CN) was added a solution of TMTH **9** (0.12 mL of a 50 mM solution in CD_3CN) and diluted with 0.18 mL CD_3CN. The reaction was monitored over time by ^{1}H-NMR spectroscopy.

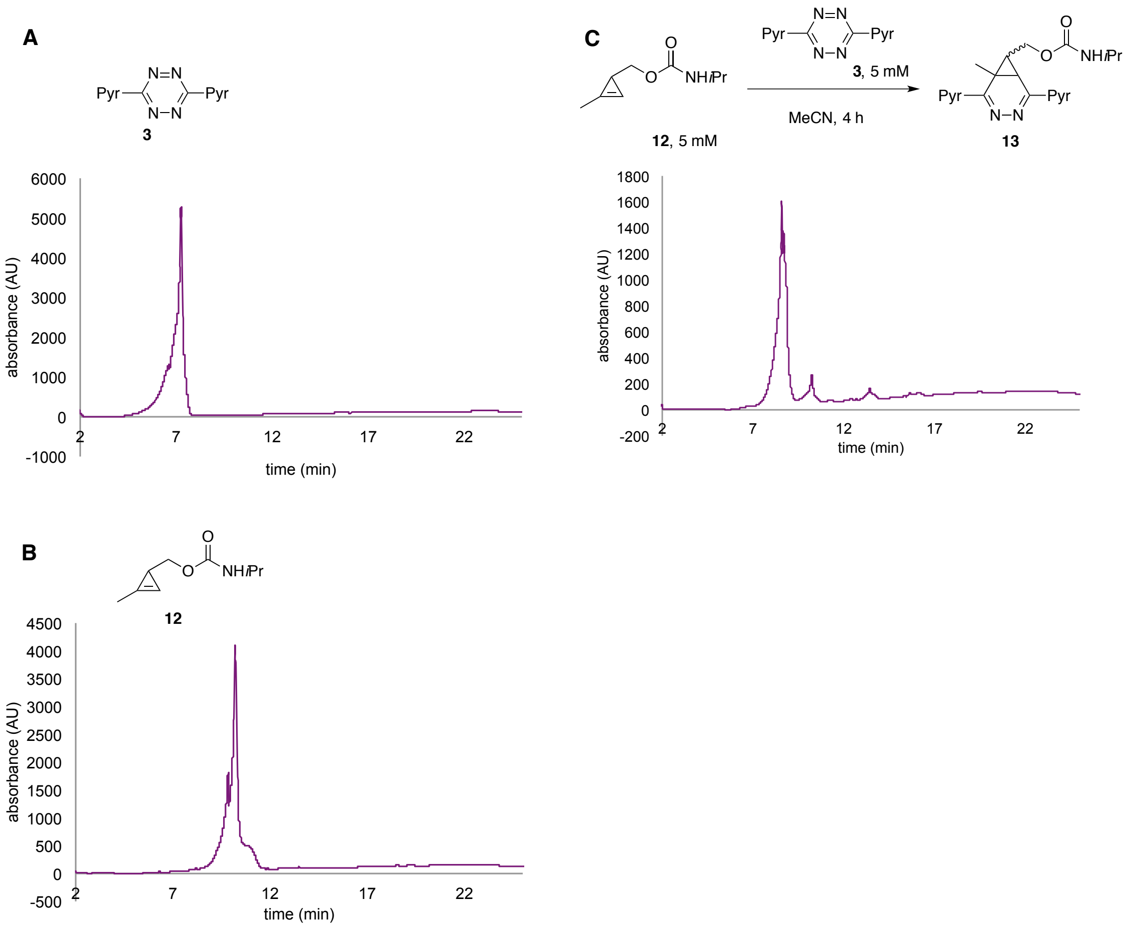

Figure S14. Tetrazine **3** reacts quantitatively with 1,3-disubstituted cyclopropene **12**. (A) HPLC trace of starting tetrazine **3** reagent. (B) HPLC trace of starting cyclopropene **12**. (C) Tetrazine **3** (5 mM in MeCN) was reacted with 1,3-disubstituted cyclopropene **12** (5 mM in MeCN) for 4 h, and monitored by HPLC. The initial cycloadduct formed between **3** and **12** can undergo further rearrangement. HPLC traces were monitored at 210 nm.

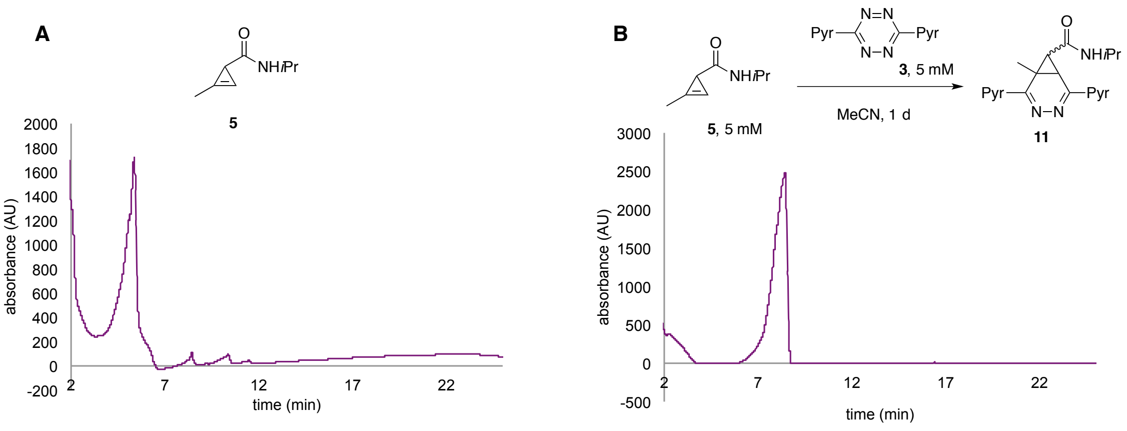

Figure S15. Tetrazine **3** reacts quantitatively with 1,3-disubstituted cyclopropene. (A) HPLC trace of starting cyclopropene **5** trace. (B) Tetrazine **3** (5 mM in MeCN) was reacted with cyclopropene **5** (5 mM in MeCN) for 4 h, and monitored by HPLC. The initial cycloadduct formed between **5** and **3** can undergo further rearrangement. HPLC traces were monitored at 210 nm.

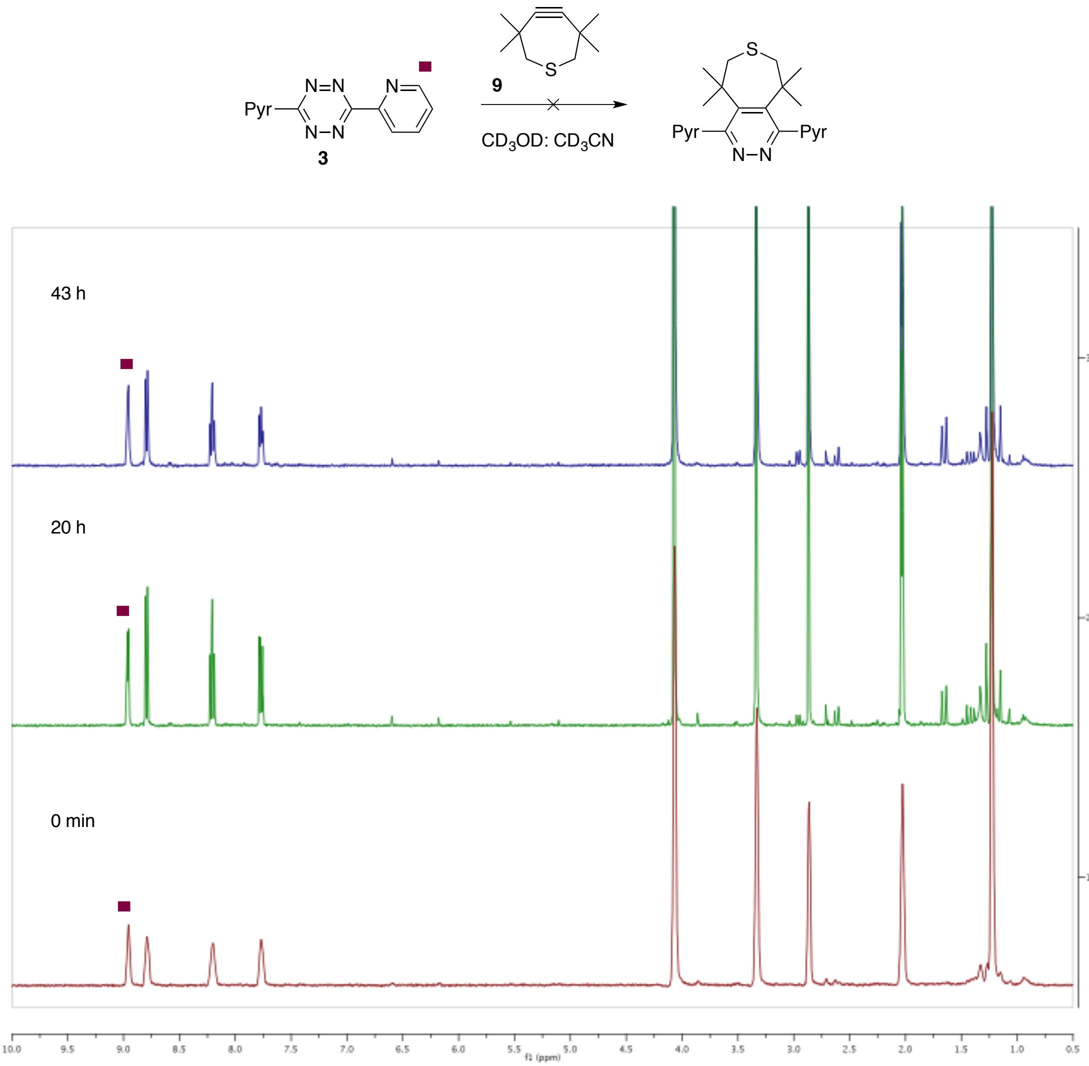

Figure S16. Tetrazine **3** is orthogonal to TMTH **9**. Tetrazine **3** (0.30 mL of a 20 mM solution in CD_3OD) was added a solution of TMTH **9** (0.12 mL of a 50 mM solution in CD_3CN) and diluted with 0.18 mL CD_3CN. The reaction was monitored over time by ^{1}H-NMR spectroscopy.

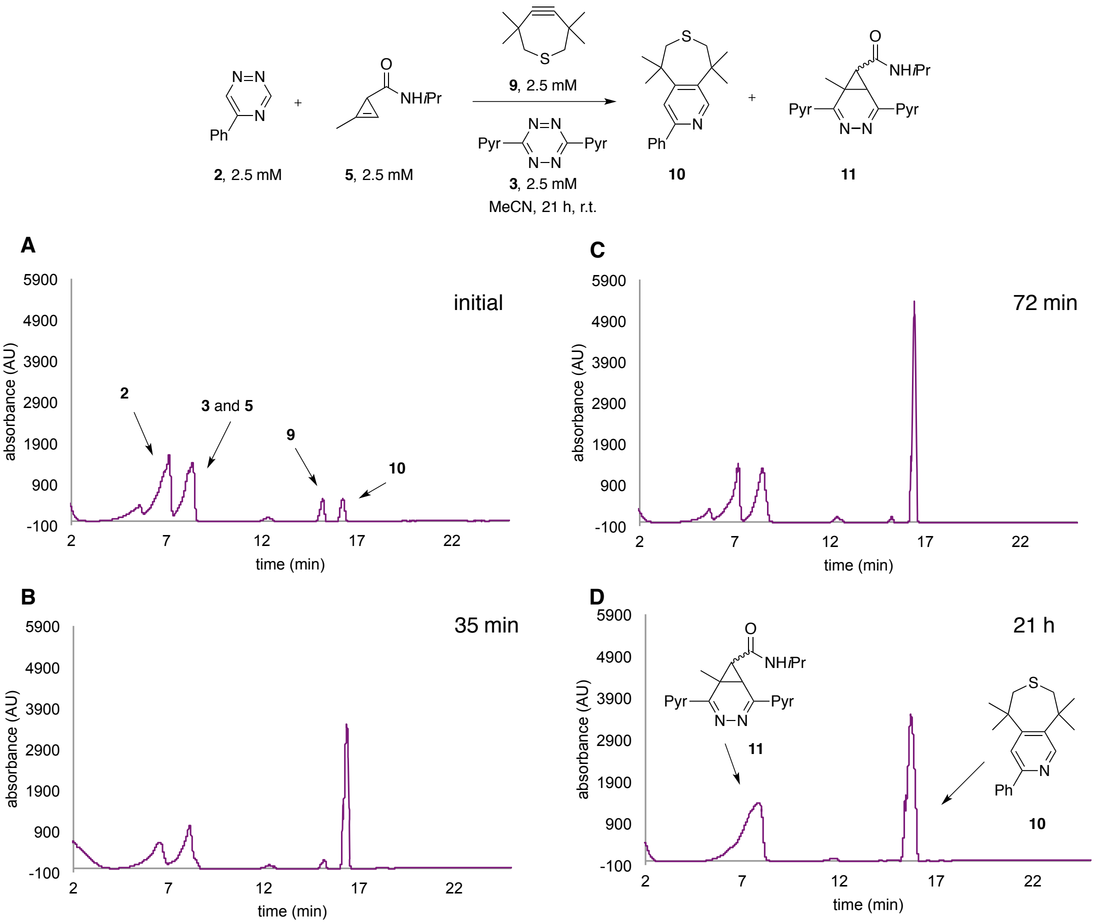

Figure S17. One-pot reaction between triazine **2**, cyclopropene **5**, TMTH **9**, and tetrazine **3**. All four reagents were added together (2.5 mM reach), and the reaction was monitored by HPLC for 1 d (panels A-D). HPLC traces were acquired at 210 nm.

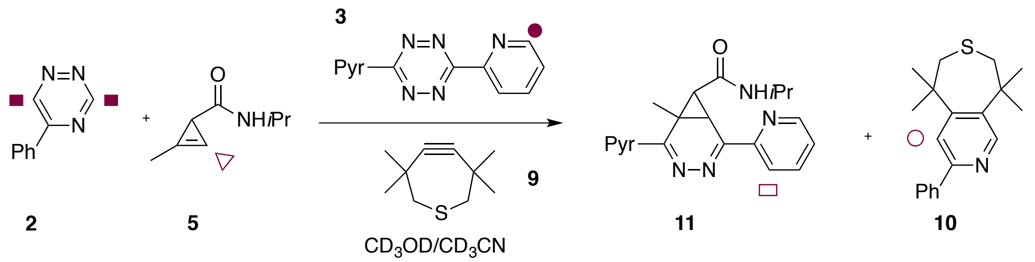

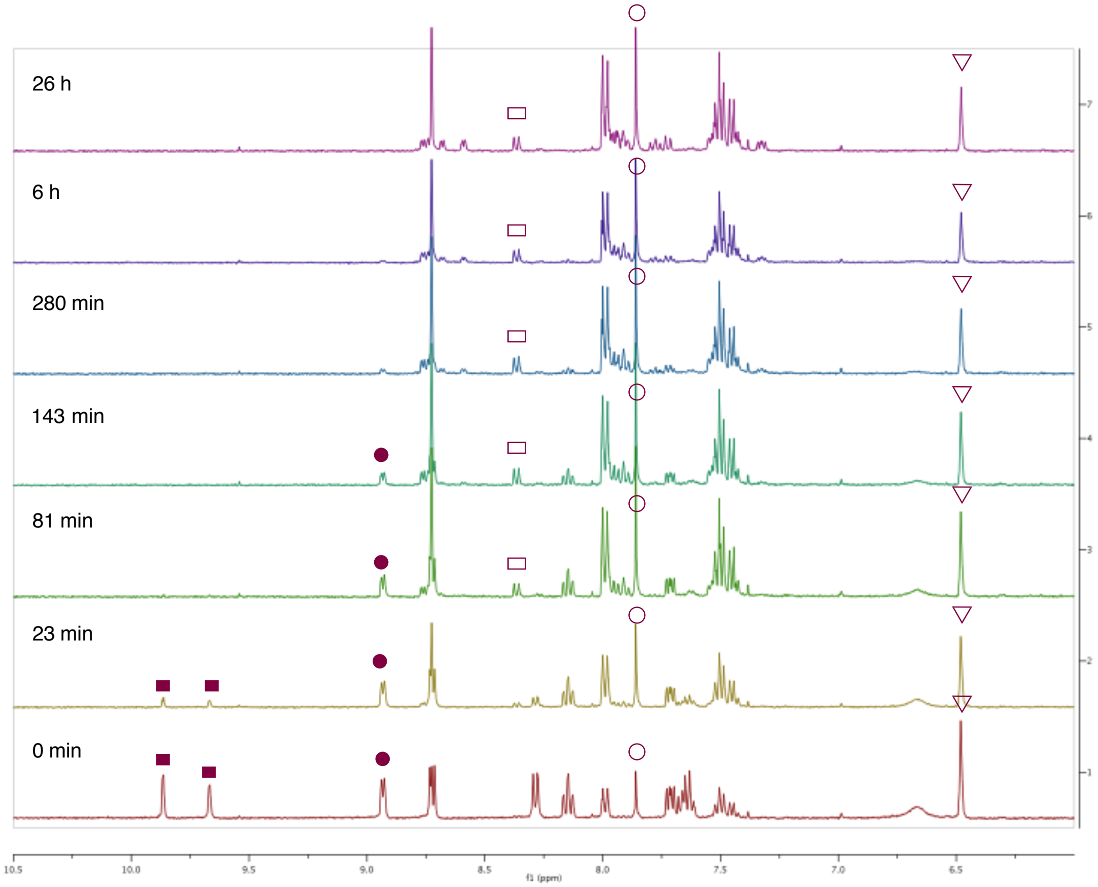

Figure S18. Triazine **2** (0.30 mL of a 25 mM solution in CD_3CN), cyclopropene **5** (0.30 mL of a 35 mM solution in CD_3CN), tetrazine **3** (0.30 mL of a 20 mM solution in CD_3OD), and TMTH **9** (0.30 mL of a 50 mM solution in CD_3CN) were mixed together. The reaction was monitored over time by ^{1}H-NMR spectroscopy.

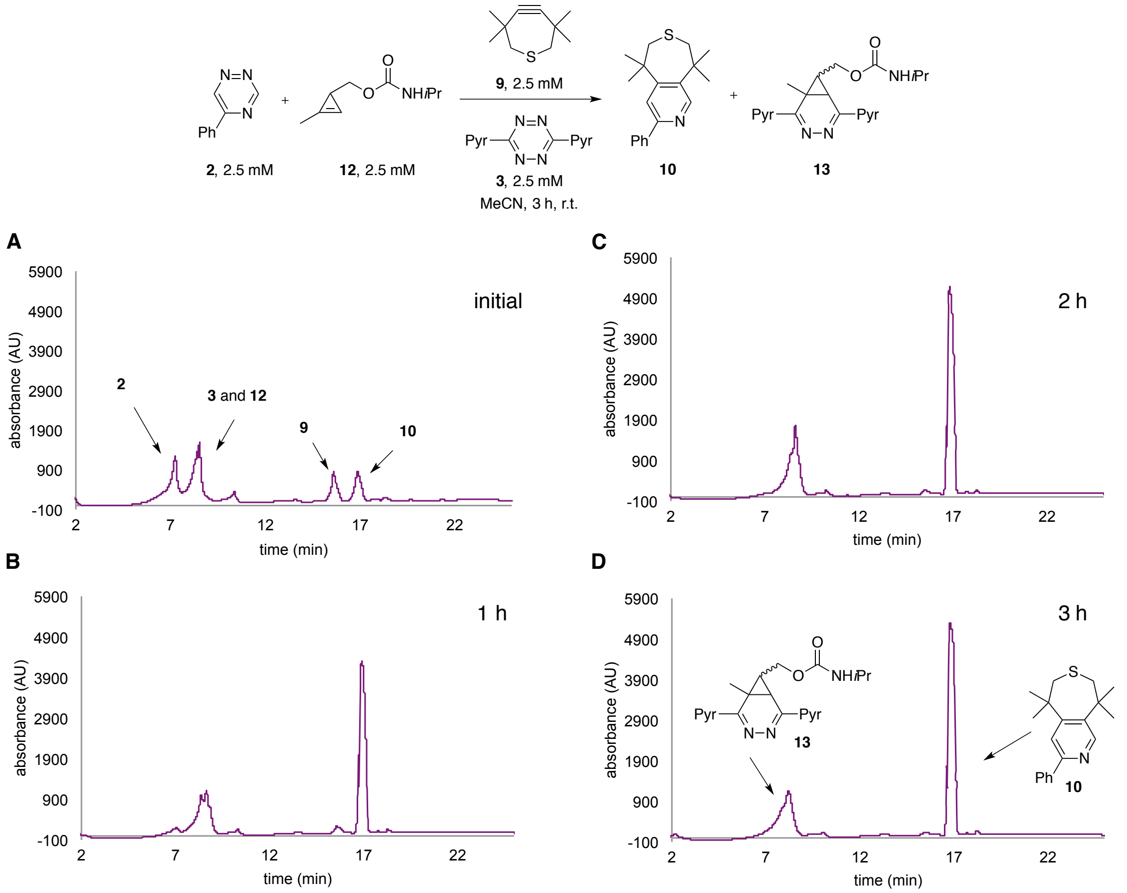

Figure S19. One-pot reaction with triazine **2**, cyclopropene **12**, tetrazine **3**, and TMTH **9**. All four reagents were combined (2.5 mM each in MeCN), and the reaction was monitored by HPLC (210 nm) over 3 h (panels A-D).

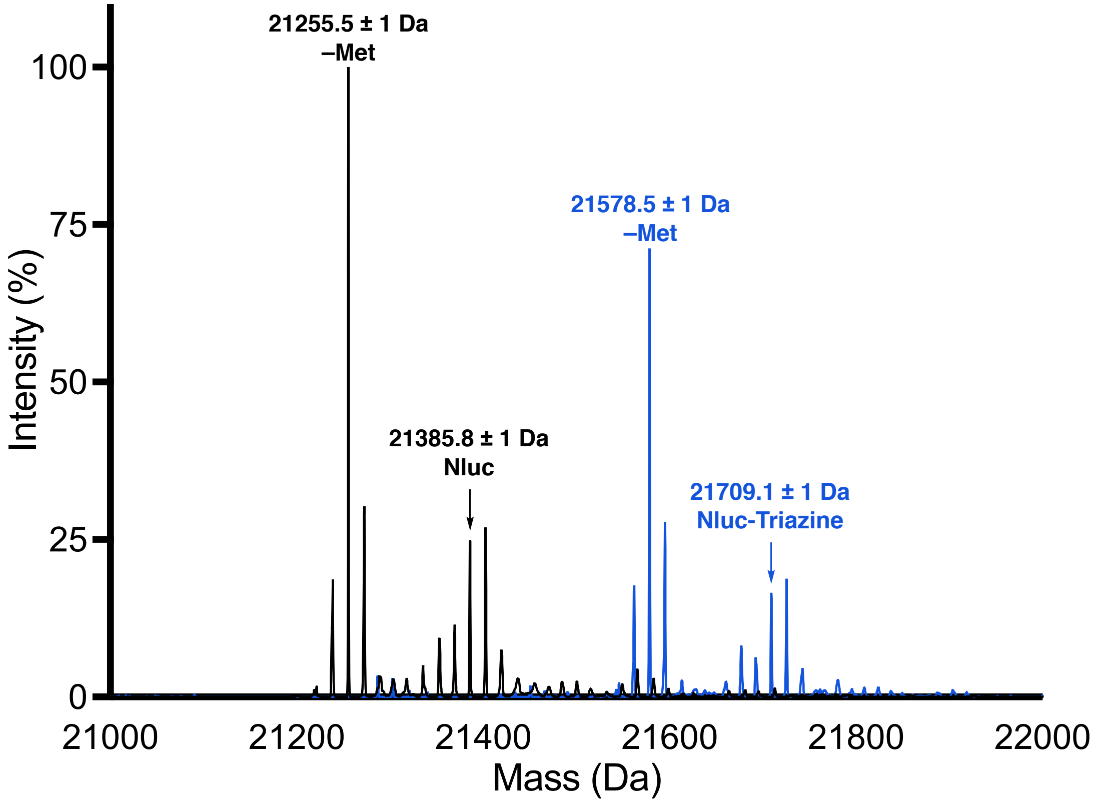

Figure S20. ESI-MS analysis of unlabeled **Nluc** (G180C, black) and **Nluc-Triazine** (blue). The expected masses were observed, in addition to masses corresponding to proteins with oxidized methionine residues and/or N-terminal methionine deletions. The observed and expected mass values for each species are tabulated below.

Species	Expected Mass (Da)	Observed Mass (Da)
Nluc – Met + 2 Met[O]	21239.3	21238.7±1
Nluc – Met + 3 Met[O]	21255.3	21255.5±1
Nluc – Met + 4 Met[O]	21271.3	21272.5±1
Nluc + 1 Met[O]	21354.5	21353.2±1
Nluc + 2 Met[O]	21370.5	21369.5±1
Nluc + 3 Met[O]	21386.5	21385.8±1
Nluc + 4 Met[O]	21402.5	21402.6±1
Nluc + 5 Met[O]	21418.5	21419.6±1
Nluc-Triazine – Met + 2 Met[O]	21562.4	21561.8±1
Nluc-Triazine – Met + 3 Met[O]	21578.4	21578.5±1
Nluc-Triazine – Met + 4 Met[O]	21594.4	21595.0±1
Nluc-Triazine + 1 Met[O]	21677.6	21676.9±1
Nluc-Triazine + 2 Met[O]	21693.6	21692.3±1
Nluc-Triazine + 3 Met[O]	21709.6	21709.1±1
Nluc-Triazine + 4 Met[O]	21725.6	21725.5±1
Nluc-Triazine + 5 Met[O]	21741.6	21742.5±1

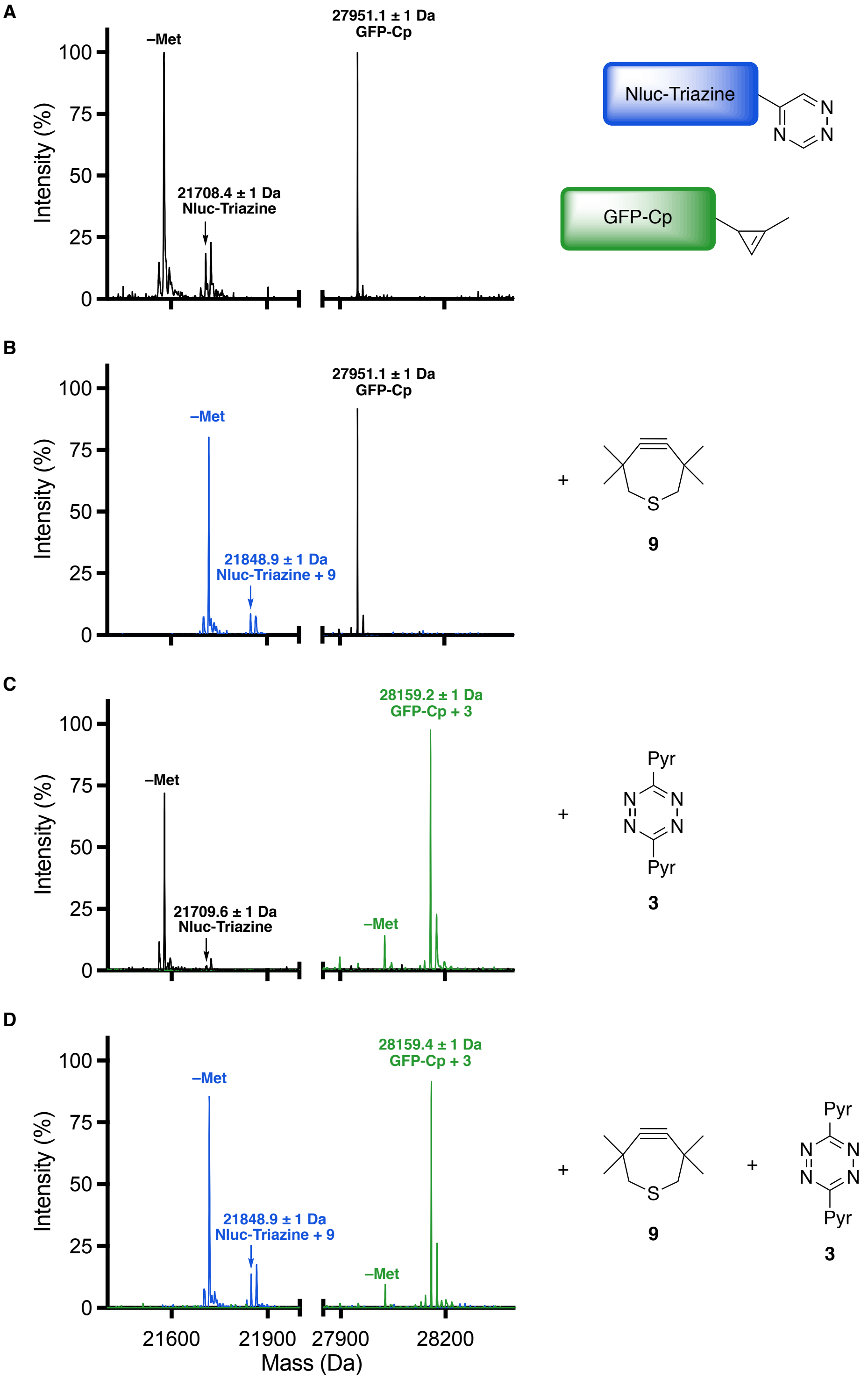

Figure S21. ESI-MS of orthogonal [4+2] cycloadditions on proteins. (A) Masses of starting proteins before exposure to small molecule probes (black). (B) **Nluc-Triazine** reacts upon treatment with TMTH **9** (blue) with a peak at 21848.9±1 Da, which corresponds to the cycloadduct plus three oxidized methionine residues (expected mass 21849.7 Da). A secondary peak at 21718.0±1 Da corresponds to the cycloadduct, plus three oxidized methionine residues, and cleavage of the N-terminal methionine residue

(expected mass 21718.5 Da). **GFP-Cp** remains unreacted (expected mass 27951.4 Da). (C) **GFP-Cp** reacts upon treatment with tetrazine **3** (green) with a major peak at 28159.2±1 Da (expected mass 28159.5 Da). A secondary peak at 28027.7±1 Da corresponds to loss of the N-terminal methionine residue (expected mass 28028.3 Da). **Nluc-Triazine** is unreacted. (D) Addition of both small molecules leads to quantitative conversion to two distinct cycloadducts. No cross-reactivity adducts are observed.

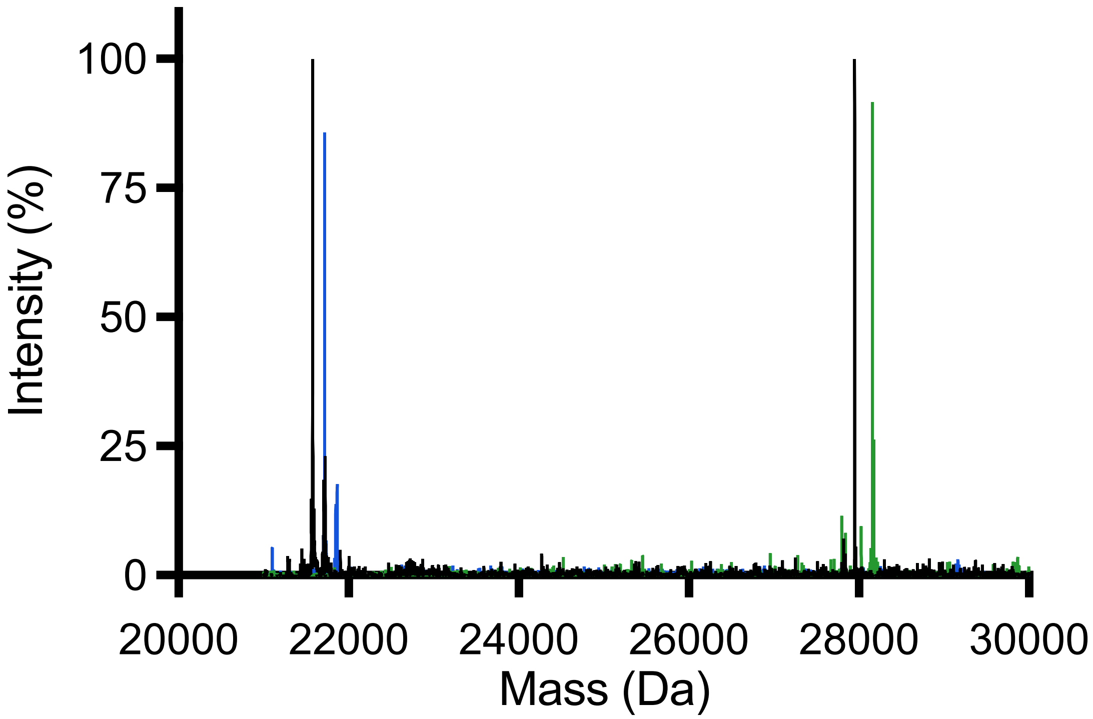

Figure S22. Full ESI-MS spectrum of the one-pot labeling reactions using orthogonal [4+2] cycloadditions. **Nluc-Triazine** and **GFP-Cp** (black) are converted to their respective cycloadducts without cross-reactivity (blue for **Nluc-Triazine**, green for **GFP-Cp**).

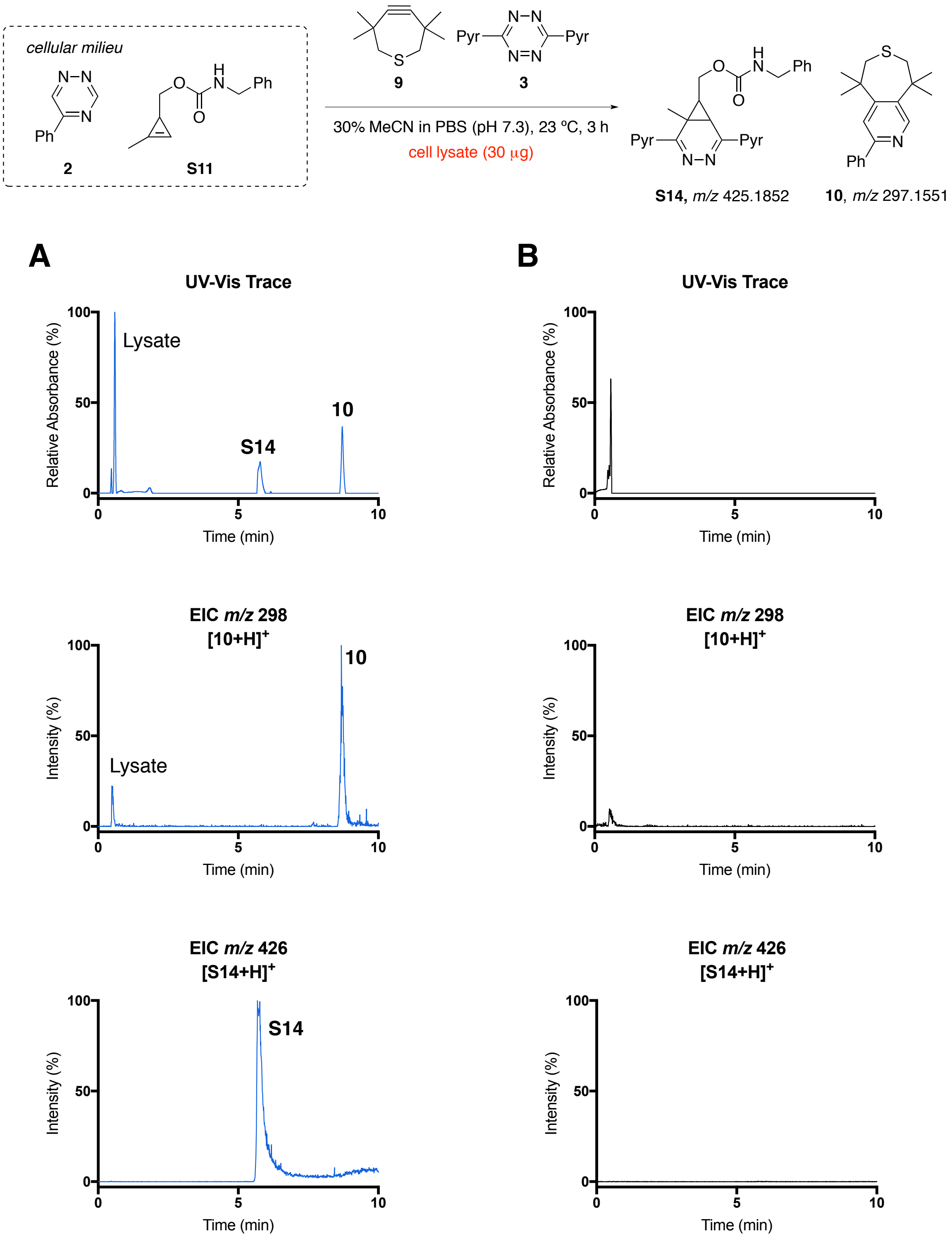

Figure S23. Orthogonal [4+2] cycloadditions were performed in cell lysate. (A) Triazine **2**, Cp **S11**, tetrazine **3** and TMTH **9** were converted to their respective cycloadducts in the presence of cell lysate (blue). (B) No adducts were observed in the absence of the requisite reaction partners (black). The reactions were analyzed by absorbance (top) and extracted ion chromatographs (middle, *m/z* 298; bottom, *m/z* 426).

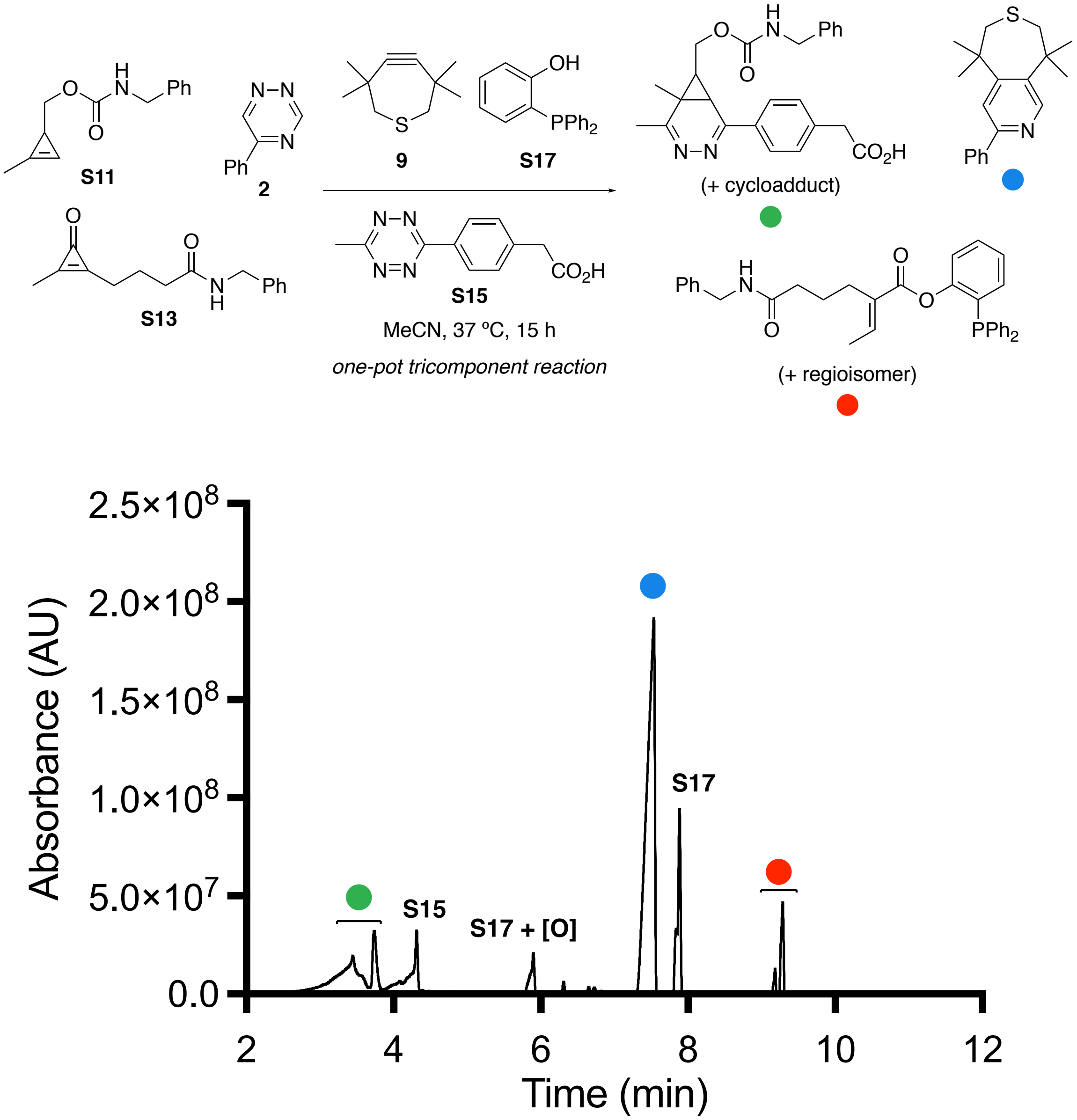

Figure S24. Tricomponent, one-pot bioorthogonal reaction. Triazine **2**, Cp **S11**, cyclopropenone **S13**, TMTH **9**, tetrazine **S15**, and phosphine **S17** were mixed at equimolar concentrations in MeCN (1 mM final concentration for all reagents). The reaction was monitored by LC-MS. Three distinct ligation products were observed, corresponding to the matched reaction partners. No cross-reactivity was observed. Individual traces and reference reactions are shown in Fig. S25 below.

A

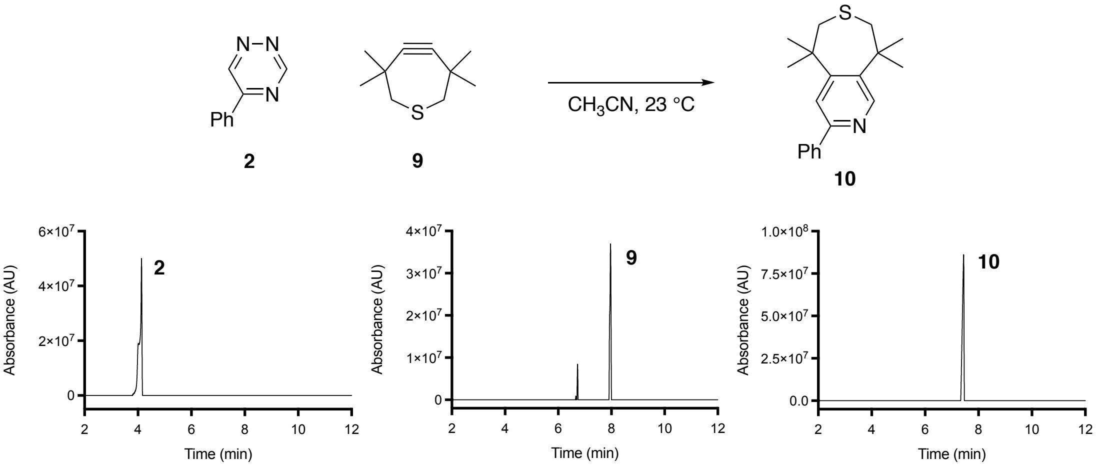

N–N
N
Ph
S
CH$_3$CN, 23 °C
2
9
10
2
9
10
Absorbance (AU)
Time (min)

B

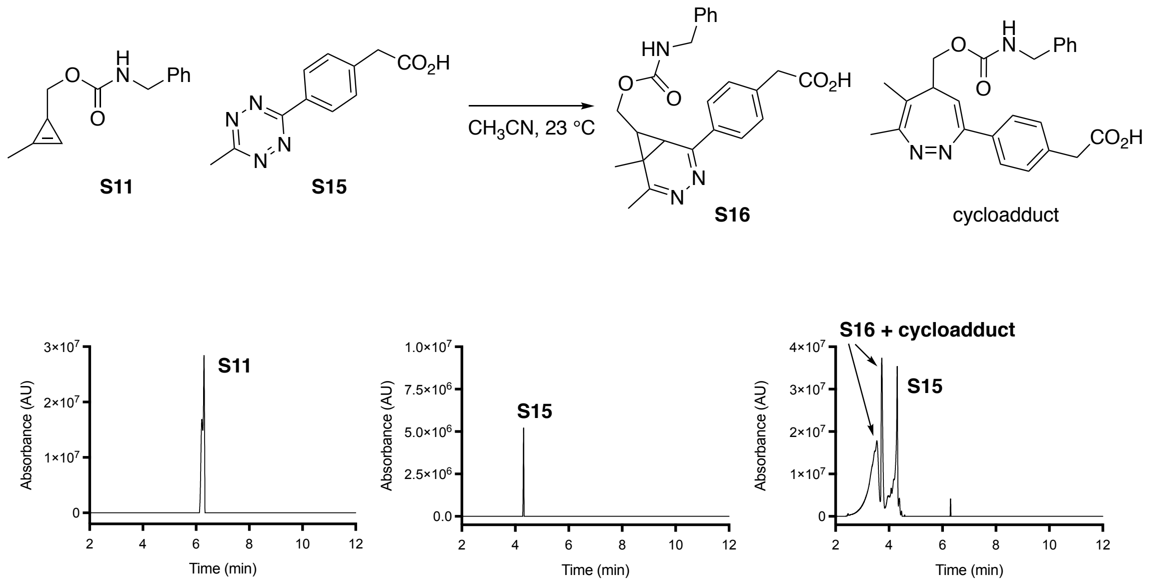

H
O
N
Ph
O
CO$_2$H
N
N
HN
Ph
CH$_3$CN, 23 °C
S11
S15
S16
cycloadduct
S11
S15
S16 + cycloadduct
S15
Absorbance (AU)
Time (min)

C

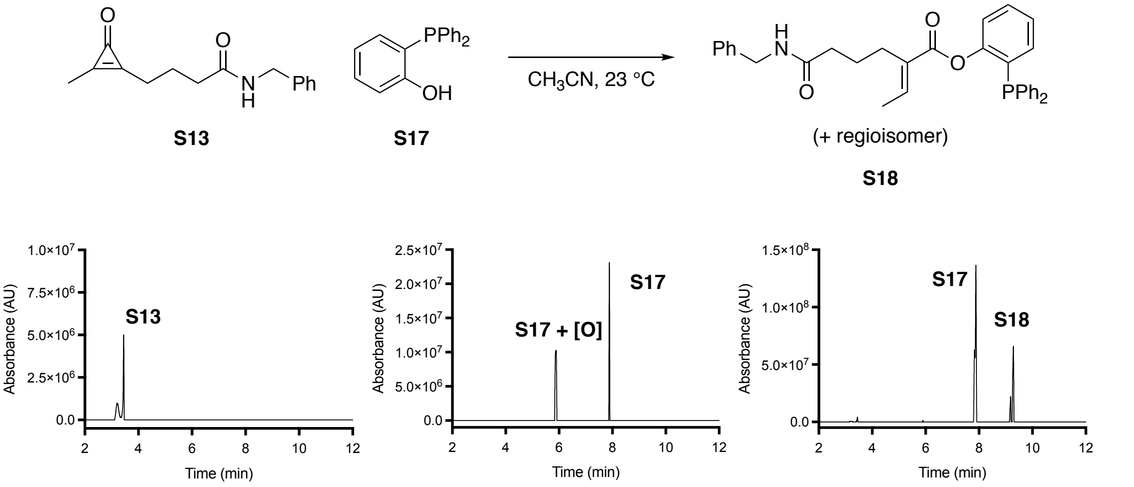

O
PPh$_2$
OH
N
H
Ph
CH$_3$CN, 23 °C
PPh$_2$
S13
S17
(+ regioisomer)
S18
S13
S17 + [O]
S17
S17
S18
Absorbance (AU)
Time (min)

Figure S25. LC-MS traces of starting bioorthogonal reagents and corresponding ligated adducts. (A) Triazine **2** and TMTH **9** were mixed in a 1:1 ratio (1 mM final concentration) in MeCN, then the reaction was monitored by LC-MS for the formation of ligation adduct **10**. The reaction trace is shown on the far right. The first two traces correspond to starting materials only. (B) Cp **S11** and tetrazine **S15** were mixed in a 1:1 ratio (1 mM final concentration) in MeCN, then the reaction was monitored by LC-MS for the formation of ligation adduct **S16**. **S16** can further rearrange over the course of the experiment.[20] The reaction trace is shown on the far right. The first two traces correspond to starting materials only. (C) Cyclopropenone **S13** and phosphine **S17** were mixed in a 1:1 ratio (1 mM final concentration) in MeCN, then the reaction was monitored by LC-MS for the formation of ligation adduct **S18** and its regioisomer. Phosphine **S17** oxidized over the course of the reaction (**S17 + [O]**). The reaction trace is shown on the far right. The first two traces correspond to starting materials only.

1H spectrum

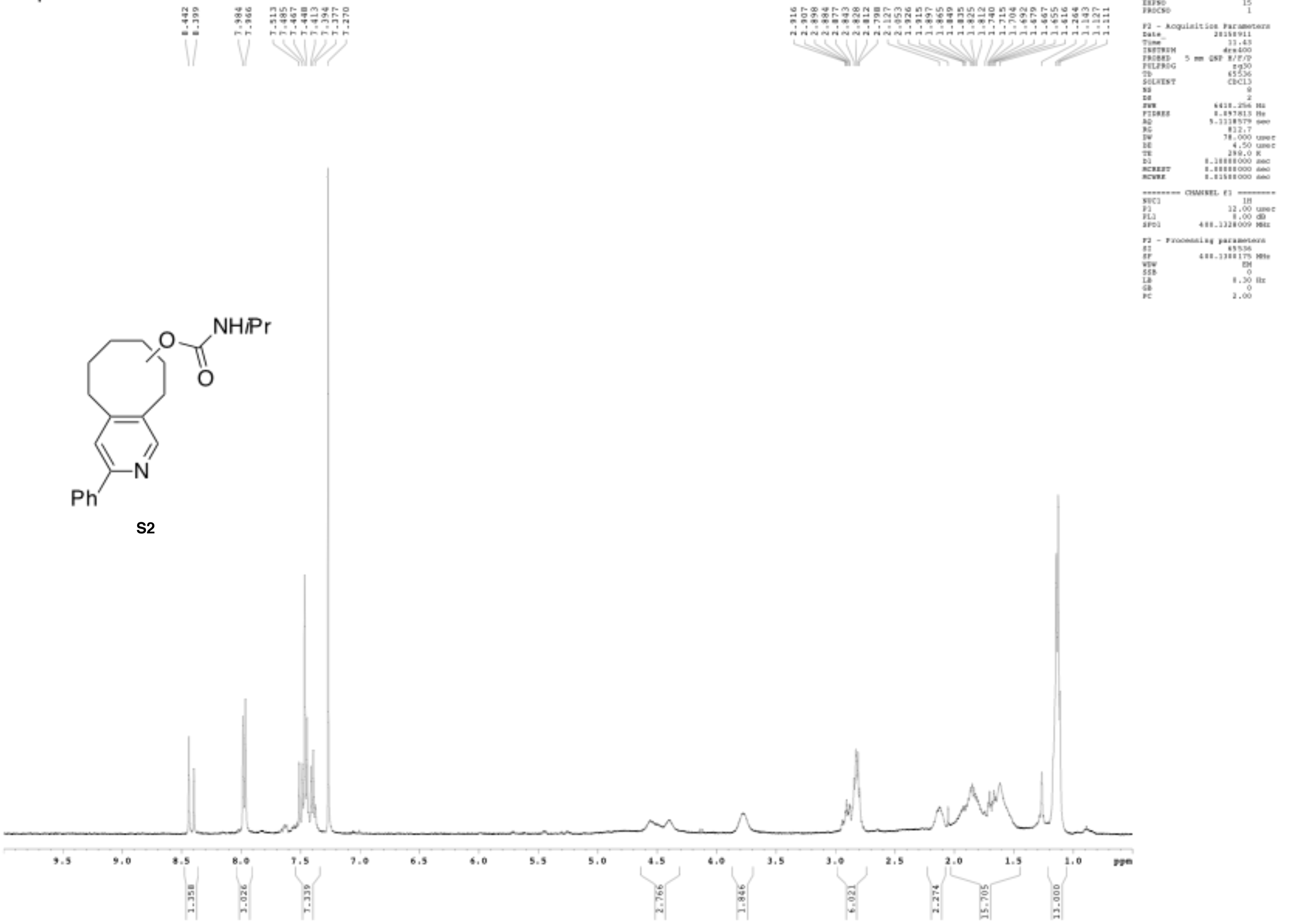

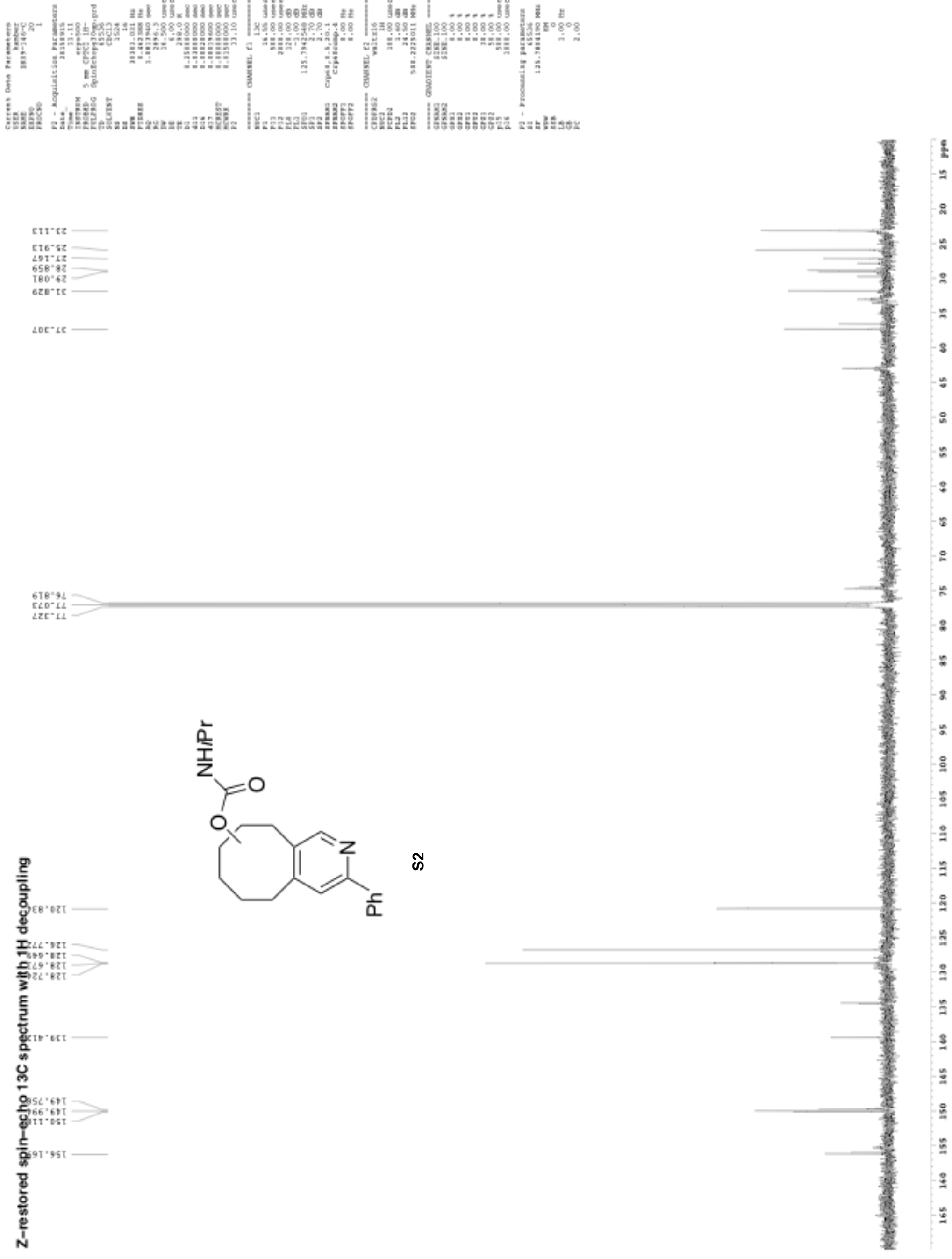
Z-restored spin-echo 13C spectrum with 1H decoupling
NH*i*Pr
O
N
Ph
S2
ppm

1H spectrum

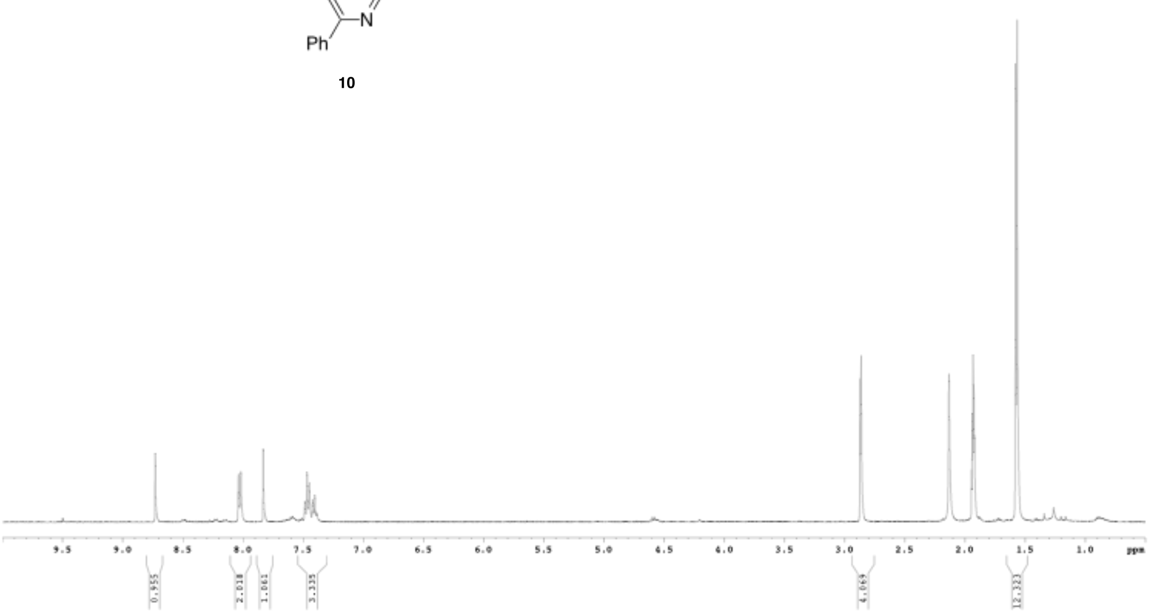

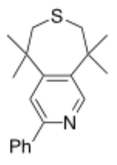

10

Z–restored spin–echo 13C spectrum with 1H decoupling

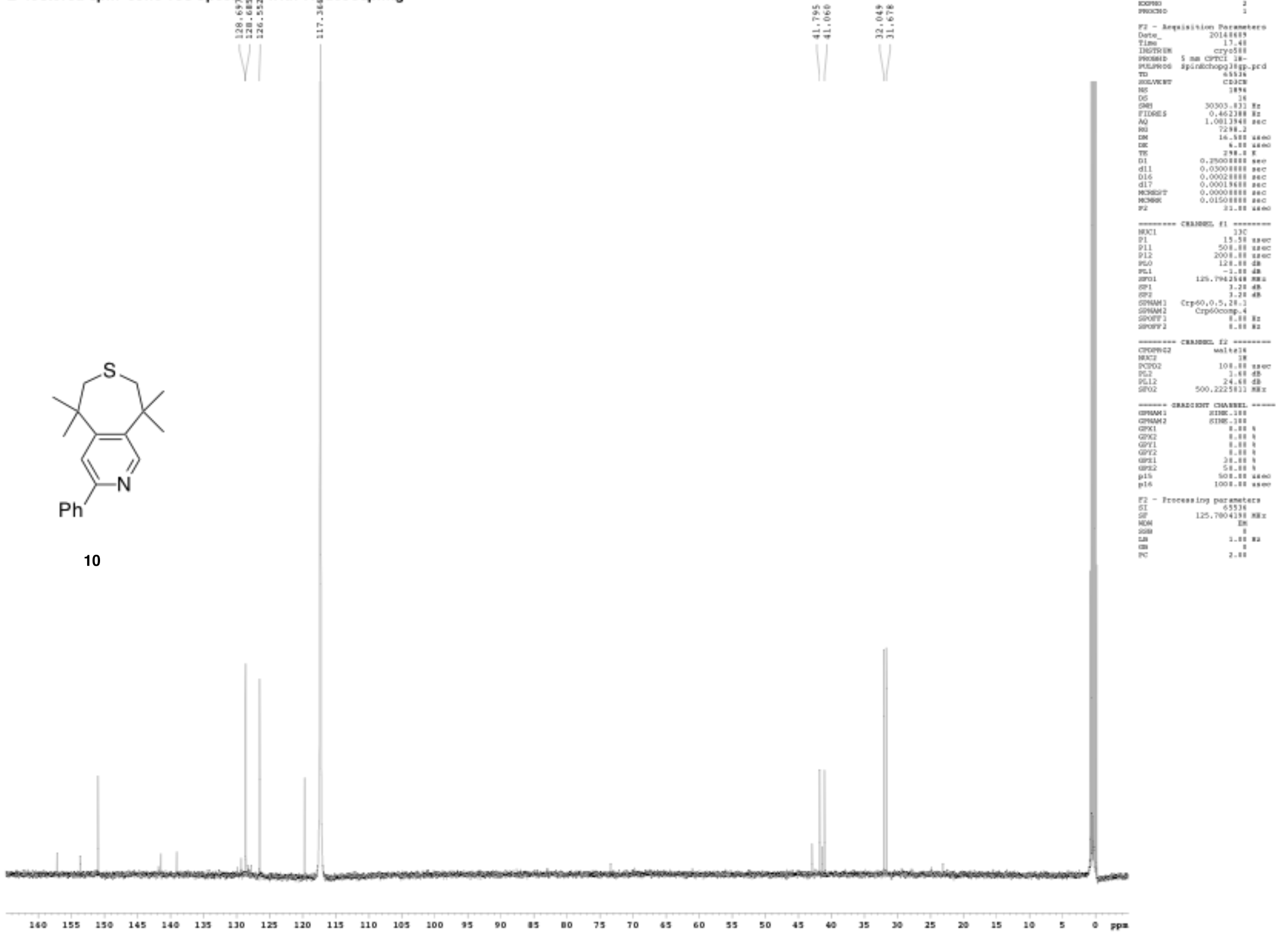

10

1H spectrum

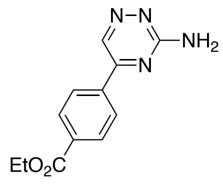

S5

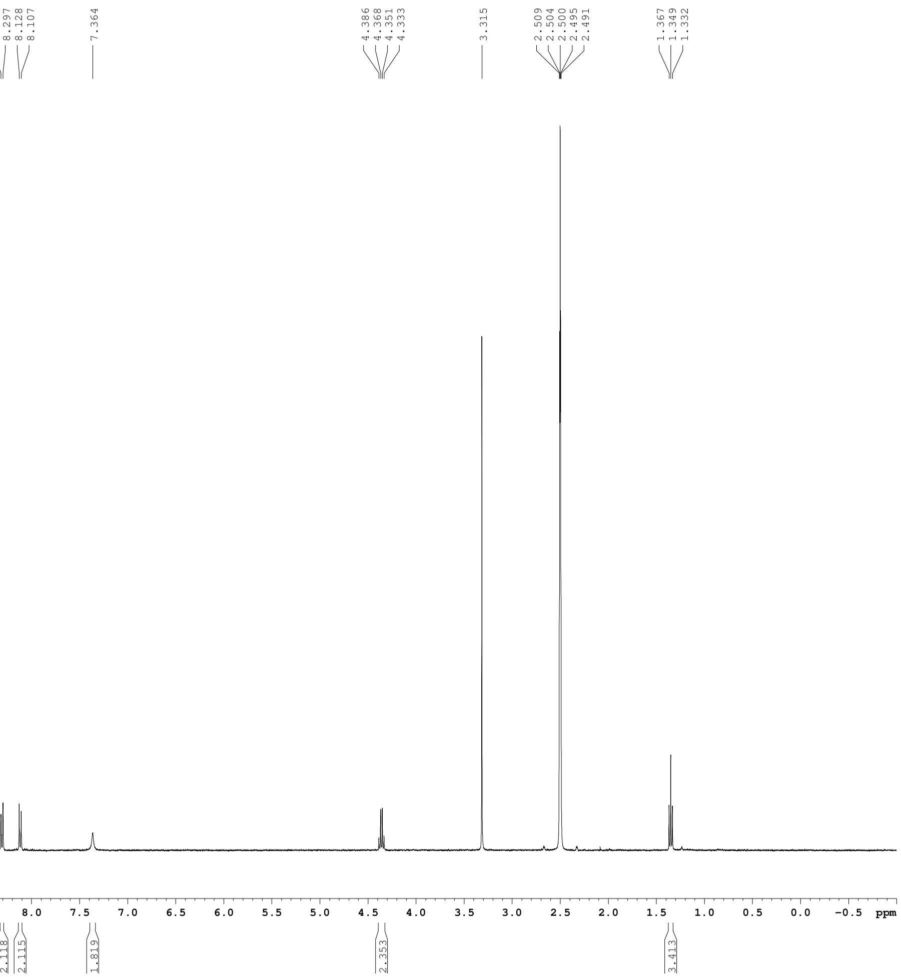


```
Current Data Parameters
NAME             SN3-098
EXPNO                  1
PROCNO                 1

F2 - Acquisition Parameters
Date_           20170630
Time               19.02
INSTRUM           drx400
PROBHD   5 mm Multinucl
PULPROG             zg30
TD                 65536
SOLVENT            CD3OD
NS                     8
DS                     2
SWH             6410.256 Hz
FIDRES          0.097813 Hz
AQ             5.1118078 sec
RG                1824.6
DW                78.000 usec
DE                  4.50 usec
TE                 298.1 K
D1            0.10000000 sec
MCREST   0 sec
MCWRK         0.01500000 sec

======== CHANNEL f1 ========
NUC1                  1H
P1                 12.00 usec
PL1                -1.10 dB
SFO1         400.1328009 MHz

F2 - Processing parameters
SI                 65536
SF           400.1300075 MHz
WDW                   EM
SSB      0
LB                  0.30 Hz
GB       0
PC                  2.00
```

Z-restored spin-echo 13C spectrum with 1H decoupling

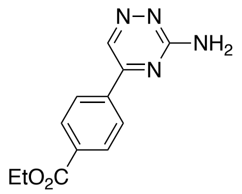

S5

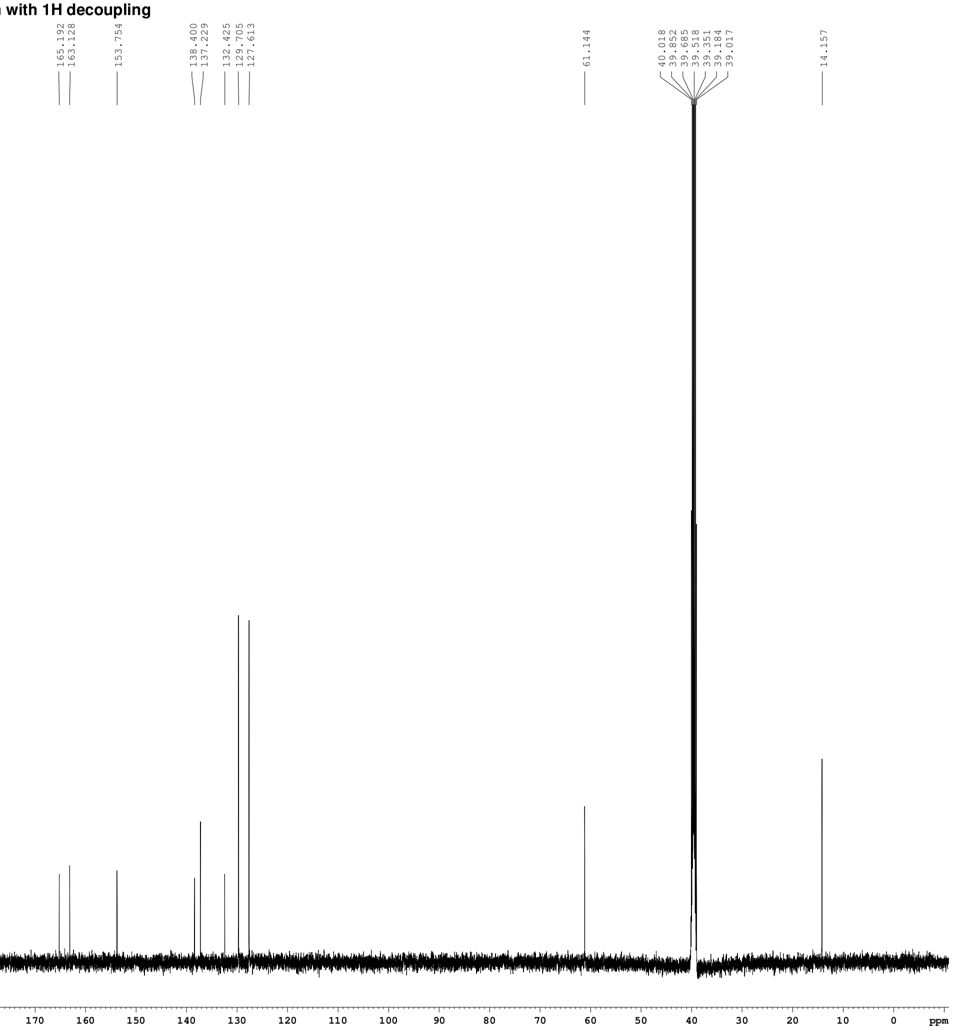


```
Current Data Parameters
NAME              SN3-084
EXPNO                   7
PROCNO                  1

F2 - Acquisition Parameters
Date_            20170612
Time                19.09
INSTRUM           cryo500
PROBHD   5 mm CPTCI 1H-
PULPROG  SpinEchopg30gp.prd
TD                  65536
SOLVENT             CD3OD
NS                    641
DS                     16
SWH             30303.031 Hz
FIDRES           0.462388 Hz
AQ              1.0813440 sec
RG                 7298.2
DW                 16.500 usec
DE                   6.00 usec
TE                  298.0 K
D1             0.25000000 sec
d11            0.03000000 sec
D16            0.00020000 sec
d17            0.00019600 sec
MCREST   0 sec
MCWRK          0.01500000 sec
P2                  33.10 usec

======== CHANNEL f1 ========
NUC1                  13C
P1                  16.55 usec
P11                500.00 usec
P12               2000.00 usec
PL0                120.00 dB
PL1                 -1.00 dB
SFO1          125.7942548 MHz
SP1                  2.70 dB
SP2                  2.70 dB
SPNAM[1] Crp60,0.5,20.1
SPNAM[2]    Crp60comp.4
SPOFF1   0 Hz
SPOFF2   0 Hz

======== CHANNEL f2 ========
CPDPRG[2          waltz16
NUC2                   1H
PCPD2              100.00 usec
PL2                  1.60 dB
PL12                24.50 dB
SFO2          500.2225011 MHz

====== GRADIENT CHANNEL =====
GPNAM[1]         SINE.100
GPNAM[2]         SINE.100
GPX1     0 %
GPX2     0 %
GPY1     0 %
GPY2     0 %
GPZ1                30.00 %
GPZ2                50.00 %
p15                500.00 usec
p16               1000.00 usec

F2 - Processing parameters
SI                  65536
SF            125.7804788 MHz
WDW                    EM
SSB      0
LB                   1.00 Hz
GB       0
PC                   2.00
```

1H spectrum

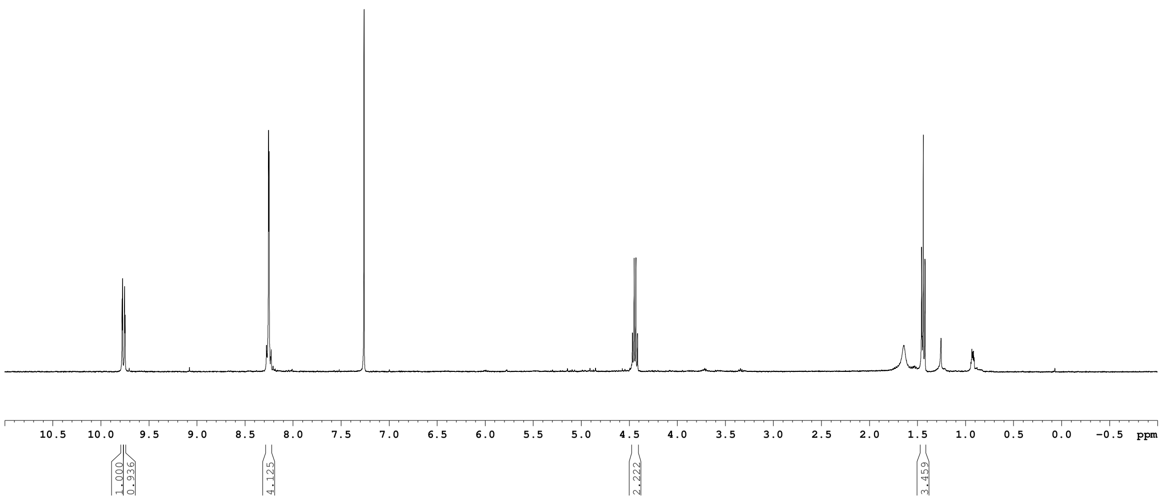

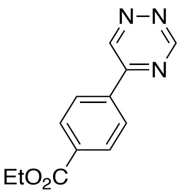

S6

```
Current Data Parameters
NAME              SN3-103
EXPNO                   1
PROCNO                  1

F2 - Acquisition Parameters
Date_            20170703
Time                10.25
INSTRUM            drx400
PROBHD   5 mm Multinucl
PULPROG              zg30
TD                  65536
SOLVENT              DMSO
NS                      8
DS                      2
SWH              6410.256 Hz
FIDRES           0.097813 Hz
AQ              5.1118078 sec
RG                 1290.2
DW                 78.000 usec
DE                   4.50 usec
TE                  298.0 K
D1             0.10000000 sec
MCREST   0 sec
MCWRK          0.01500000 sec

======== CHANNEL f1 ========
NUC1                   1H
P1                  12.00 usec
PL1                 -1.10 dB
SFO1          400.1328009 MHz

F2 - Processing parameters
SI                  65536
SF            400.1300216 MHz
WDW                    EM
SSB      0
LB                   0.30 Hz
GB       0
PC                   2.00
```

Z-restored spin-echo 13C spectrum with 1H decoupling

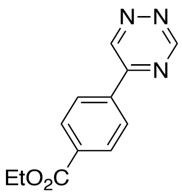

S6

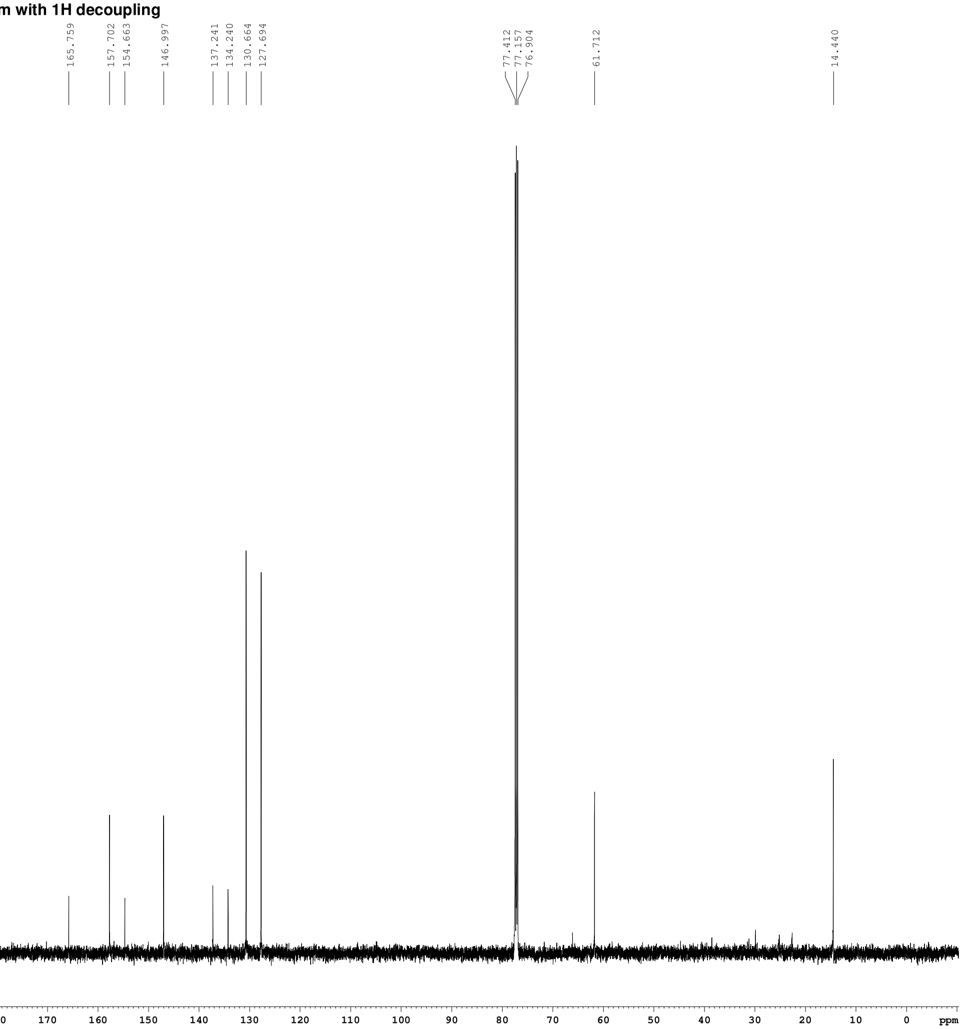


```
Current Data Parameters
NAME            SN3-103
EXPNO                 4
PROCNO                1

F2 - Acquisition Parameters
Date_          20170703
Time              19.17
INSTRUM         cryo500
PROBHD   5 mm CPTCI 1H-
PULPROG  SpinEchopg30gp.prd
TD                65536
SOLVENT            DMSO
NS                  473
DS                   16
SWH           30303.031 Hz
FIDRES         0.462388 Hz
AQ            1.0813440 sec
RG               5792.6
DW               16.500 usec
DE                 6.00 usec
TE                298.0 K
D1           0.25000000 sec
d11          0.03000000 sec
D16          0.00020000 sec
d17          0.00019600 sec
MCREST   0 sec
MCWRK        0.01500000 sec
P2                33.10 usec

======== CHANNEL f1 ========
NUC1                13C
P1                16.55 usec
P11              500.00 usec
P12             2000.00 usec
PL0              120.00 dB
PL1               -1.00 dB
SFO1        125.7942548 MHz
SP1                2.70 dB
SP2                2.70 dB
SPNAM[1] Crp60,0.5,20.1
SPNAM[2]    Crp60comp.4
SPOFF1   0 Hz
SPOFF2   0 Hz

======== CHANNEL f2 ========
CPDPRG[2        waltz16
NUC2                 1H
PCPD2            100.00 usec
PL2                1.60 dB
PL12              24.50 dB
SFO2        500.2225011 MHz

====== GRADIENT CHANNEL =====
GPNAM[1]       SINE.100
GPNAM[2]       SINE.100
GPX1     0 %
GPX2     0 %
GPY1     0 %
GPY2     0 %
GPZ1              30.00 %
GPZ2              50.00 %
p15              500.00 usec
p16             1000.00 usec

F2 - Processing parameters
SI                65536
SF          125.7804064 MHz
WDW                  EM
SSB      0
LB                 1.00 Hz
GB       0
PC                 2.00
```

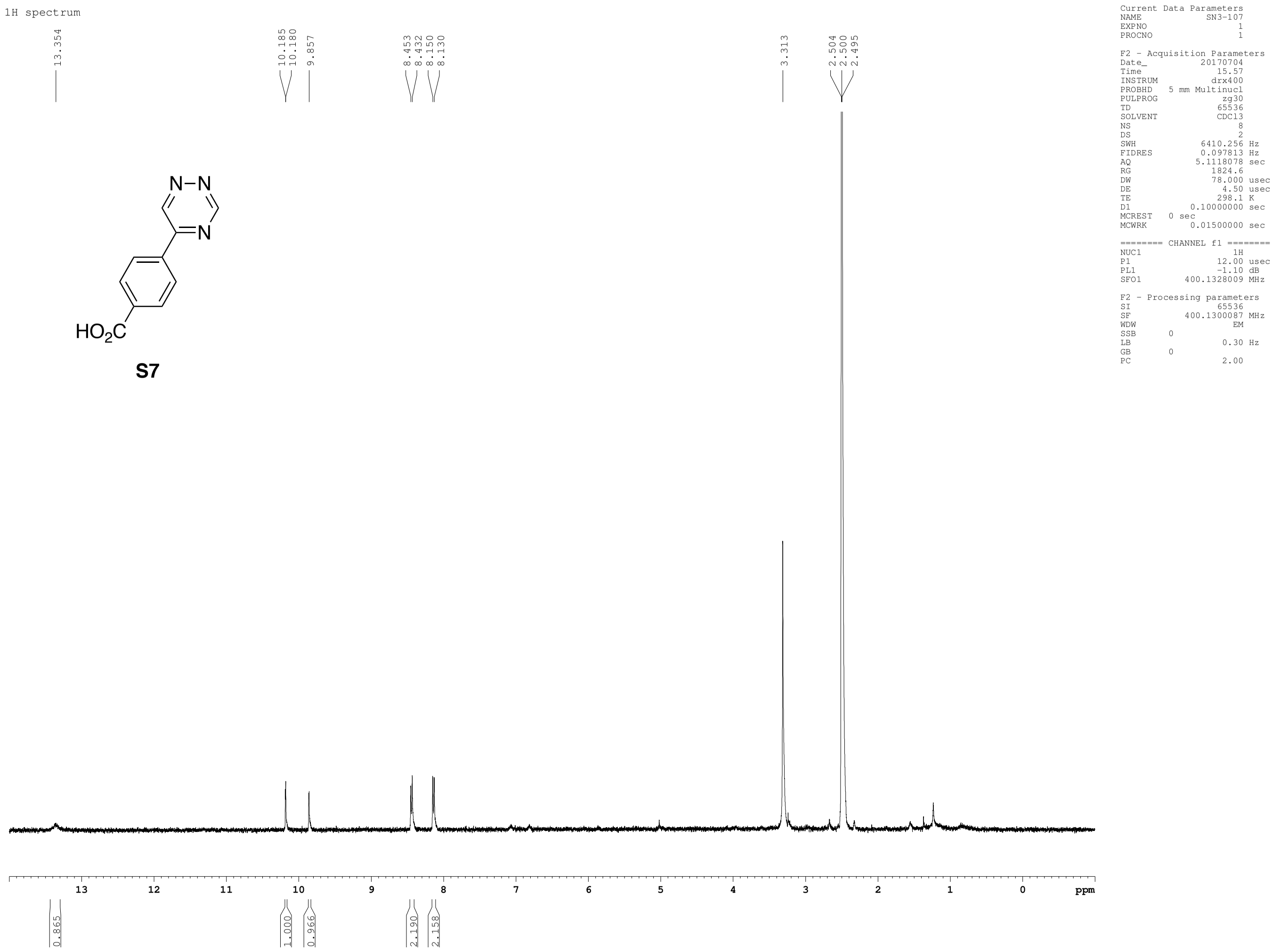
1H spectrum
13.354
10.185
10.180
9.857
8.453
8.432
8.150
8.130
3.313
2.504
2.500
2.495
N–N
N
HO2C
S7
13 12 11 10 9 8 7 6 5 4 3 2 1 0 ppm
0.865
1.000
0.966
2.190
2.158
Current Data Parameters
NAME SN3-107
EXPNO 1
PROCNO 1
F2 - Acquisition Parameters
Date_ 20170704
Time 15.57
INSTRUM drx400
PROBHD 5 mm Multinucl
PULPROG zg30
TD 65536
SOLVENT CDCl3
NS 8
DS 2
SWH 6410.256 Hz
FIDRES 0.097813 Hz
AQ 5.1118078 sec
RG 1824.6
DW 78.000 usec
DE 4.50 usec
TE 298.1 K
D1 0.10000000 sec
MCREST 0 sec
MCWRK 0.01500000 sec
======== CHANNEL f1 ========
NUC1 1H
P1 12.00 usec
PL1 -1.10 dB
SFO1 400.1328009 MHz
F2 - Processing parameters
SI 65536
SF 400.1300087 MHz
WDW EM
SSB 0
LB 0.30 Hz
GB 0
PC 2.00

Z-restored spin-echo 13C spectrum with 1H decoupling

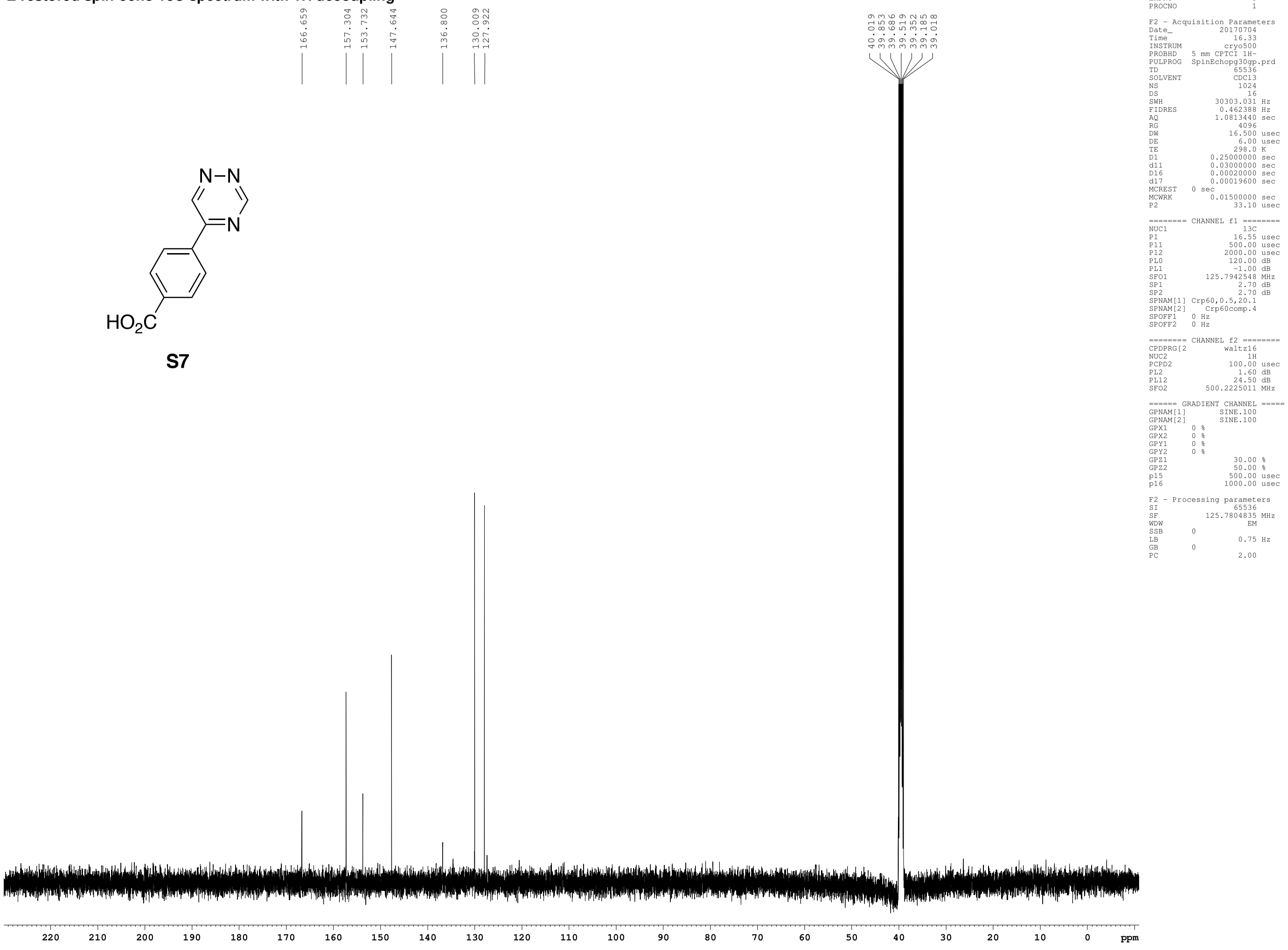

S7

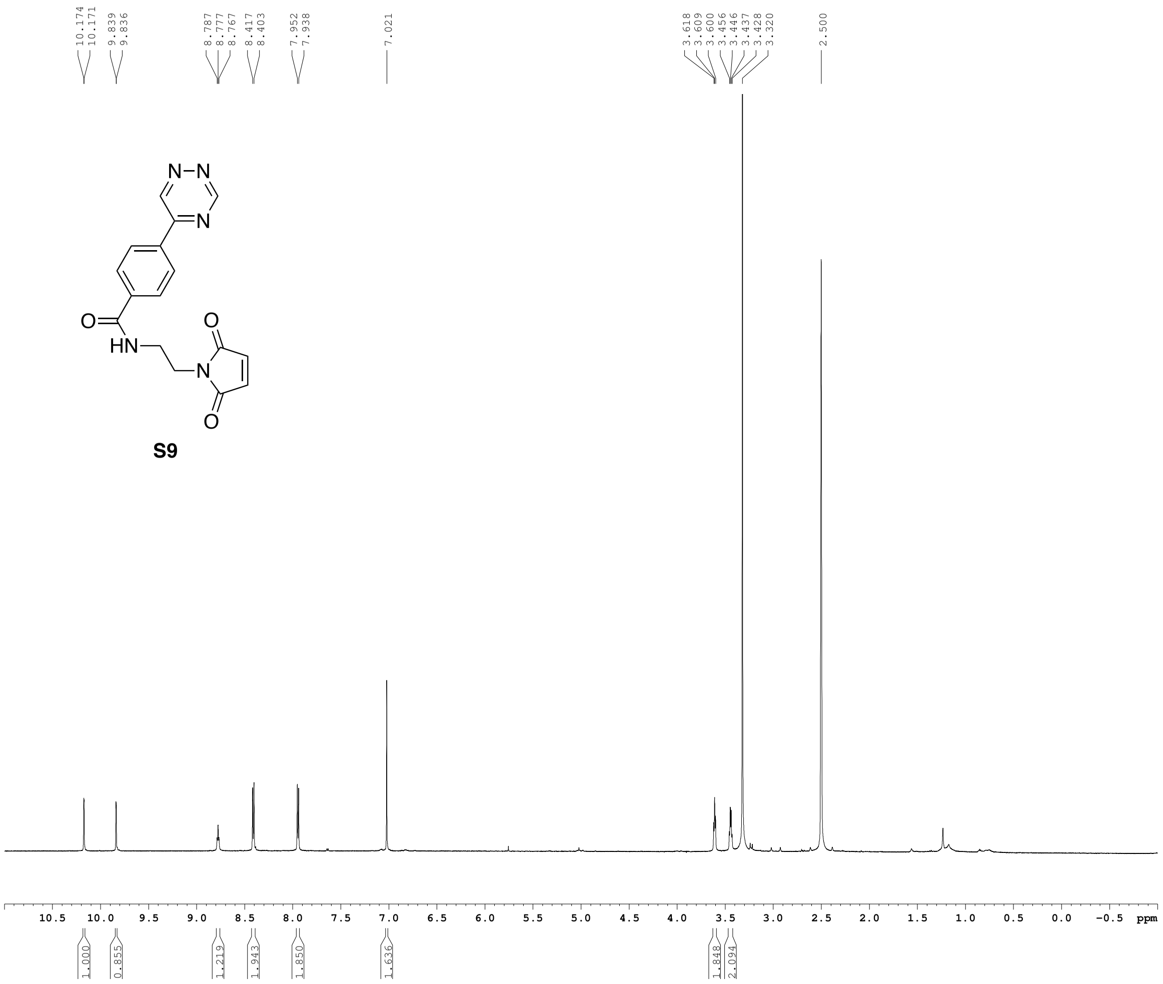

S9

```
Current Data Parameters
NAME              SN3-110
EXPNO                  10
PROCNO                  1

F2 - Acquisition Parameters
Date_            20170707
Time                 2.06
INSTRUM             av600
PROBHD   5 mm CPBBO BB-
PULPROG              zg30
TD                  98074
SOLVENT              DMSO
NS                      8
DS                      2
SWH              9615.385 Hz
FIDRES           0.098042 Hz
AQ              5.0998478 sec
RG                     10
DW                 52.000 usec
DE                  13.70 usec
TE                  298.0 K
D1             0.10000000 sec
TD0                     1

======== CHANNEL f1 ========
SFO1          600.1342009 MHz
NUC1                   1H
P1                  12.00 usec
PLW1          20.00000000 W

F2 - Processing parameters
SI                  65536
SF            600.1300130 MHz
WDW                    EM
SSB      0
LB                   0.30 Hz
GB       0
PC                   1.00
```

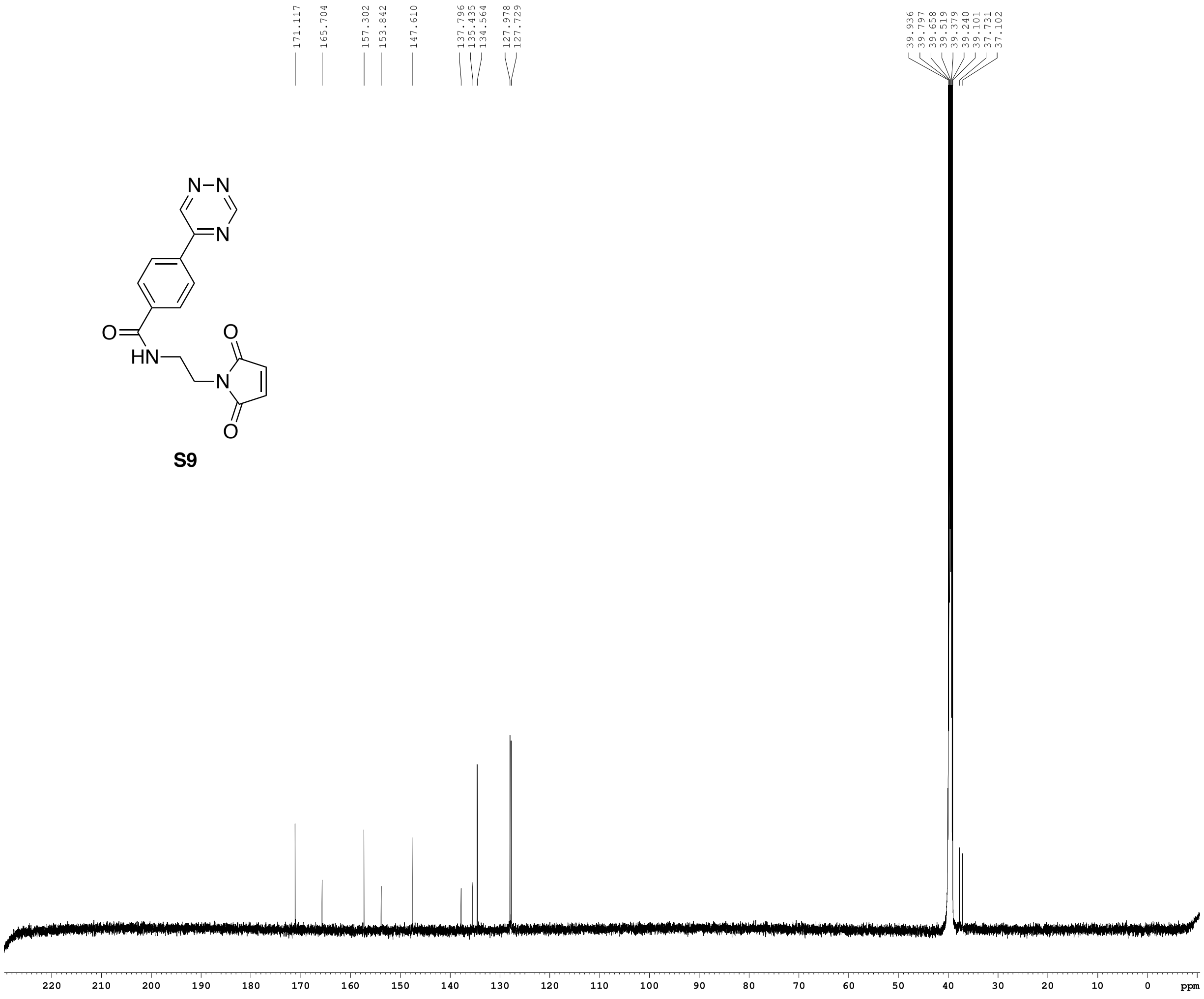

S9

Current Data Parameters		
NAME	SN3-110	
EXPNO	11	
PROCNO	1	
F2 - Acquisition Parameters		
Date_	20170707	
Time	2.15	
INSTRUM	av600	
PROBHD	5 mm CPBBO BB-	
PULPROG	zgdc30	
TD	65536	
SOLVENT	DMSO	
NS	958	
DS	4	
SWH	36231.883	Hz
FIDRES	0.552855	Hz
AQ	0.9043968	sec
RG	2050	
DW	13.800	usec
DE	19.65	usec
TE	298.0	K
D1	0.40000001	sec
D11	0.03000000	sec
TD0	1	
======== CHANNEL f1 ========		
SFO1	150.9194080	MHz
NUC1	13C	
P1	10.00	usec
PLW1	64.00000000	W
======== CHANNEL f2 ========		
SFO2	600.1330010	MHz
NUC2	1H	
CPDPRG[2	waltz16	
PCPD2	80.00	usec
PLW2	20.00000000	W
PLW12	0.36000001	W
F2 - Processing parameters		
SI	65536	
SF	150.9028830	MHz
WDW	EM	
SSB	0	
LB	1.00	Hz
GB	0	
PC	1.00	

1H spectrum

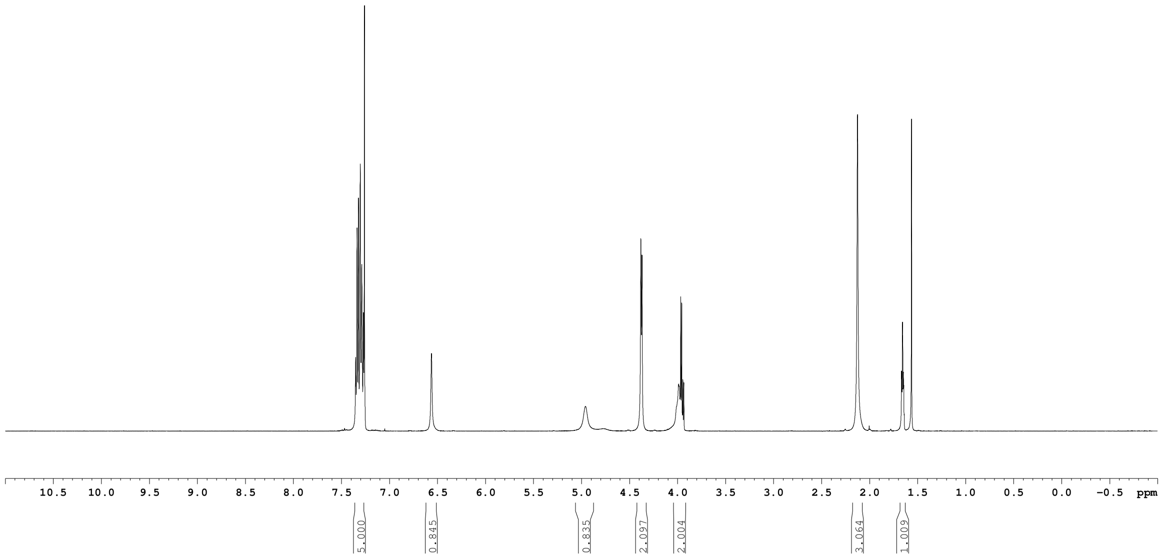

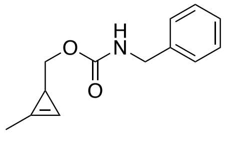

S11

```
Current Data Parameters
NAME              ZL2-025
EXPNO                   3
PROCNO                  1
DATPATH  /v/data/seansn/nmr

F2 - Acquisition Parameters
Date_            20181004
Time                 9.01
INSTRUM           cryo500
PROBHD   5 mm CPTCI 1H-
PULPROG              zg30
TD                  81728
SOLVENT             CDCl3
NS                     16
DS                      2
SWH              8012.820 Hz
FIDRES           0.098043 Hz
AQ              5.0998273 sec
RG                    7.1
DW                 62.400 usec
DE                   6.00 usec
TE                  298.0 K
D1             0.10000000 sec
MCREST   0 sec
MCWRK          0.01500000 sec

======== CHANNEL f1 ========
NUC1                   1H
P1                   7.50 usec
PL1                  1.60 dB
SFO1          500.2235015 MHz

F2 - Processing parameters
SI                  65536
SF            500.2200322 MHz
WDW                    EM
SSB      0
LB                   0.30 Hz
GB       0
PC                   1.00
```

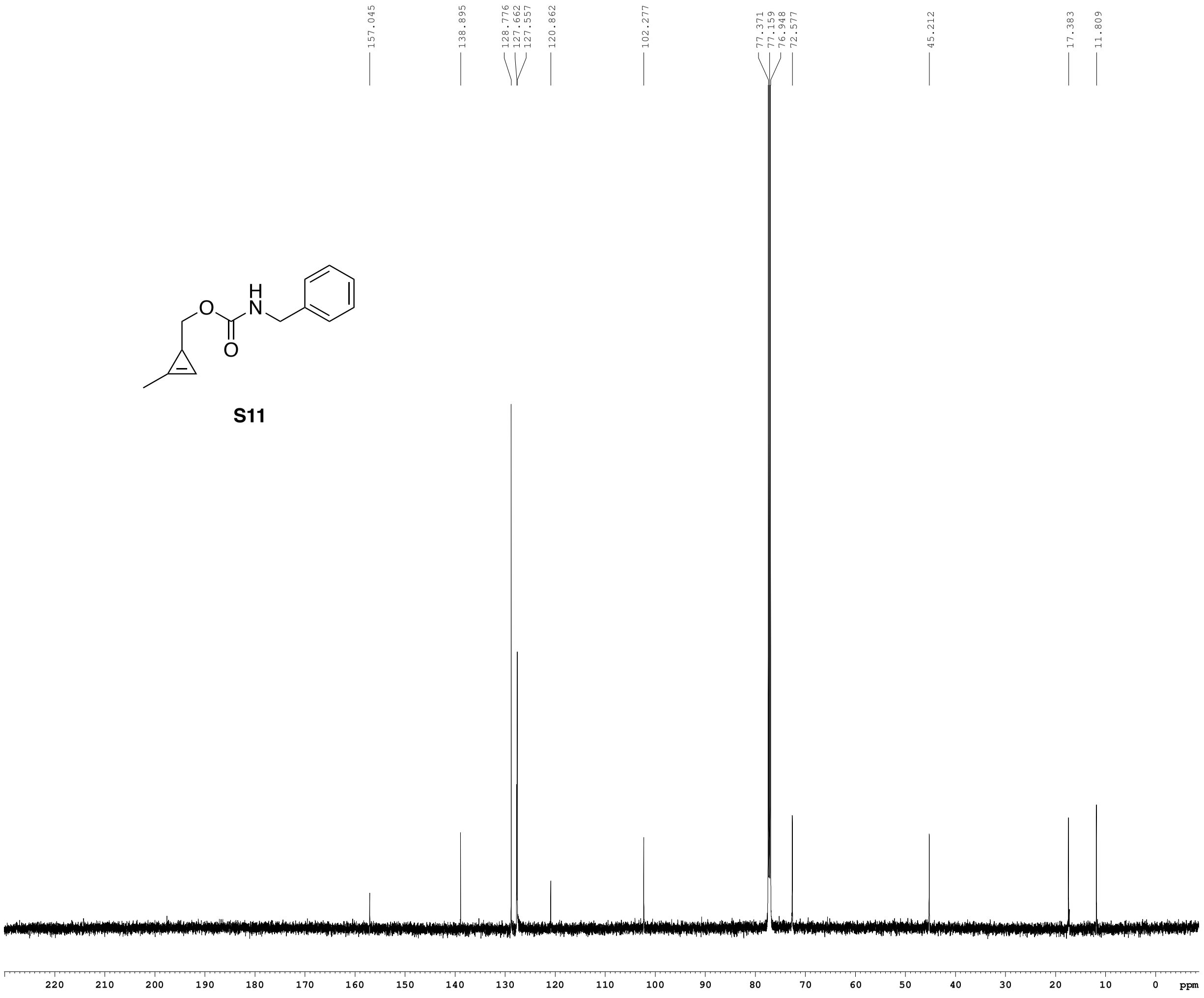


```
Current Data Parameters
NAME              ZL2-025
EXPNO                   2
PROCNO                  1
DATPATH  /v/data/seansn/nmr

F2 - Acquisition Parameters
Date_          20181004
Time               8.43
INSTRUM           av600
PROBHD   5 mm CPBBO BB-
PULPROG         zgdc30
TD               65536
SOLVENT          CDCl3
NS                 519
DS                   4
SWH          36231.883 Hz
FIDRES        0.552855 Hz
AQ           0.9043968 sec
RG                2050
DW              13.800 usec
DE               18.01 usec
TE               298.0 K
D1          0.40000001 sec
D11         0.03000000 sec
TD0                  1

======== CHANNEL f1 ========
SFO1       150.9194080 MHz
NUC1               13C
P1                9.60 usec
PLW1       64.00000000 W

======== CHANNEL f2 ========
SFO2       600.1330010 MHz
NUC2                1H
CPDPRG[2         waltz16
PCPD2            80.00 usec
PLW2       20.00000000 W
PLW12       0.32734001 W

F2 - Processing parameters
SI               65536
SF         150.9027936 MHz
WDW                 EM
SSB      0
LB                1.00 Hz
GB       0
PC                1.00
```

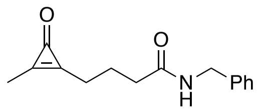

S13

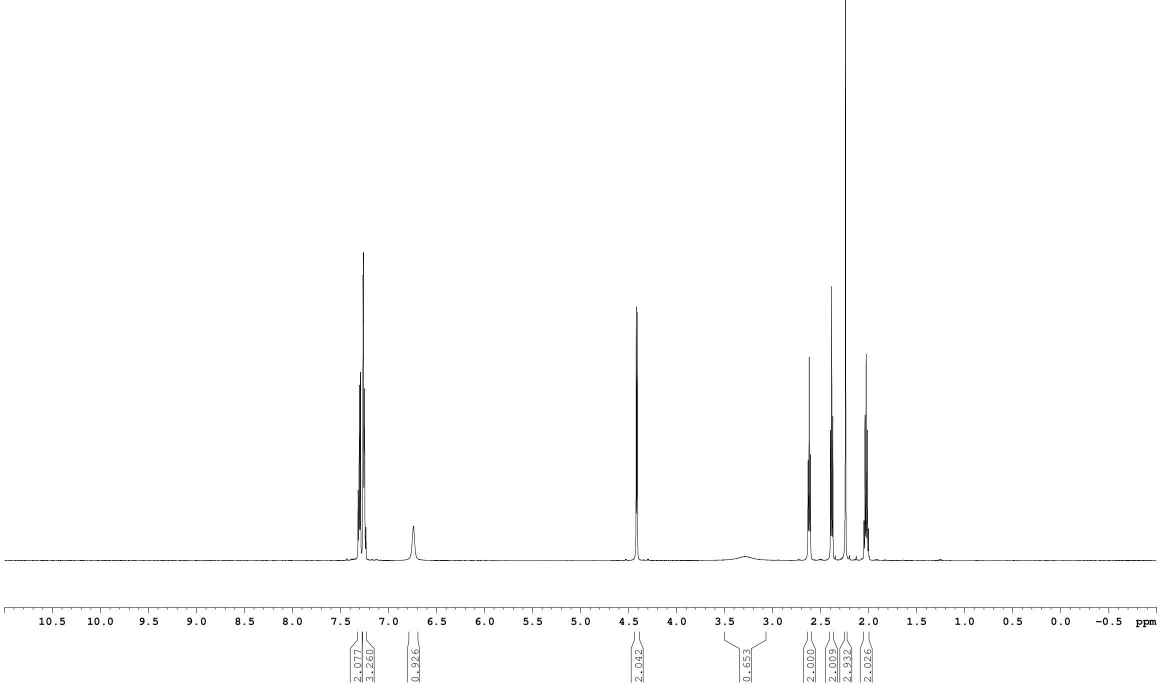

Current Data Parameters
NAME DR5-123
EXPNO 2
PROCNO 1
DATPATH /v/data/seansn/nmr

F2 - Acquisition Parameters
Date_ 20180913
Time 19.04
INSTRUM av600
PROBHD 5 mm CPBBO BB-
PULPROG zg30
TD 98074
SOLVENT CDCl3
NS 8
DS 2
SWH 9615.385 Hz
FIDRES 0.098042 Hz
AQ 5.0998478 sec
RG 10
DW 52.000 usec
DE 13.81 usec
TE 298.0 K
D1 0.10000000 sec
TD0 1

======== CHANNEL f1 ========
SFO1 600.1342009 MHz
NUC1 1H
P1 11.50 usec
PLW1 20.00000000 W

F2 - Processing parameters
SI 65536
SF 600.1300356 MHz
WDW EM
SSB 0
LB 0.30 Hz
GB 0
PC 1.00

13C spectrum with 1H decoupling

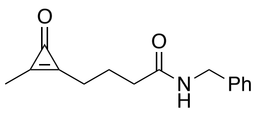

S13

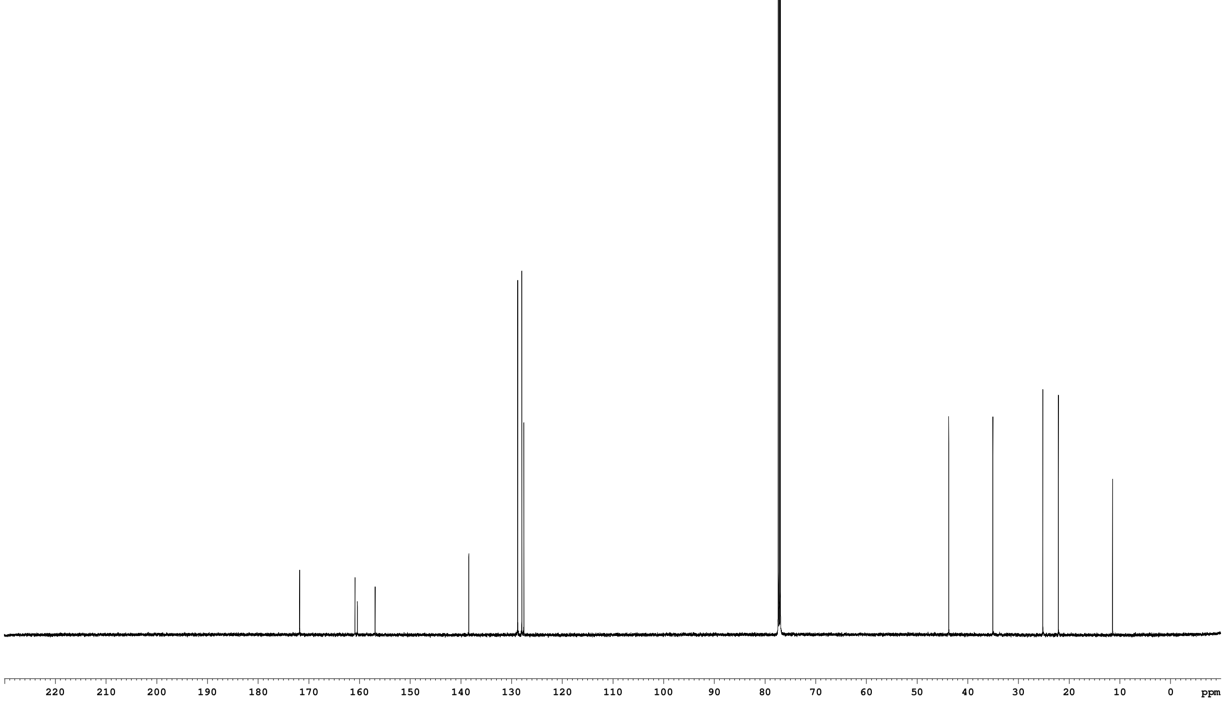


```
Current Data Parameters
NAME              DR5-123
EXPNO                   1
PROCNO                  1
DATPATH  /v/data/seansn/nmr

F2 - Acquisition Parameters
Date_            20180913
Time                19.10
INSTRUM             av600
PROBHD   5 mm CPBBO BB-
PULPROG           zgdc30
TD                  65536
SOLVENT             CDCl3
NS                    284
DS                      4
SWH             36231.883 Hz
FIDRES           0.552855 Hz
AQ              0.9043968 sec
RG                   2050
DW                 13.800 usec
DE                  18.01 usec
TE                  298.0 K
D1             0.40000001 sec
D11            0.03000000 sec
TD0                     1

======== CHANNEL f1 ========
SFO1         150.9194080 MHz
NUC1                  13C
P1                   9.60 usec
PLW1          64.00000000 W

======== CHANNEL f2 ========
SFO2         600.1330010 MHz
NUC2                   1H
CPDPRG[2          waltz16
PCPD2               80.00 usec
PLW2          20.00000000 W
PLW12          0.32734001 W

F2 - Processing parameters
SI                  65536
SF            150.9027974 MHz
WDW                    EM
SSB      0
LB                   1.00 Hz
GB       0
PC                   1.00
```
